# Topology‐Aware Deep Learning on Higher‐Order Structures for Drug Response Prediction

**DOI:** 10.1002/advs.75816

**Published:** 2026-05-27

**Authors:** Cong Shen, Guancen Lin, Chuan‐Shen Hu, Yong Wang

**Affiliations:** ^1^ State Key Laboratory of Mathematical Sciences, Academy of Mathematics and Systems Science Chinese Academy of Sciences Beijing China; ^2^ Department of Applied Mathematics National University of Kaohsiung Kaohsiung Taiwan; ^3^ School of Mathematics University of Chinese Academy of Sciences, Chinese Academy of Sciences Beijing China; ^4^ Center for Excellence in Animal Evolution and Genetics Chinese Academy of Sciences Kunming Yunnan China; ^5^ Key Laboratory of Systems Biology, Hangzhou Institute for Advanced Study University of Chinese Academy of Sciences, Chinese Academy of Sciences Hangzhou Zhejiang China

**Keywords:** drug response prediction, higher‐order interactions, interpretability, simplicial complexes, topological deep learning

## Abstract

Accurate prediction of anticancer drug response remains a central challenge in precision oncology. Existing approaches often rely on pairwise modeling, overlooking higher‐order dependencies among drugs and cell lines. We present TopDr, a topology‐aware deep learning framework that encodes both drugs and cell lines as multiscale simplicial complexes, capturing interactions at the 0‐, 1‐, and 2‐simplex levels. By jointly integrating local higher‐order neighborhoods and global topological structures, TopDr generates enriched representations for sensitivity prediction. Across six benchmark datasets, TopDr consistently matches or surpasses state‐of‐the‐art baselines in both regression and classification tasks. Beyond predictive accuracy, TopDr offers mechanism‐level interpretability: attention over 1‐ and 2‐simplices highlights drug pairs and triplets with significant pathway enrichment, while cell line groupings reveal biologically coherent expression modules. These results demonstrate that modeling multiscale higher‐order topology yields predictions that are not only accurate and robust but also biologically interpretable, paving the way for more reliable drug response modeling.

## Graphical abstract

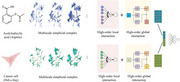

## 1 Introduction

Cancer remains one of the leading causes of death worldwide, and predicting how tumors or cancer cell lines will respond to therapeutic agents is crucial for precision oncology [1, 2]. Accurate prediction of drug response not only enables personalized treatment strategies for patients but also accelerates drug discovery and reduces the cost and time associated with experimental screening in preclinical models. Traditionally, cancer drug response has been measured through laboratory‐based assays such as in vitro viability tests (e.g. MTT, ${\mathrm{IC}}_{50}$) on cell lines, patient‐derived xenografts, or organoid models [3, 4]. While these assays provide valuable empirical data, they are often expensive, time‐consuming, and poorly scalable when screening hundreds or thousands of drug–sample combinations. Moreover, reproducibility can suffer across biological heterogeneity and experimental batches.

To overcome the limitations of experimental screening, a wide range of computational methods have been developed to predict drug response from molecular and genomic profiles [5]. The earliest approaches were based on quantitative structure–activity relationship (QSAR) models, which estimate drug activity by correlating handcrafted molecular descriptors with observed sensitivity values [6, 7]. While QSAR methods are intuitive and interpretable, they typically rely on simple linear assumptions and struggle to generalize to complex genomic contexts. Subsequently, machine learning models such as support vector machines, random forests, and ensemble regressors have been adopted to handle heterogeneous molecular data, including gene expression, mutation status, and copy number variation [2, 8, 9, 10, 11]. These models often incorporate drug and cell line similarity matrices to enhance generalization, but still depend heavily on manual feature design and lack the capacity to learn deep, nonlinear representations.

The advent of deep learning introduced more expressive architectures capable of automatically learning feature representations from high‐dimensional data. Multilayer perceptrons, convolutional neural networks, and variational autoencoders have been used to integrate drug molecular structures and transcriptomic profiles, achieving improved performance in predicting drug sensitivity across large‐scale pharmacogenomic datasets [12, 13, 14, 15, 16, 17]. However, these models generally treat drugs and cell lines as independent vectors, limiting their ability to model interactions between entities. To address this, graph neural networks (GNNs) have been proposed to exploit the relational structure between entities. In these models, drugs are represented as molecular graphs with atoms as nodes and bonds as edges, while cell lines may be embedded into similarity graphs or drug–cell interaction graphs [18, 19, 20, 21]. GNNs allow for localized message passing and structure‐aware feature aggregation, and have achieved state‐of‐the‐art performance in many benchmarks. Nonetheless, they mostly focus on pairwise interactions and often fail to capture higher‐order biological dependencies such as drug synergy or co‐regulated gene modules.

More recently, transfer learning techniques have been introduced to improve drug response prediction, especially in scenarios where annotated pharmacogenomic data are scarce. By pretraining models on large‐scale datasets and fine‐tuning them on smaller or domain‐specific datasets, transfer learning enables knowledge reuse across different biological contexts. For example, DeepCoVDR [22] leverages a graph transformer and cross‐attention mechanism to learn drug–cell line interactions, and uses transfer learning from cancer datasets to predict COVID‐19 drug responses. Similarly, CSG2A [23] incorporates a condition‐specific gene–gene attention mechanism to transfer knowledge from the LINCS L1000 transcriptomic dataset to GDSC cell line drug responses, achieving improved interpretability and accuracy. Another study [24] proposed a two‐step transfer learning strategy that significantly enhances predictive performance on small datasets by progressively fine‐tuning on related drug response tasks. Additional efforts, such as those [25, 26], extend these ideas through cross‐cell line and multi‐modal transfer learning frameworks that combine structural drug features and gene expression data from diverse biological systems. While these approaches mitigate data scarcity and domain shift, they remain largely confined to modeling pairwise drug–cell interactions. They often lack mechanisms to explicitly model the higher‐order and multiscale relationships that are pervasive in biological systems, such as drug–drug synergy, pathway crosstalk, or coordinated cellular responses. These limitations motivate the need for modeling paradigms that go beyond pairwise representations, and instead capture topological structures that reflect complex biological dependencies.

To address this gap, Topological data analysis (TDA), especially persistent homology and simplicial complex constructions, offers a mathematically principled framework to model multiscale and higher‐order relationships in data [27, 28, 29]. TDA has been successfully applied in oncology for biomarker discovery, disease subtyping, and treatment response prediction by extracting topological signatures (e.g., loops, voids) that persist across scales and correspond to biologically meaningful structure in omics data [30, 31, 32, 33, 34, 35]. In particular, at the molecular level, simplicial complexes represent interactions beyond pairwise edges, capturing motifs like triangles (3–way synergy) and higher‐dimensional holes, summarized by Betti numbers and persistence diagrams [36, 37, 38, 39]. Recent works have begun exploring topology‐aware and higher‐order models that move beyond graphs to simplicial complexes or hypergraphs [40, 41, 42, 43]. These include hypergraph neural networks [44, 45], simplicial neural networks [46], and other higher‐order message passing frameworks [47], which aim to capture multi‐way relationships beyond pairwise interactions. In parallel, transformer‐based and foundation‐model‐style approaches [48] have recently emerged, leveraging attention mechanisms to capture long‐range dependencies in molecular and pharmacogenomic data. However, these models typically focus on sequence or graph representations and do not explicitly model higher‐order topological structures across multiple scales.

TDA‐oriented methods that represent data using simplicial complexes and hypergraphs highlight the potential of higher‐order modeling for complex biological tasks. Recent studies have integrated rich topological structures, such as upper and lower adjacencies and (co)facial relationships among simplices or cells, into end‐to‐end deep learning models. For example, simplicial neural networks (SNNs), together with cell complex, CW complex, and hypergraph neural networks, extend graph neural networks by treating higher‐dimensional simplices, including edges, triangles, tetrahedra, and hyperedges, as fundamental computational units [44, 49, 50]. However, these approaches typically rely on a single predefined structure and focus primarily on local higher‐order message passing, while multiscale topological organization, long‐range dependencies, and closer integration with downstream prediction tasks remain less explored. Furthermore, current applications remain largely limited to social networks and atomic‐level molecular systems [38, 44, 45, 51, 52]. From a chemical space perspective, representing each molecule as a single node with associated chemical properties leaves the underlying topological structure and its relationship to drug–drug interactions insufficiently explored.

In this paper, we propose **TopDr**, a topology‐aware deep learning framework for cancer drug response prediction based on higher‐order structures. TopDr integrates topological modeling with neural networks to capture both local higher‐order motifs and global long‐range dependencies in drug and cell‐line similarity spaces. In TopDr, drugs and cell lines are each represented as simplicial complexes derived from molecular fingerprint similarity and gene expression similarity, respectively, capturing 0‐, 1‐, and 2‐simplices corresponding to entities, pairwise relationships, and triplet interactions. A dual‐branch neural architecture applies simplicial attention for local topological neighborhoods and Transformer–style self–attention to model long‐range dependencies within each simplex dimension. These multi–order representations are hierarchically fused and used to predict drug response. Compared to prior methods, TopDr explicitly models higher–order interactions, leverages robust and interpretable topological features, and achieves improved generalization and predictability in pharmacogenomic datasets.

## 2 Results

### 2.1 The Overall Framework of TopDr

**FIGURE 1:**
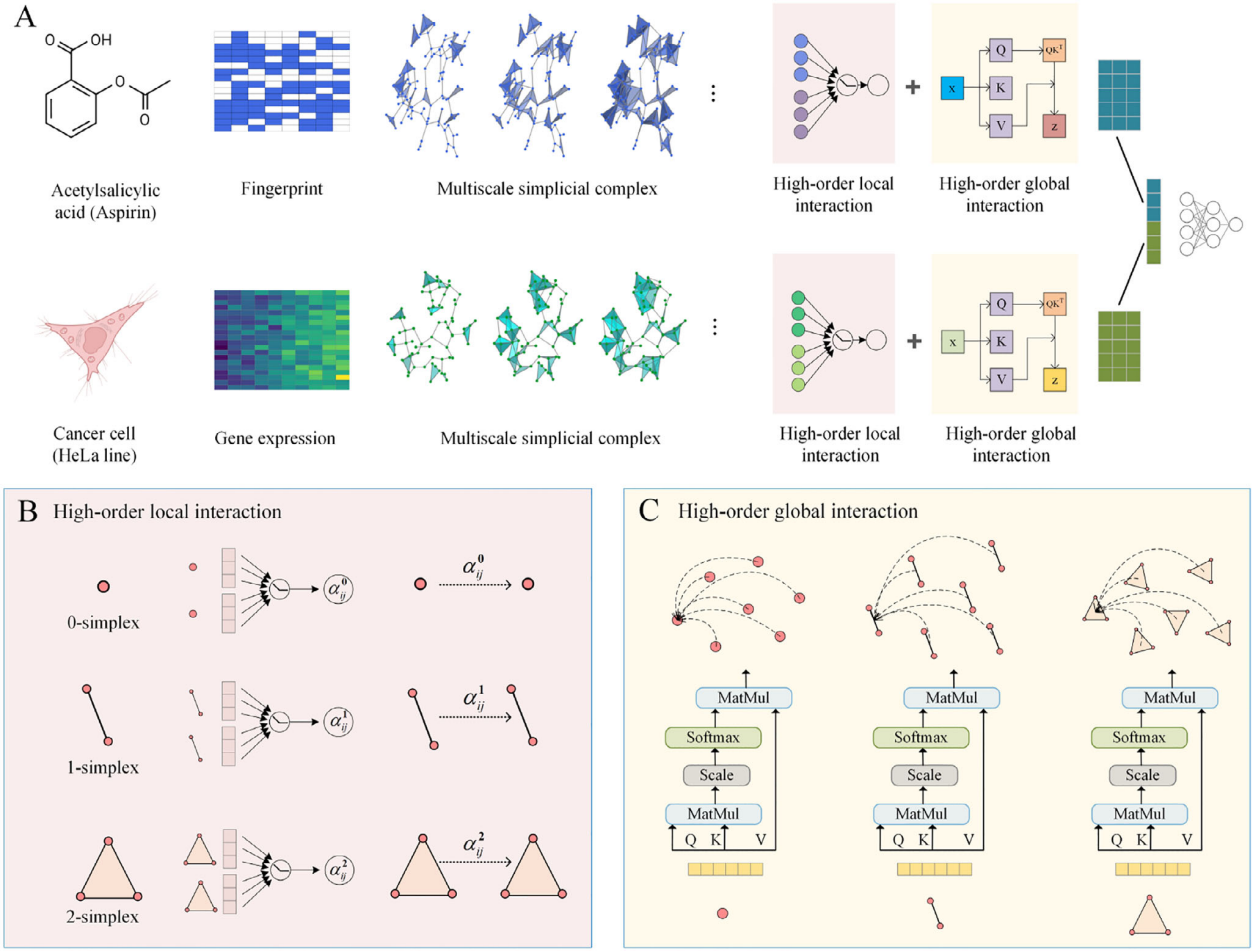
The overall framework of TopDr. (A) TopDr constructs multiscale simplicial complexes for both drugs and cell lines based on molecular fingerprints and gene expression similarity, respectively. Each simplicial complex captures 0‐, 1‐, and 2‐simplex relationships. A dual‐branch encoder is then applied to each complex: one branch models *high‐order local interactions* via attention‐based message passing across simplices of different dimensions, while the other branch captures *high‐order global interactions* using a Transformer‐style self‐attention mechanism. The fused embeddings from drugs and cell lines are used to predict drug response. (B) The high‐order local interaction encoder independently aggregates features from 0‐, 1‐, and 2‐simplices using simplex‐specific attention weights. (C) The high‐order global interaction encoder computes self‐attention among all simplices of the same dimension to capture long‐range dependencies.

TopDr is a topology‐aware deep learning framework that integrates higher‐order geometric structures into anticancer drug response prediction. As illustrated in Figure 1, the model comprises four major components: data representation, simplicial complex construction, topology‐aware encoding, and prediction (Figure 1A). First, each drug is represented by its molecular fingerprint, and each cell line by its gene expression profile. Based on the pairwise similarity matrices, TopDr constructs two independent multiscale simplicial complexes, where nodes (0‐simplices), edges (1‐simplices), and triangles (2‐simplices) represent similarity‐based connections among drugs or cell lines. A k‐nearest neighbor strategy is used to build the 1‐ and 2‐simplices.

Each simplicial complex is then encoded by a dual‐branch topology‐aware encoder. One branch models *high‐order local interactions* (Figure 1B) using a simplicial attention mechanism, which performs attention‐based message passing separately across the 0‐, 1‐, and 2‐simplices. The other branch models *high‐order global interactions* (Figure 1C) using a Transformer‐style self‐attention mechanism to capture long‐range dependencies among all simplices of the same dimension. The outputs of these two branches are fused to obtain enriched embeddings for drugs and cell lines. A cross‐attention module is subsequently applied to integrate the drug and cell line representations, learning drug–cell line compatibility in a context‐aware manner. The resulting fused features are passed through a fully connected network to predict the drug sensitivity score (e.g., ln(${\mathrm{IC}}_{50}$)). The entire model is trained end‐to‐end using mean squared error loss. By incorporating both local and global topological interactions over simplicial complexes, TopDr enables interpretable and accurate modeling of complex pharmacogenomic relationships.

### 2.2 Main Evaluation: Cancer Drug Response Prediction

To evaluate TopDr, we conducted experiments on six benchmark cancer drug response datasets: TGSA [53], GDSC1 [54], GDSC2 [54], CCLE [1], CTRP1 [55], and CTRP2 [55]. Unlike most existing work that focuses on splitting drug–cell line interaction pairs, we adopt a more practical and reproducible strategy by randomly partitioning the entire dataset–including all drug–cell line pairs–into 80% for training, 10% for validation, and 10% for testing. This ensures that the model is evaluated on previously unseen drug–cell line combinations, reflecting a realistic deployment scenario in which the system is required to generalize to novel therapeutic contexts.

We compare TopDr with 15 baseline methods spanning three representative categories. First, five state‐of‐the‐art models specifically designed for cancer drug response prediction are included: MultiDRP [56], MSDRP [57], PANCDR [58], GraphCDR [59], and SubCDR [60]. Second, five general‐purpose graph neural networks with strong empirical performance are considered: A‐DGN [61], ARMA [62], EGC [63], GraphGPS [64], and SSGC [65]. Third, four higher‐order or topology‐inspired methods are included: HGNN [44], ${\mathrm{HGNN}}^{+}$ [45], BScNets [46], and HiGCN [47]. In addition, we include MolGT [48] as a representative foundation model for molecular representation learning. These baselines enable a comprehensive evaluation of whether the proposed topological modeling framework provides consistent advantages over both conventional GNNs and higher‐order approaches. Performance is assessed using three standard metrics: Pearson Correlation Coefficient (PCC), Root Mean Square Error (RMSE), and Mean Absolute Error (MAE). All experiments are repeated five times with different random seeds, and the reported results correspond to the mean and standard deviation of the evaluation metrics.

**FIGURE 2:**
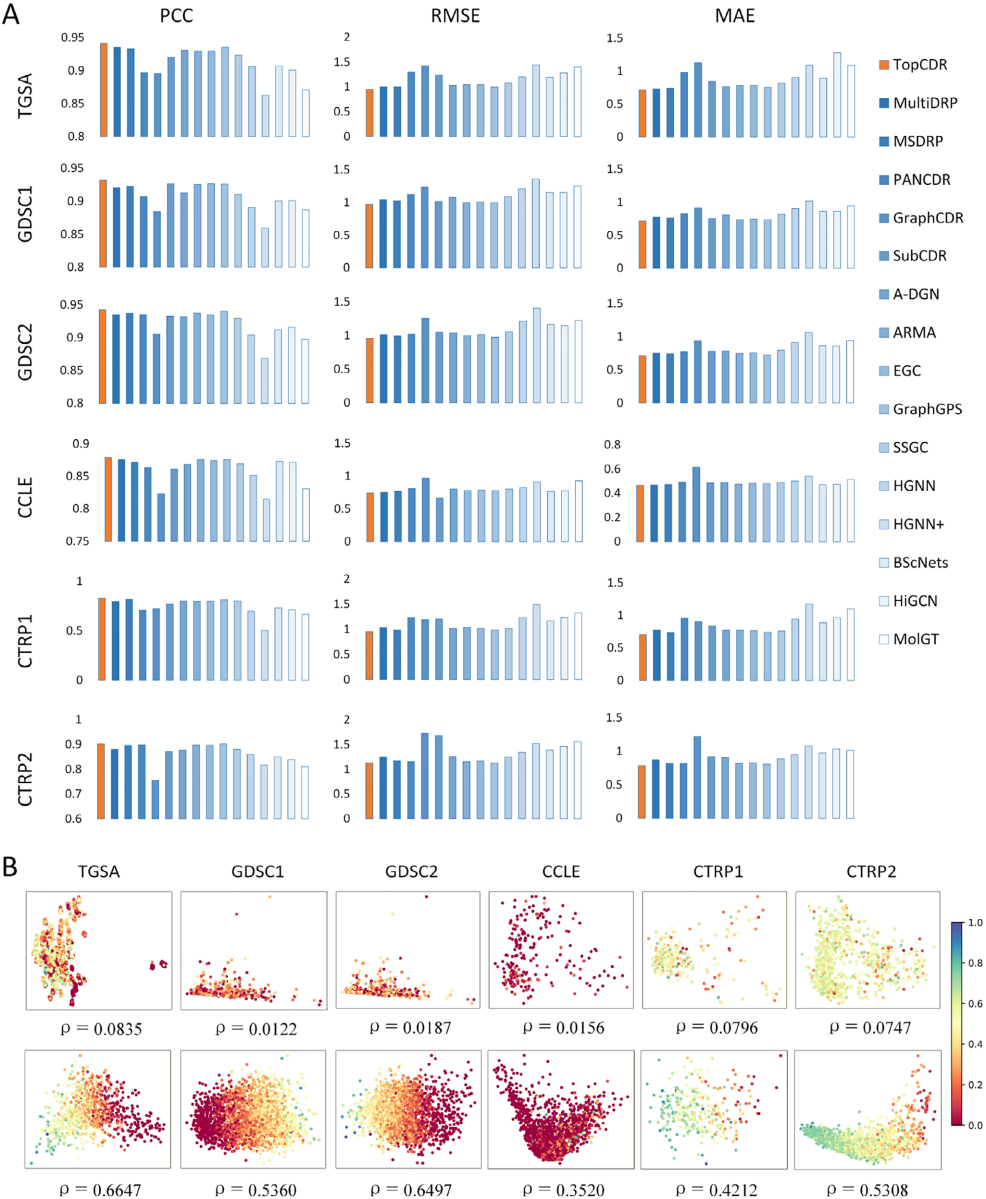
Main evaluation results of TopDr across six cancer drug response datasets. (A) Quantitative comparison of TopDr with ten baseline models on six benchmark datasets (TGSA, GDSC1, GDSC2, CCLE, CTRP1, and CTRP2), measured by Pearson Correlation Coefficient (PCC), Root Mean Squared Error (RMSE), and Mean Absolute Error (MAE). (B) Visualization of learned drug–cell line representations before (top) and after (bottom) applying TopDr using PCA. The color of each point corresponds to the associated drug response value, normalized to the range [0,1], and is independent of the PCA embedding.

As shown in Figure 2 and Table S1, TopDr consistently achieves the best or near‐best predictive performance across all datasets and evaluation metrics. For example, on the TGSA dataset, TopDr attains a PCC of 0.9418, outperforming strong baselines such as MultiDRP (0.9354) and MSDRP (0.9335), as well as recently introduced higher‐order methods including HGNN, ${\mathrm{HGNN}}^{+}$, BScNets, and HiGCN. It also achieves the lowest RMSE and MAE values of 0.9484 and 0.0.7114, respectively. On the GDSC2 dataset, TopDr again leads with a PCC of 0.9423 and an RMSE of 0.9608. Similarly, on CTRP2, TopDr attains a PCC of 0.9026 and an MAE of 0.7900, consistently surpassing both classical baselines and recently proposed topology‐inspired approaches. These improvements are consistent across all six benchmark datasets, where TopDr either ranks first or performs comparably to the top‐performing model within a statistically negligible margin. To further assess statistical robustness, we repeated all experiments five times with different random seeds and conducted paired *t*‐tests between TopDr and each baseline. As reported in Table S2, all p‐values are below 0.05, indicating that the observed performance gains are statistically significant. This demonstrates its strong generalization capability across diverse cancer types, drug classes, and omics modalities. Furthermore, TopDr exhibits clear advantages over both advanced graph neural network (GNN) architectures and higher‐order learning frameworks. Among the GNN baselines, GraphGPS shows the strongest performance, outperforming A‐DGN, ARMA, EGC, and SSGC across multiple datasets and metrics. GraphGPS, an advanced graph transformer variant, incorporates global structural information and positional encodings, and has demonstrated strong performance on large‐scale benchmarks. However, despite these advantages, its performance remains consistently inferior to that of TopDr on all six drug response prediction datasets. More importantly, although higher‐order and topology‐inspired models such as HGNN, ${\mathrm{HGNN}}^{+}$, BScNets, and HiGCN aim to capture complex interactions beyond pairwise relations, they still fall short of TopDr. This suggests that explicitly modeling multiscale topological structures, as done in TopDr, provides a more effective representation of the underlying biological systems. In addition, the foundation model MolGT, despite its strong pretraining capability, does not outperform TopDr in this task, indicating that domain‐specific topological inductive biases remain crucial for drug response prediction. Collectively, these results highlight TopDr not only as a powerful model for cancer drug response prediction but also as a robust framework for learning biologically meaningful and structurally informed representations.

In addition to predictive accuracy, we evaluate the quality of learned representations by projecting drug‐cell line embeddings into a two‐dimensional space using PCA (Figure 2B). The initial features (top row) exhibit weak structure and poor separability, whereas TopDr produces more coherent and well‐organized manifolds in which samples with similar drug response values are naturally clustered. To quantitatively assess this alignment, we compute the Spearman correlation between pairwise Euclidean distances in the 2D space and pairwise absolute differences in IC50 values. As shown in Figure 2B, the initial features yield very low correlations across datasets (e.g., ρ=0.0835 on TGSA and ρ=0.0122 on GDSC1), indicating poor consistency with the response landscape, while TopDr substantially improves this relationship (e.g., ρ=0.6647 on TGSA, ρ=0.5360 on GDSC1, and ρ=0.6497 on GDSC2). Similar improvements are observed on the remaining datasets, demonstrating that the learned embedding space better preserves the underlying structure of drug response. These results confirm that TopDr not only improves predictive performance but also learns more informative and biologically meaningful representations.

We attribute TopDr's superior performance to three major factors. First, the use of higher‐order topological constructs (1‐ and 2‐simplices) allows the model to capture beyond‐pairwise relationships, such as functional modules or drug combinations. Second, the multiscale modeling scheme allows TopDr to incorporate geometric information from multiple abstraction levels. Third, the combination of simplicial GAT and non‐local self‐attention enables TopDr to integrate both local and long‐range interactions, which are critical for modeling biological systems with rich hierarchical structure. Together, these innovations result in a robust, generalizable, and interpretable model for cancer drug response prediction.

### 2.3 Contribution of Multiscale and High‐Order Topological Objects

To further validate the design motivations behind TopDr, we conduct a series of ablation studies to systematically investigate the contributions of (1) multiscale neighborhood construction, (2) higher‐order topological structures (1‐simplex and 2‐simplex), and (3) global long‐range interactions. These components respectively capture local geometric context, simplicial topology beyond pairwise interactions, and non‐local dependencies, forming the core of our topological modeling framework. The results are summarized in Table 1.

**TABLE 1:** Ablation study of TopDr on six datasets under three evaluation metrics. For PCC, higher values indicate better performance, while for RMSE and MAE, lower values are preferred. The best results are highlighted in bold. Values in parentheses denote the standard deviation over five runs.

Metric	Methods	TGSA	GDSC1	GDSC2	CCLE	CTRP1	CTRP2
PCC	TopDr	**0.9418** 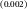	**0.9318** 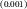	**0.9423** 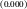	**0.8791** 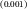	**0.8319** 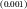	**0.9026** 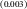
	w/o 0‐scale	0.9380 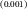	0.9299 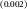	0.9417 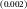	0.8788 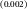	0.8221 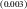	0.8921 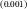
	w/o 1‐scale	0.9394 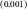	0.9290 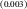	0.9401 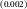	0.8772 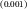	0.8175 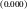	0.8988 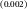
	w/o 2‐simplex	0.9400 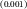	**0.9318** 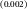	0.9396 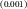	0.8704 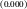	0.8221 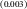	0.9022 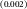
	w/o 1‐simplex	0.9396 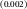	0.9297 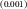	0.9404 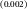	0.8782 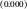	0.8262 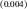	0.8981 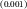
	w/o 1&2‐simplex	0.9407 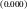	0.9296 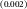	0.9403 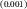	0.8781 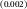	0.8309 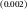	0.8973 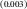
	w/o long range	0.9341 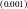	0.9247 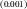	0.9320 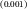	0.8738 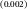	0.8168 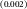	0.8856 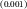
RMSE	TopDr	**0.9484** 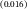	**0.9762** 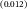	**0.9608** 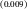	**0.7454** 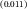	**0.9562** 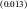	**1.1331** 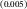
	w/o 0‐scale	0.9823 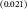	0.9883 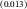	0.9700 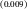	0.7530 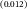	0.9740 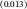	1.1877 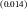
	w/o 1‐scale	0.9755 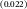	0.9766 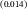	0.9773 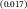	0.7506 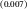	0.9924 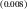	1.1513 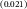
	w/o 2‐simplex	0.9674 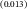	0.9767 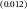	0.9784 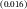	0.7477 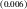	0.9775 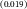	1.1353 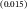
	w/o 1‐simplex	0.9691 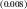	0.9886 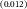	0.9694 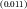	0.7481 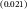	0.9707 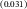	1.1564 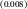
	w/o 1&2‐simplex	0.9626 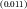	0.9870 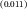	0.9704 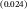	0.7743 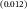	0.9660 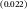	1.1586 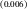
	w/o long range	1.0117 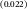	1.0201 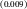	1.0360 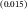	0.7628 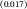	0.9950 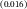	1.2203 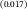
MAE	TopDr	**0.7114** 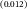	**0.7187** 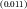	**0.7109** 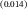	**0.4671** 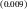	**0.7058** 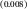	**0.7900** 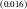
	w/o 0‐scale	0.7226 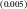	0.7310 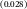	0.7143 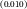	0.4690 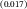	0.7127 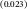	0.8328 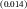
	w/o 1‐scale	0.7208 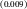	0.7221 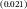	0.7198 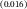	0.4693 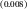	0.7282 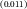	0.8052 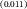
	w/o 2‐simplex	0.7146 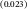	0.7196 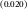	0.7220 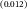	0.4675 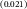	0.7234 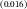	0.7928 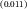
	w/o 1‐simplex	0.7155 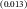	0.7307 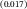	0.7176 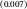	0.4673 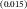	0.7079 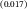	0.8090 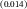
	w/o 1&2‐simplex	0.7151 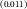	0.7275 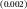	0.7195 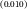	0.4716 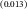	0.7079 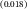	0.8071 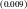
	w/o long range	0.7566 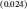	0.7602 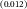	0.7752 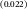	0.4774 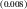	0.7342 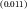	0.8650 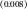

We first examine the role of multiscale neighborhoods by removing either the fine‐scale (5‐nearest neighbors, 0‐scale) or the coarse‐scale (10‐nearest neighbors, 1‐scale) adjacency. Removing either scale consistently degrades performance across datasets. For example, on TGSA, removing the 0‐scale increases RMSE from 0.9484 to 0.9823 and MAE from 0.7114 to 0.7226, while removing the 1‐scale leads to RMSE = 0.9755 and MAE = 0.7208. Similar trends are observed across other datasets, indicating that combining multiple neighborhood scales is essential for capturing complementary local structures.

We then evaluate the contribution of higher‐order simplicial structures. Removing 2‐simplices results in noticeable performance degradation (e.g., GDSC2 MAE increases from 0.7109 to 0.7220), while removing 1‐simplices often leads to comparable or slightly larger drops (e.g., GDSC1 MAE increases from 0.7187 to 0.7307). These results suggest that both pairwise and higher‐order interactions provide complementary structural information, and jointly contribute to accurate modeling of drug–cell relationships.

Finally, removing the long‐range interaction module leads to the most significant performance decline across all settings. For instance, on CTRP2, PCC drops from 0.9026 to 0.8856, while RMSE and MAE increase from 1.1331 and 0.7900 to 1.2203 and 0.8650, respectively. This highlights the critical role of global information propagation in capturing non‐local dependencies that cannot be recovered by local topology alone.

Overall, the ablation results consistently demonstrate that each component–multiscale construction, higher‐order topology, and long‐range interaction–contributes meaningfully to the final performance. Their integration enables TopDr to achieve robust and superior results across all datasets.

### 2.4 Biological Interpretability of Higher‐Order Simplicial Interactions

While TopDr demonstrates strong predictive performance, it is equally important to examine the biological interpretability of the learned representations. Specifically, we investigate whether the higher‐order topological structures encoded by the model correspond to known biological interactions and functional groupings. To this end, we conduct post hoc analyses focusing on the simplicial complexes constructed for drugs and cell lines. For each modality, we analyze the attention mechanisms, evaluate biological coherence via pathway enrichment, and assess whether the model captures meaningful higher‐order relationships that go beyond pairwise interactions.

#### 2.4.1 Higher‐Order Interaction Analysis in the Drug Simplicial Complex

We first focus on the drug simplicial complex derived from the TGSA dataset [53] to investigate whether the higher‐order attention patterns reflect biologically meaningful relationships among therapeutic agents. Our analysis spans 0‐, 1‐, and 2‐simplex levels to assess how topological representations contribute to interpretability in drug space.

**FIGURE 3:**
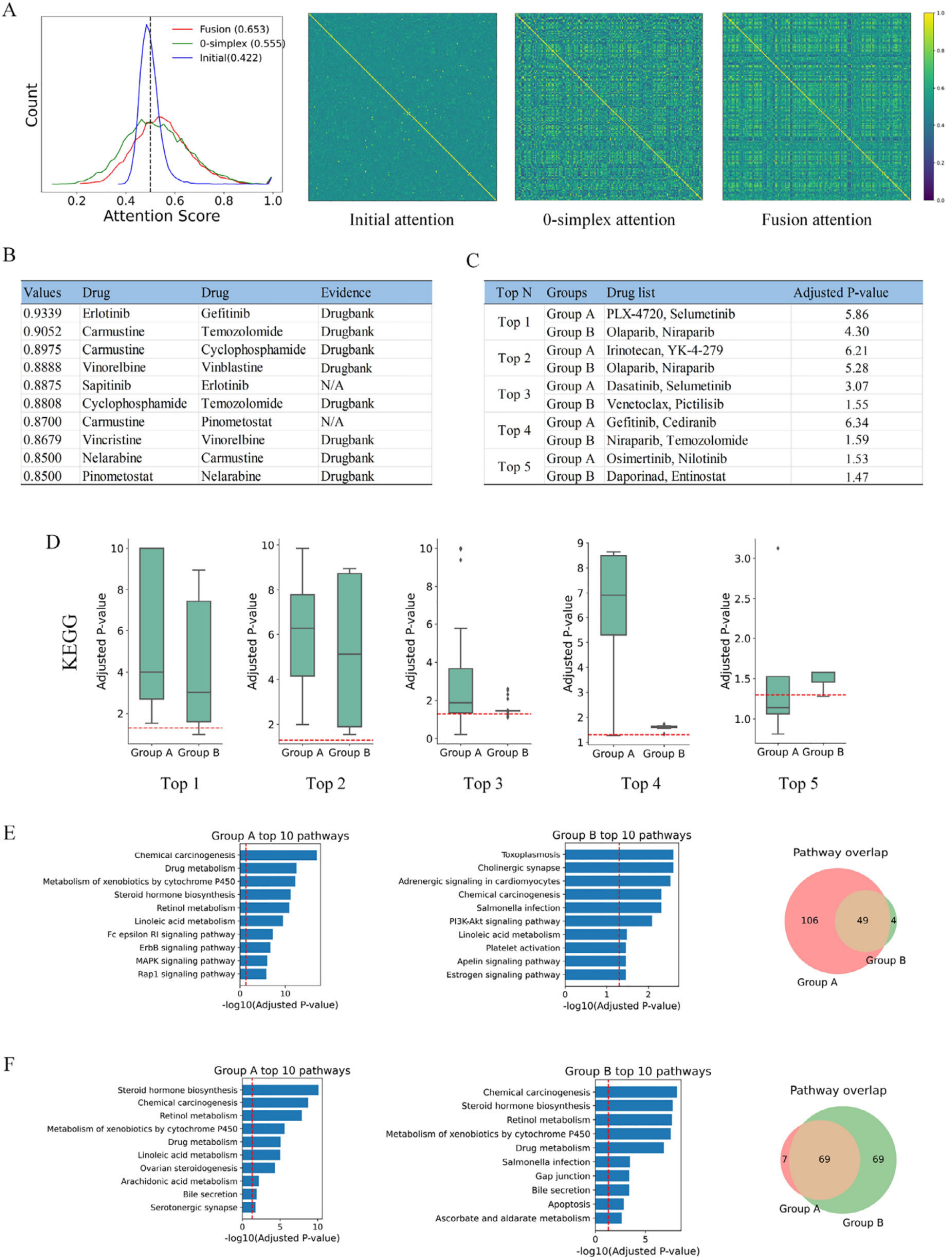
Biological interpretability of higher‐order drug interactions. (A) Distribution and visualization of three pairwise relation matrices for drugs at different stages: the initial similarity matrix computed from raw molecular fingerprints, the 0‐simplex score matrix computed from learned 0‐simplex representations, and the fusion score matrix computed from the final topology‐enriched representations. (B) Top 10 drug pairs ranked by fusion attention scores, with supporting evidence from DrugBank. (C) Enrichment significance ($-\log_{10}$(adjusted p)) of KEGG pathways for the top 5 drug pairs from 1‐simplex interactions. (D) Boxplots of adjusted p‐values for the top 5 1‐simplex drug pairs, showing statistical significance in KEGG enrichment (red dashed line = threshold 1.3). (E) Representative case: top 10 KEGG pathways enriched in Group A (Dasatinib, Selumetinib) and Group B (Venetoclax, Pictilisib), with pathway overlap shown as a Venn diagram. (F) Representative case: top 10 KEGG pathways enriched in Group A (Paclitaxel, Epirubicin, Docetaxel) and Group B (Vincristine, Irinotecan, Vinorelbine), with pathway overlap shown as a Venn diagram.

The interpretability analysis begins with attention matrices derived from the final drug representations. Specifically, we construct three pairwise relation matrices among all drugs (Figure 3A), each corresponding to a different stage of representation learning. (1) *Initial attention* denotes the pairwise similarity matrix computed directly from the raw molecular fingerprint features; (2) *0‐simplex attention* denotes the pairwise score matrix computed from the learned 0‐simplex representations after the 0‐simplex message‐passing stage, but before incorporating higher‐order information; and (3) *Fusion attention* denotes the pairwise score matrix computed from the final fused representations after aggregating 1‐ and 2‐simplex features. Thus, these three matrices reflect how drug‐drug relationships evolve from raw feature similarity to topology‐enriched learned representations. As shown in the left panel of Figure 3A, the proportion of attention values exceeding 0.5 is highest in the fusion attention matrix (65.3%), compared to 42.2% in the initial attention. This indicates that higher‐order structures enable TopDr to capture additional drug–drug relationships beyond those directly evident from chemical similarity. To validate whether these high‐scoring interactions are biologically meaningful, the top 10 drug pairs are extracted based on the fusion attention scores (Figure 3B). Eight out of the ten pairs are confirmed in the DrugBank interaction network.

To further assess the utility of higher‐order simplices, Pathway enrichment analysis is conducted on the drug targets involved in high‐attention 1‐ and 2‐simplex interactions. For 1‐simplex interactions, we select the top five drug pairs with the highest attention values and extract their associated targets. For each selected interaction (i.e., a drug pair or triplet identified by high attention scores), we define two groups, denoted as Group A and Group B, corresponding to the two sets of interacting entities within the simplex. These groups represent the endpoints of the learned higher‐order interaction, capturing distinct but functionally related subsets of drugs identified by the model. We then evaluate the functional similarity between the two groups in terms of enrichment significance, number of overlapping pathways, and Jaccard similarity across four gene set libraries (KEGG, GO‐BP, Reactome, WikiPathways).

As shown in Figure 3C and Table S3, most enrichment significance scores (i.e., $-\log_{10}$(adjusted p‐value)) exceed 1.3, and the overlapping pathway counts are consistently greater than 5, with Jaccard indices above 0.03 [66]. These trends strongly suggest that 1‐simplex interactions capture biologically coherent relationships between drugs. Figure 3D and Figure S1 further show the distribution of enrichment significance across all selected drug pairs, with a red dashed line indicating the significance threshold of 1.3. The consistently high enrichment scores ($-\log_{10}$(adjusted p)) reinforce the robustness of our observations. Additionally, Figure 3E illustrates a representative case based on the KEGG gene set, showing the top 10 enriched pathways for Group A (Dasatinib, Selumetinib) and Group B (Venetoclax, Pictilisib), along with a Venn diagram that highlights the extent of pathway overlap. To generalize this observation, Figure S2 presents similar analyses on the KEGG gene set for five drug group pairs, each accompanied by its corresponding Venn diagram. To further determine whether these observations go beyond random combinations, we constructed a random baseline by repeatedly sampling 1‐simplex pairs and computing the same enrichment statistics across the four gene set libraries. As summarized in Table S4, the random baseline yields markedly lower average enrichment significance (1.144), overlapping pathway counts (5.355), and Jaccard similarity (0.053), indicating that the top‐ranked 1‐simplex interactions identified by TopDr are substantially more functionally coherent than random simplex pairs.

Importantly, to further distinguish functional coherence from simple structural redundancy, we additionally examined high‐scoring 1‐simplex interactions with low cross‐group structural similarity (Table S5). Here, the structural similarity of an interaction pair was defined as the average cross‐group Tanimoto similarity between the molecular fingerprints of the two drug groups. Interestingly, several high‐scoring 1‐simplex pairs remained functionally coherent despite low structural similarity. These results indicate that the learned 1‐simplex interactions are not merely driven by chemical similarity, but can also uncover functionally related drug groupings that are structurally diverse.

The interpretability analysis is further extended to 2‐simplex interactions by selecting the top five drug triplets from the 2‐simplex attention matrix. As shown in Figure 3F, a representative example involving Group A (Paclitaxel, Epirubicin, Docetaxel) and Group B (Vincristine, Irinotecan, Vinorelbine) demonstrates statistically significant KEGG pathway enrichment, with all $-\log_{10}$(adjusted p) values exceeding 1.3. The Venn diagram illustrates substantial pathway overlap between the two groups. To generalize this observation, Figure S4 presents analogous results for five top‐ranked 2‐simplex drug group pairs, each showing consistent trends in pathway enrichment, overlap, and Jaccard similarity. Further, Figure S3 displays the distribution of enrichment significance across all selected triplets, with the red dashed line indicating the 1.3 threshold. The majority of drug combinations exceed this threshold, demonstrating the robustness of the enrichment. Table S6 summarizes the enrichment significance, number of overlapping pathways, and Jaccard similarity for each triplet pair, with most cases showing strong enrichment (>1.3), overlap counts greater than 5, and Jaccard indices above 0.03 [66]. For comparison, we also evaluated a random baseline by repeatedly sampling 2‐simplex pairs and computing the same pathway‐level statistics. As shown in Table S4, although random 2‐simplex pairs retain moderate pathway overlap on average, the top‐ranked 2‐simplex interactions identified by TopDr consistently exhibit stronger enrichment and higher functional coherence, supporting that the learned higher‐order interactions are not merely random combinations but biologically meaningful simplex‐level relations. To further verify that these identified higher‐order interactions are actively utilized by the model rather than being merely correlative, we conducted a targeted perturbation experiment by masking only the top‐ranked 2‐simplex interactions. Notably, masking only a few high‐scoring simplices leads to a more pronounced performance degradation (PCC drops from 0.9418 to 0.9391, RMSE increases to 0.9726) than removing all 2‐simplex interactions, indicating that TopDr relies on a small number of critical higher‐order structures as key decision‐making units. A similar phenomenon is also observed at the 2‐simplex level. For instance, the interaction between Group A (Paclitaxel, Epirubicin, Docetaxel) and Group B (Ribociclib, BI‐2536, Palbociclib)(Table S5), ranked 16th by interaction score, has a low structural similarity of 0.119, yet still shows strong functional coherence. This example further supports that TopDr is able to identify biologically meaningful higher‐order interactions beyond simple similarity‐based clustering.

#### 2.4.2 Higher‐Order Interaction Analysis in the Cell Line Simplicial Complex

Beyond drug interactions, we next turn to the cell line simplicial complex to assess whether similar biological coherence emerges in the representation of cellular profiles, using the TGSA dataset to examine interactions at the 0‐, 1‐, and 2‐simplex levels.

**FIGURE 4:**
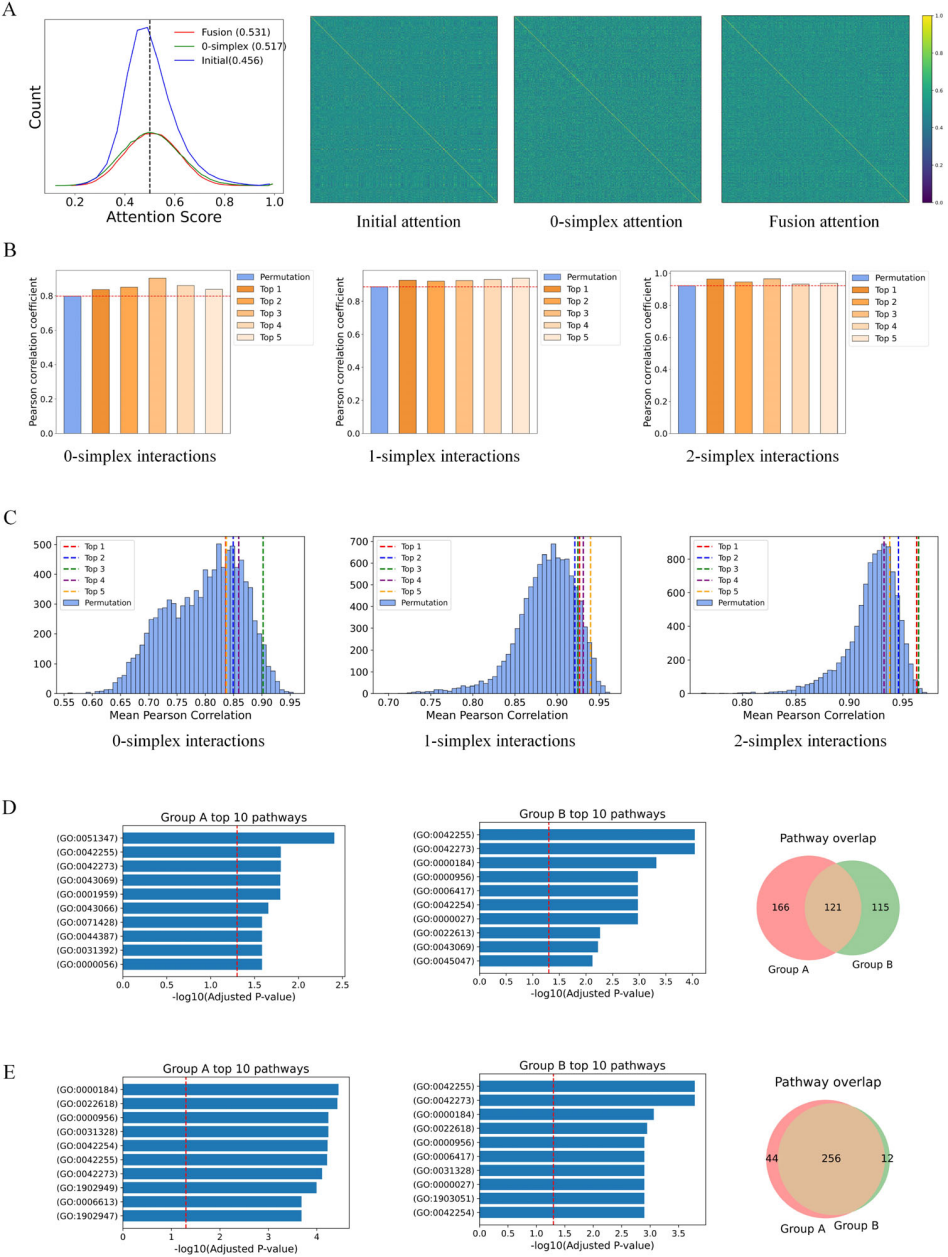
Biological interpretability of higher‐order cell line interactions. (A) Distribution and visualization of three pairwise relation matrices for cell lines at different stages: the initial similarity matrix computed from raw gene expression features, the 0‐simplex score matrix computed from learned 0‐simplex representations, and the fusion score matrix computed from the final topology‐enriched representations. (B) Bar plots comparing mean Pearson correlation coefficients between attention‐based groupings (Top 1–5) and permutation‐derived baselines across 0‐, 1‐, and 2‐simplex levels. (C) Null distributions of mean Pearson correlation coefficients from permutation tests, with true scores from Top 1–5 interactions marked by vertical dashed lines. (D) Representative 1‐simplex interaction: top 10 enriched GO terms in Group A (TYK‐nu, LU‐65) and Group B (Hs‐746T, SNG‐M), with overlapping pathways shown via Venn diagram. (E) Representative 2‐simplex interaction: top 10 enriched GO terms in Group A (NCI‐H1693, IST‐MES1, SNU‐C1) and Group B (HSC‐4, LS‐180, SW982), with corresponding pathway overlap.

Following a procedure analogous to that used for drug analysis, we construct three pairwise relation matrices among all cell lines (Figure 4A), corresponding to different stages of representation learning. (1) *Initial attention* denotes the pairwise similarity matrix computed directly from raw gene expression features; (2) *0‐simplex attention* denotes the pairwise score matrix computed from the learned 0‐simplex representations before incorporating higher‐order information; and (3) *Fusion attention* denotes the pairwise score matrix computed from the final fused representations after aggregating 1‐ and 2‐simplex features. We visualize the distribution of values in each matrix (Figure 4A, left panel), and observe that the Fusion attention matrix yields the highest proportion of scores above 0.5 (53.1%), compared to only 45.6% in the Initial attention matrix. This suggests that incorporating higher‐order topological information helps uncover novel and potentially meaningful relationships between celllines.

To rigorously test whether these inferred interactions correspond to biologically relevant patterns, we conduct a Permutation test using gene expression similarity as a surrogate for functional relatedness. For each attention matrix (0‐, 1‐, and 2‐simplex), we select the top five interaction pairs (or triplets) and denote them as Top 1 to Top 5. For each selected interaction, we define two groups, denoted as Groups A and B, corresponding to the two sets of interacting entities within the simplex. These groups represent the endpoints of the learned higher‐order interaction, capturing distinct but functionally related subsets of cell lines identified by the model. Then, we compute the mean Pearson correlation of gene expression between Groups A and B cell lines. Then, we generate a null distribution by randomly sampling pairs (or triplets) of cell lines to form new groups A and B, computing their average correlation score, and repeating this process 10 000 times. The results are shown in Figure 4B,C. The bar plots in Figure 4B compare the observed average correlation against the null mean (red dashed line), while Figure 4C shows the full null distributions with observed scores indicated by vertical dashed lines. In all cases, the true attention‐based groupings yield significantly higher expression similarity than expected by chance. These results hold across all simplex orders, indicating that both low‐ and high‐order interactions learned by TopDr are biologically plausible.

Beyond expression correlation, we also perform pathway enrichment analysis on the genes expressed in the interacting cell line groups. For 1‐simplex interactions, we analyze the top five attention‐ranked cell line pairs (Table S7), and for 2‐simplex interactions, the top five triplets (Table S8). For each pair or triplet, genes from Groups A and B are mapped to biological pathways across KEGG, GO‐BP, Reactome, and WikiPathways. We compute three metrics for each interaction: enrichment significance (adjusted p‐value, shown as ‐log10(p)), number of overlapping pathways, and Jaccard similarity. Most enrichment significance values exceed the threshold of 1.3, and the overlaps and Jaccard indices further support strong pathway‐level concordance between interacting groups. Finally, we showcase two representative examples from the 1‐simplex and 2‐simplex interaction lists. In Figure 4D, we present the top 10 enriched pathways for Group A (TYK‐nu, LU‐65) and Group B (Hs‐746T, SNG‐M), and in Figure 4E, we analyze the pathway enrichment of Group A (NCI‐H1693, IST‐MES1, SNU‐C1) versus Group B (HSC‐4, LS‐180, SW982). In both cases, adjusted p‐values for all top pathways exceed 1.3, confirming the functional coherence of the identified 1‐ and 2‐simplex interactions among cell lines.

Together, these analyses show that TopDr is capable of discovering biologically meaningful and statistically robust higher‐order relationships among cell lines. The results highlight the interpretability of our simplicial learning framework and its ability to capture both molecular and phenotypic structure embedded in gene expression data.

### 2.5 Robustness Under Feature Perturbation

To evaluate the robustness of our proposed TopDr model against noisy or incomplete input features, we conduct two additional experiments that simulate potential data corruption scenarios commonly encountered in real‐world biomedical settings. Specifically, we introduce controlled random perturbations to either the gene expression data of cell lines or the molecular fingerprints of drugs, and evaluate how predictive performance degrades across different models.

In the first experiment, we randomly replace 20% of the expression values in each cell line with uniformly distributed random noise. All other settings–including model architecture, training protocol, and data splits–remain identical to those in the main experiment. As shown in Table 2, TopDr consistently achieves the best performance across all six datasets under all three evaluation metrics (PCC, RMSE, and MAE). For example, on the GDSC2 dataset, TopDr attains a PCC of 0.9398, outperforming MultiDRP (0.9349) and MSDRP (0.9189), while simultaneously achieving the lowest RMSE (0.9687) and MAE (0.7233) among all compared methods. Similar trends can be observed across other datasets such as TGSA (PCC = 0.9396, RMSE = 0.9655, MAE = 0.7244) and CTRP2 (PCC = 0.9016, RMSE = 1.1521, MAE = 0.7988). These results demonstrate that TopDr maintains strong predictive accuracy even under substantial gene‐level perturbations, indicating high robustness to noisy expression inputs.

**TABLE 2:** Performance comparison under perturbed gene expression (20% random noise).

Metric	Methods	TGSA	GDSC1	GDSC2	CCLE	CTRP1	CTRP2
PCC	MultiDRP	0.9327 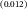	0.9242 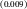	0.9349 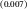	0.8747 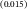	0.8153 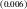	0.8992 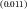
	MSDRP	0.9306 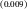	0.9249 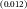	0.9189 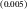	0.8744 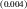	0.8161 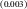	0.8897 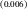
	PANCDR	0.8685 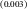	0.8793 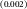	0.9317 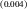	0.8428 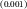	0.6729 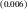	0.8867 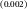
	GraphCDR	0.8992 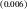	0.8820 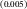	0.9062 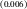	0.8226 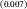	0.7293 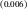	0.7545 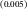
	SubCDR	0.9170 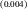	0.9243 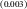	0.9317 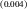	0.8596 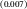	0.7664 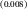	0.8686 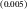
	A‐DGN	0.9213 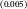	0.9126 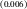	0.9289 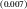	0.8702 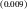	0.8014 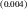	0.8762 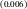
	ARMA	0.9305 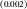	0.9290 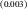	0.9347 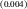	0.8739 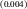	0.8061 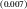	0.8961 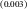
	EGC	0.9302 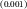	0.9247 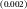	0.9344 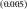	0.8768 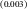	0.7973 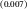	0.8941 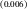
	GraphGPS	0.9250 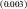	0.9288 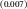	0.9192 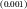	0.8773 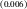	0.8109 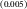	0.8788 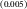
	SSGC	0.9241 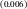	0.9122 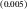	0.9284 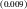	0.8705 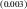	0.7962 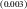	0.8785 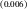
	HGNN	0.9064 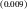	0.8950 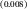	0.9101 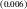	0.8538 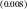	0.6709 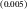	0.8647 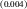
	${\mathrm{HGNN}}^{+}$	0.8712 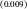	0.8706 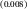	0.8750 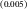	0.8196 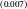	0.5070 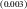	0.8256 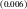
	BScNets	0.9038 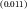	0.9009 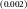	0.9138 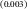	0.8710 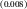	0.7321 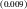	0.8470 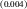
	HiGCN	0.9044 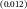	0.9031 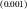	0.9167 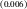	0.8728 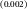	0.7292 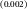	0.8401 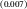
	MolGT	0.8657 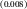	0.8655 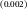	0.8935 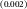	0.3805 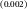	0.4759 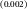	0.7157 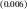
	TopDr	**0.9396** 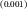	**0.9308** 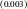	**0.9398** 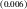	**0.8772** 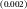	**0.8266** 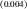	**0.9016** 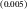
RMSE	MultiDRP	1.0219 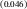	1.0238 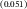	1.0119 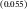	0.7594 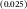	0.9982 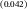	1.1881 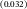
	MSDRP	1.0986 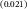	1.0125 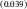	0.9780 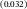	0.7646 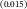	0.9959 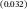	1.2055 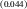
	PANCDR	1.4465 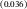	1.3449 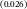	1.0411 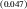	0.8646 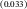	1.2788 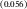	1.2192 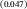
	GraphCDR	1.2414 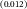	1.2493 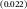	1.2205 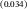	0.9237 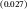	1.1840 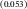	1.4493 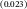
	SubCDR	1.2813 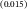	1.0448 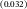	1.0647 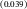	0.7967 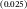	1.2651 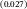	1.4217 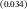
	A‐DGN	1.1030 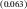	1.1026 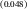	1.0563 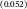	0.7708 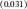	1.0224 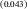	1.2667 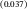
	ARMA	1.0395 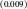	0.9936 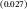	1.0138 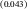	0.7622 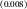	1.0127 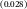	1.1721 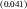
	EGC	1.0402 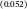	1.0038 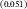	1.0154 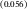	0.7516 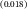	1.0359 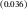	1.1764 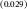
	GraphGPS	1.0053 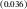	0.9945 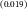	0.9850 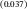	0.7527 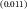	1.0045 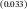	1.1931 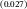
	SSGC	1.0778 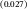	1.0971 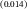	1.0602 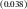	0.7720 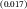	1.0341 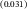	1.2552 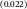
	HGNN	1.1963 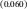	1.1872 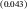	1.1773 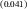	0.8214 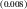	1.2785 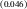	1.3274 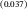
	${\mathrm{HGNN}}^{+}$	1.3929 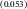	1.3151 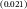	1.3753 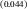	0.9028 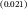	1.4869 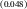	1.4964 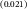
	BScNets	1.2123 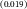	1.1575 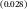	1.1543 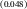	0.7739 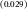	1.1735 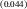	1.4055 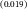
	HiGCN	1.2549 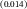	1.1458 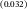	1.1392 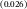	0.7689 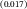	1.2216 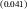	1.4592 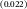
	MolGT	1.4114 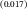	1.3585 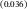	1.2901 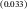	1.4636 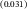	1.5160 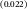	1.7373 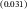
	TopDr	**0.9655** 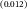	**0.9827** 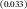	**0.9687** 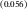	**0.7488** 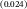	**0.9824** 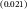	**1.1521** 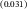
MAE	MultiDRP	0.7609 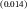	0.7625 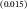	0.7541 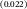	0.4751 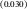	0.7333 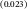	0.8004 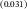
	MSDRP	0.7164 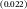	0.7536 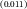	0.7729 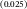	0.4795 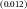	0.7403 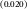	0.8369 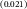
	PANCDR	1.0093 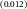	1.0123 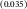	0.7904 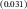	0.5265 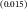	0.9917 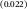	0.8608 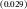
	GraphCDR	0.9266 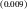	0.9273 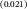	0.9195 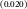	0.5568 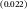	0.9020 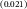	1.0533 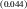
	SubCDR	0.8623 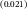	0.7681 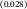	0.7780 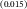	0.4895 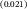	0.8455 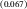	0.9295 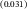
	A‐DGN	0.8236 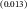	0.8249 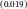	0.7945 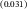	0.4826 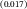	0.7669 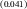	0.9080 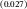
	ARMA	0.7690 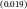	0.7378 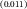	0.7518 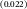	0.4761 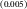	0.7646 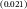	0.8270 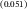
	EGC	0.7758 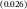	0.7471 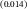	0.7593 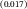	0.4729 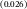	0.7797 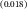	0.8342 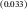
	GraphGPS	0.7451 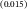	0.7397 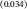	0.7368 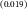	0.4721 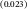	0.7501 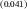	0.8221 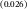
	SSGC	0.8119 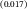	0.8210 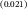	0.7977 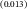	0.4848 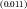	0.7825 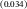	0.8895 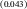
	HGNN	0.8903 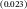	0.8872 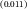	0.8781 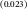	0.4982 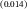	0.9824 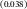	0.9277 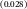
	${\mathrm{HGNN}}^{+}$	1.0550 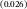	0.9906 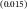	1.0369 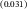	0.5366 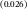	1.1792 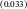	1.0703 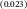
	BScNets	0.9103 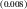	0.8623 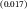	0.8612 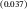	0.4722 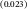	0.8859 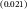	0.9830 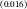
	HiGCN	0.9627 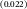	0.8541 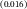	0.8515 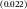	0.4711 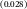	0.9486 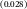	1.0279 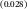
	MolGT	1.0971 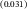	1.0484 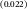	1.0039 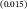	0.8101 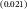	1.2032 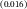	1.2997 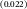
	TopDr	**0.7244** 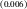	**0.7222** 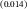	**0.7233** 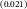	**0.4688** 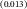	**0.7175** 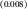	**0.7988** 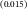

In the second experiment, we randomly perturb 20% of each drug's molecular fingerprint with random values, simulating incomplete or corrupted chemical descriptors. As shown in Table S9, TopDr again achieves the best overall performance across most datasets. For instance, on the TGSA dataset, TopDr achieves a PCC of 0.9401, outperforming MultiDRP (0.9323) and MSDRP (0.9088), while also yielding the lowest RMSE (0.9635) and MAE (0.7208). On GDSC2, TopDr achieves a PCC of 0.9397 with the lowest RMSE (0.9771) and MAE (0.7221), further confirming its stability under fingerprint perturbation. Notably, three drug‐specific baselines (PANCDR, GraphCDR, and SubCDR) are excluded from this experiment, as they rely on molecular graph structures rather than fingerprint representations. This highlights an additional advantage of TopDr: its ability to remain effective even when chemical descriptors are partially corrupted, which is challenging for models tightly coupled with specific structural encodings.

Taken together, these results demonstrate that TopDr exhibits strong robustness against both gene expression noise and molecular feature perturbations. The consistent performance gains suggest that incorporating multiscale, higher‐order, and non‐local topological representations enables the model to capture more stable and intrinsic structure patterns, thereby improving resilience under noisy or incomplete input conditions.

### 2.6 Cross‐Domain Generalization Performance

To comprehensively evaluate the generalization capability of the proposed TopDr model, we consider two challenging out‐of‐distribution settings: *cross‐cell line* and *cross‐drug* prediction. In the cross‐cell line setting, the dataset is split according to non‐overlapping cell lines, where 80% of cell lines are used for training, 10% for validation, and the remaining 10% for testing. In the cross‐drug setting, the split is instead performed over drugs, requiring the model to generalize to unseen compounds. Both settings reflect realistic scenarios in drug response prediction, where either new cell lines or new drugs are encountered.

**FIGURE 5:**
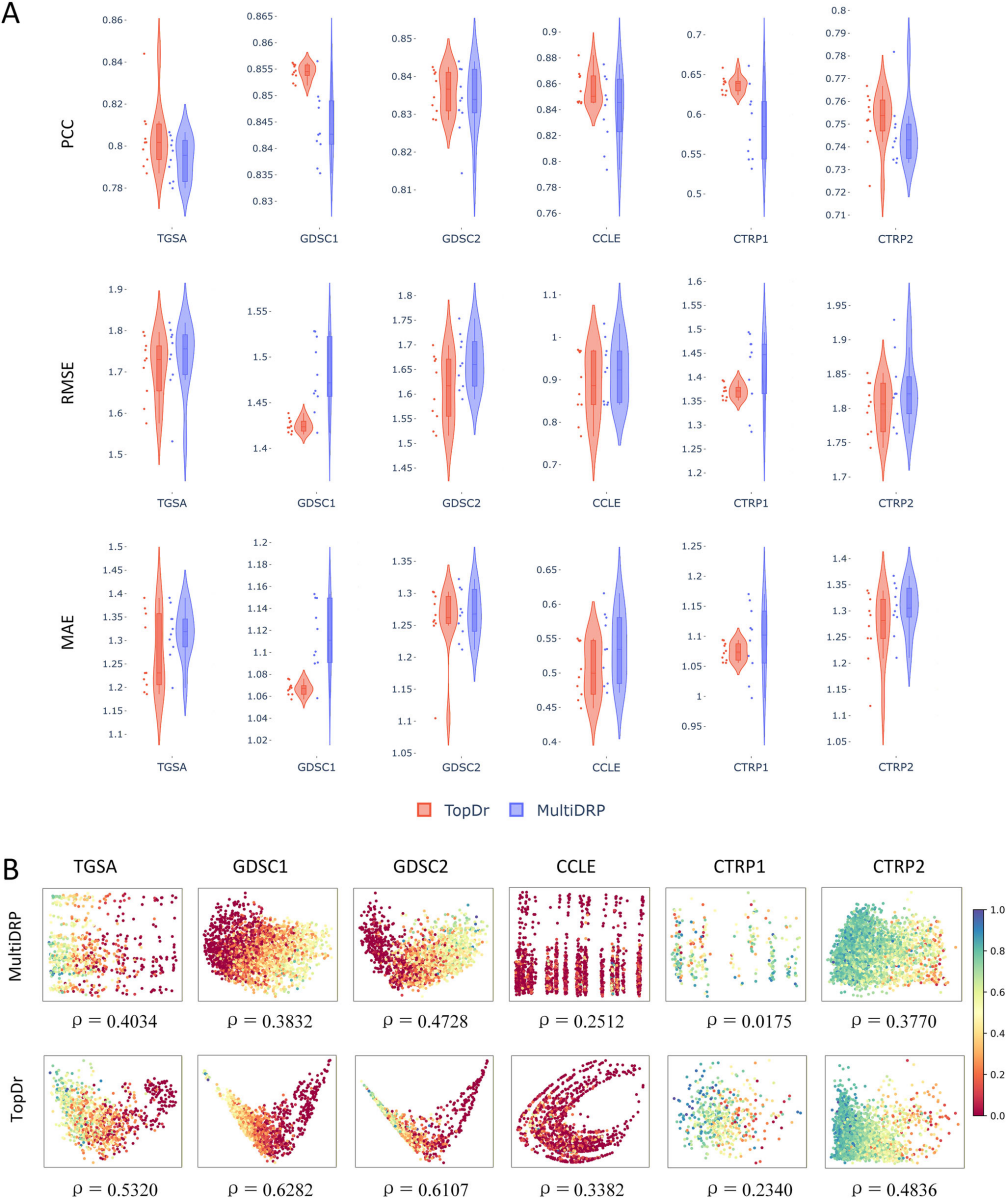
Cross‐cell Line Evaluation and Embedding Visualization of TopDr and MultiDRP. (A) Performance comparison between TopDr and MultiDRP across six benchmark datasets under a cross‐cell line split, evaluated using PCC, RMSE, and MAE. (B) Two‐dimensional projections of the learned drug–cell line embeddings by MultiDRP (top) and TopDr (bottom), where each point is colored according to the true drug response value. The color of each point corresponds to the associated drug response value, normalized to the range [0,1], and is independent of the PCA embedding.

As shown in Table S10 and Figure 5A, TopDr consistently outperforms the baseline MultiDRP across all six datasets under all three evaluation metrics in the cross‐cell line setting. For example, on GDSC2, TopDr achieves a PCC of 0.9370, substantially higher than MultiDRP (0.8336), while also reducing RMSE from 1.6649 to 1.6019 and MAE from 1.2674 to 1.2469. Similar improvements are observed on TGSA (PCC: 0.8072 vs. 0.7941) and CCLE (RMSE: 0.8783 vs. 0.9185; MAE: 0.4951 vs. 0.5365). Notably, on the more challenging CTRP1 dataset, TopDr improves PCC from 0.5871 to 0.6394, indicating stronger robustness under significant distribution shifts across cell lines. We note that the variance of performance differs across datasets in Figure 5A. This is likely due to differences in dataset heterogeneity and the sensitivity of the unseen‐cell line split, where some datasets exhibit stronger distribution shifts between training and testing cell lines than others. These results demonstrate that TopDr is able to learn transferable representations that generalize effectively to unseen cellular contexts.

The cross‐drug setting is inherently more difficult, as it requires extrapolation to unseen chemical structures. Despite this, TopDr consistently achieves better performance than MultiDRP across all datasets. For instance, on GDSC2, TopDr improves PCC from 0.6673 to 0.6855 while reducing RMSE from 2.1294 to 1.9946 and MAE from 1.6881 to 1.5923. On TGSA, PCC increases from 0.3925 to 0.4217, accompanied by notable reductions in RMSE (2.3513 → 2.2170) and MAE (1.6987 → 1.5358). Even on the most challenging dataset CTRP2, TopDr achieves a substantial reduction in RMSE (3.6905 to 3.0689) and MAE (2.3589 to 2.2655). These results indicate that TopDr exhibits stronger extrapolation capability and robustness when predicting responses for unseen drugs.

To further investigate the quality of the learned representations, we visualize the drug–cell embeddings in a two‐dimensional projection (Figure 5B), where each point is colored according to its true response value. Compared with MultiDRP, the embeddings learned by TopDr exhibit smoother and more structured manifolds that align more closely with continuous response gradients. This observation is further supported by higher Spearman correlation coefficients between pairwise distances in the embedding space and response differences (e.g., ρ=0.6282 vs. 0.3832 on GDSC1), indicating that TopDr captures a more meaningful geometric organization of drug response patterns.

The superior performance of TopDr in both settings can be attributed to its multiscale and higher‐order topological modeling. By leveraging simplicial complexes, TopDr captures multi‐way interactions among drugs and cell lines beyond pairwise relationships, while the integration of global and long‐range dependencies further enhances the robustness and transferability of the learned representations.

Overall, the results under both cross‐cell line and cross‐drug settings demonstrate that TopDr achieves strong generalization performance under distribution shifts, highlighting the effectiveness of topological and higher‐order modeling for robust cancer drug response prediction.

### 2.7 Modeling Discrete Drug Response Phenotypes via TopDr

To further evaluate the capability of TopDr in modeling discrete drug response phenotypes, we extend the analysis from continuous regression to a binary classification setting. In this setup, drug responses are binarized into sensitive and resistant categories, reflecting clinically relevant decision‐making scenarios. The experimental protocol follows the phenotype classification framework proposed in PANCDR [58].

Experiments are conducted on both the TCGA [67] and GDSC [68] datasets. Drug response values are converted into binary labels based on the median threshold, where samples below the median are considered sensitive and those above are labeled resistant. We compare TopDr with two representative baselines: PANCDR [58], a GAN‐based method, and DeepCDR [69], a multi‐modal deep learning framework. The evaluation metrics include AUC, accuracy (ACC), precision, recall, and F1 score.

The results are summarized in Figure S5. As shown in Figure S5A, TopDr achieves the best AUC on both datasets. On TCGA, TopDr obtains an AUC of 0.8206, significantly outperforming DeepCDR (0.7106) and PANCDR (0.5273). On GDSC, TopDr achieves an AUC of 0.8375, which is slightly higher than DeepCDR (0.8361) and notably better than PANCDR (0.7970). These results indicate that TopDr provides more reliable discrimination between sensitive and resistant samples.

Figure S5B further reports additional classification metrics. On the TCGA dataset, TopDr consistently achieves the best performance across all metrics, with ACC = 0.725, Precision = 0.734, Recall = 0.727, and F1 = 0.729, outperforming both PANCDR and DeepCDR by clear margins. On the GDSC dataset, TopDr attains the highest accuracy (0.881) and competitive F1 score (0.494), while maintaining a balanced trade‐off between precision and recall compared with the baselines. Notably, although DeepCDR exhibits relatively higher recall in some cases, its lower precision leads to inferior overall F1 performance, whereas TopDr achieves a more balanced and stable classification behavior.

Overall, these results demonstrate that TopDr not only excels in continuous drug response prediction but also generalizes effectively to discrete phenotype classification tasks. The consistent improvements across multiple metrics and datasets highlight the robustness of the learned representations, suggesting that the incorporation of multiscale and higher‐order topological structures enables TopDr to capture more discriminative patterns in pharmacogenomic data.

### 2.8 Cancer‐Specific vs. Pan‐Cancer Drug Response Prediction

An important practical question is whether drug response prediction should be performed in a cancer‐specific manner or under a pan‐cancer setting. The former focuses on a single cancer type and may better capture its intrinsic response patterns, while the latter leverages samples from multiple cancer types and evaluates generalization to a specific target cancer.

We consider three representative cancer types: BRCA (breast cancer susceptibility gene, including BRCA1/2, commonly used to denote breast cancer), LUAD (lung adenocarcinoma), and COREAD (colorectal cancer). In the cancer‐specific setting, for each cancer type, only the corresponding subset of samples is used and split into training, validation, and test sets with a ratio of 8:1:1. In the pan‐cancer setting, the target cancer type is used only for testing, while all remaining samples are used for training and validation with a ratio of 9:1.

The results are reported in Table 3. Overall, both methods achieve better performance in the cancer‐specific setting than in the pan‐cancer setting, indicating that drug response patterns are highly cancer‐type dependent. In addition, TopDr consistently outperforms MultiDRP across all cancer types and both settings in terms of PCC and error metrics.

**TABLE 3:** Performance comparison between cancer‐specific and pan‐cancer settings.

Cancer type	Method	Cancer‐specific			Pan‐cancer		
		PCC	RMSE	MAE	PCC	RMSE	MAE
BRCA	MultiDRP	0.8947 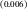	1.1784 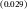	0.8780 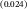	0.7544 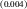	1.7742 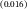	1.3236 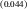
	TopDr	0.9041 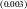	1.1173 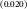	0.8397 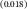	0.7651 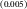	1.7444 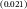	1.3297 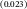
LUAD	MultiDRP	0.9167 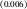	1.1111 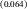	0.8448 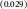	0.8042 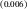	1.7868 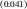	1.3794 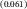
	TopDr	0.9273 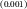	1.0795 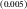	0.8109 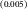	0.8101 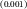	1.6906 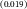	1.2950 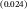
COREAD	MultiDRP	0.9109 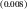	1.1409 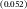	0.8520 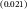	0.8204 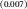	1.7148 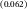	1.3208 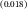
	TopDr	0.9204 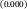	1.0846 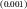	0.8225 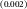	0.8284 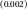	1.6956 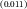	1.3331 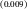

These results suggest that cancer‐specific modeling is more suitable for accurate prediction within a given cancer type, while TopDr provides a more robust representation that generalizes better across different cancer contexts.

### 2.9 Hyperparameter Sensitivity Analysis

To validate the robustness of the proposed TopDr framework with respect to topology construction and feature initialization, we conduct a comprehensive sensitivity analysis over key hyperparameters, including the number of nearest neighbors k in kNN graph construction, the RBF center range $c_{l}$, and the step size in RBF encoding. The results are summarized in Tables S11–S14.

We first evaluate the impact of the number of nearest neighbors k used in constructing the underlying graph structure. As shown in Table S11, the performance remains stable across a wide range of k values (from 5 to 20), indicating that the proposed method is not highly sensitive to this parameter. Smaller values, such as k=5 tend to better preserve local geometric structures, while moderately larger values such as k=10 improve graph connectivity and facilitate the propagation of global information. In contrast, larger values (k≥15) lead to denser graphs and higher‐order complexes, which may introduce redundant connections and increase computational cost without consistent performance gains. Therefore, we adopt a multi‐scale strategy using k=5 and k=10 to balance local fidelity and global context.

We further analyze the influence of the RBF center range used for distance encoding. For 1‐simplex features (Table S12), a wider range (e.g., [0,10]) achieves consistently strong performance across datasets, as it better captures the broader distribution of pairwise distances. In contrast, for 2‐simplex features (Table S13), the optimal performance is achieved with a relatively narrower range (e.g., [0,4]). This difference reflects the distinct geometric roles of different simplex orders: 1‐simplex encodes pairwise relationships with larger variation in distances, while 2‐simplex captures local higher‐order interactions, where distances are more concentrated. These results justify the use of different RBF ranges for different simplex orders.

Finally, we evaluate the effect of the RBF step size (Table S14). The results show that the model performance is stable across a wide range of step sizes (0.05–0.5), demonstrating the robustness of the proposed encoding scheme. Among all configurations, a step size of 0.1 achieves the most balanced performance across PCC, RMSE, and MAE. From a modeling perspective, smaller step sizes may introduce redundant features due to overly dense RBF centers, while larger step sizes reduce the resolution of distance encoding. A step size of 0.1, therefore, provides a suitable trade‐off between representation capacity and smoothness.

Overall, these results demonstrate that TopDr is robust to hyperparameter choices in both topology construction and feature encoding. The selected configuration (k=5 and k=10, RBF ranges of [0,10] for 1‐simplex and [0,4] for 2‐simplex, and step size of 0.1) provides a consistent balance between performance, interpretability, and computational efficiency. Importantly, the stability across all tested configurations suggests that the performance gains of TopDr do not rely on delicate hyperparameter tuning, but instead stem from the intrinsic expressiveness of the proposed topology‐aware representation.

## 3 Discussion

Predicting cancer drug response is a central problem in precision oncology, with wide‐ranging implications for patient stratification and personalized treatment planning. Existing computational approaches have made significant strides by leveraging omics data and graph‐based representations; however, they often fall short in capturing the complex, high‐order relationships inherent in biological systems. To address this challenge, we propose TopDr, a novel framework that integrates topological deep learning with simplicial complex representations of drugs and cell lines. By moving beyond conventional pairwise modeling, TopDr is designed to capture multi‐way interactions and multiscale structural features in the pharmacogenomic landscape. Extensive experiments across six benchmark datasets show that TopDr consistently achieves competitive or superior performance in both regression‐based sensitivity prediction and phenotype‐level classification tasks, highlighting its versatility and robustness.

The core innovation of TopDr lies in its use of higher‐order topological modeling through simplicial complexes. Unlike traditional graph neural networks that operate on pairwise interactions, our approach explicitly encodes and learns from 1‐simplex and 2‐simplex structures, allowing it to capture cooperative patterns among drug combinations and cell lines. Additionally, TopDr integrates information at multiple scales by fusing representations across simplex orders, enhancing the model's capacity to learn both local and global features. To ensure biological relevance, we construct topological structures using domain‐specific priors, including chemical similarity and gene expression correlation, thereby aligning the representation space with known pharmacological and cellular biology.

To validate both predictive power and interpretability, we conducted a series of comprehensive experiments. In regression tasks, TopDr significantly outperformed strong baselines such as MultiDRP and MSDRP across metrics and datasets (see Figure 2 and Table S1). Notably, our higher‐order interpretability analysis revealed that the top‐attended 1‐ and 2‐simplex drug interactions are biologically meaningful: most of them exhibit pathway enrichment scores above the statistical threshold ($-\log_{10}\left(p_{\mathrm{adj}}\right)>1.3$), with consistent overlap and Jaccard indices across KEGG and Reactome gene sets (see Figure 3, Tables S3 and S6). Similar findings hold for the cell line simplicial complex, where top‐attended groups show significantly higher gene expression correlations than expected by chance (Figure 4). These results demonstrate that TopDr not only delivers predictive accuracy but also provides insights into higher‐order biological relationships among drugs and cell lines.

Despite its strong performance, TopDr has several limitations. First, the current simplicial complex construction relies on pre‐defined similarity thresholds, which may introduce sensitivity to hyperparameter settings. In addition, the current RBF‐based distance encoding is primarily an engineering design and is not yet explicitly linked to biological scales. Second, our model assumes static topologies across all samples, potentially overlooking patient‐specific network rewiring. Third, TopDr does not yet incorporate external omics modalities (e.g., methylation, mutation), which could further enhance its biological fidelity. In addition, the current framework has limited zero‐shot generalization capability. Future work may therefore explore adaptive topology learning, dynamic simplicial architectures, multi‐omics integration, as well as strategies for improving cross‐dataset transferability, such as domain adaptation, distribution alignment, and large‐scale pretraining, to enable more robust zero‐shot or few‐shot generalization.

In summary, TopDr presents a powerful and interpretable framework for cancer drug response modeling. By introducing a topological perspective into the predictive pipeline, it bridges the gap between high‐order data structures and actionable prediction in pharmacogenomics. We anticipate that TopDr can be readily applied to other domains where multi‐agent and high‐order interactions are essential. In particular, it can be extended to drug synergy prediction by explicitly modeling higher‐order interactions among multiple drugs and cell lines, as well as to multi‐omics integration by representing different omics modalities within a unified higher‐order topological framework. All code and data are available at https://github.com/CS‐BIO/TopDr.

## 4 Materials and Methods

### 4.1 Dataset and Data Pre‐Processing

Six widely‐used pharmacogenomic datasets–TGSA, CCLE, GDSC1, GDSC2, CTRP1, CTRP2–were employed in this study. All datasets underwent systematic preprocessing to ensure consistency in cell line expression profiles, drug molecular representations, and response measurements. For each dataset, drug–cell line response matrices were constructed based on IC_50_, AUC, or LFC values, and filtered for samples with complete information on gene expression, drug response, and molecular structure.

#### 4.1.1 TGSA

The TGSA (Target‐Gene Synergy Analysis) dataset [53] contains 580 cancer cell lines and 170 anticancer compounds, covering 82,833 drug–cell line response pairs based on IC_50_. All drugs are associated with canonical SMILES strings. Gene expression profiles span 706 landmark genes.

#### 4.1.2 CCLE

The Cancer Cell Line Encyclopedia (CCLE) dataset [1] provides gene expression data for 1,037 cancer cell lines and 18,988 genes, retrieved from the DepMap portal (DepMap 24Q2; https://depmap.org/portal). The top 1000 genes by median expression were retained. Drug response values, obtained from the PRISM Repurposing Public 24Q2 release, are reported as LFC ($\log_{2}$ fold change) across 6790 drugs and 919 cell lines. Drugs without valid SMILES (retrieved via PubChem) were discarded. After intersecting gene expression and response data, 1,267 drugs and 221 cell lines remained.

#### 4.1.3 CTRP1

The CTRP v1 dataset [55] includes drug response data for 203 small molecules across 243 cell lines, with area under the dose curve (AUC) as the sensitivity metric. SMILES strings are directly provided in the dataset. Cell line expression profiles were sourced from CCLE and restricted to the top 1,000 genes. After intersecting expression and response data, 203 drugs and 240 cell lines were retained.

#### 4.1.4 CTRP2

The CTRP v2 dataset [55], obtained from the Broad Institute (http://www.broadinstitute.org/ctrp), contains AUC responses for 545 drugs across 887 cell lines. SMILES strings for all compounds are included. Gene expression profiles were obtained from CCLE. After intersecting modalities, 545 drugs and 821 cell lines were included in the final dataset.

#### 4.1.5 GDSC1 and GDSC2

Both datasets were downloaded from the Genomics of Drug Sensitivity in Cancer (GDSC) database [2, 54, 68] (https://www.cancerrxgene.org/). GDSC1 originally includes 970 cell lines and 378 drugs, while GDSC2 includes 970 cell lines and 286 drugs. Drug responses are reported as fitted IC_50_ values. RNA‐seq expression matrices provide read counts and RSEM‐derived values; the rsem_expected_count column was selected. The 1,000 most highly expressed genes across cell lines were retained. Drug SMILES were retrieved from PubChem; drugs without valid molecular representations were excluded. After filtering and intersecting all modalities, GDSC1 retained 312 drugs and 946 cell lines, while GDSC2 retained 233 drugs and 944 cell lines.

#### 4.1.6 Data Harmonization

For datasets reporting IC_50_ values, GDSC1, GDSC2, and TGSA all used natural logarithmic transformation (ln), and the response values were provided in the LN_IC50 format. This is consistent with the standard in the GDSC database and was adopted directly by TGSA. AUC values (CTRP1/2) were used in their original form, as provided in the dataset, and LFC values (CCLE) were used as‐is. In all datasets, outliers beyond three standard deviations from the mean were clipped. Only samples with complete expression, response, and molecular structure information were retained. Final dataset dimensions are summarized in Table S15.

### 4.2 Multiscale Topological Representation for Drug and Cell Line

Understanding the structure–function relationship of biomolecules is crucial for elucidating how their shape, flexibility, and interactions influence key biological processes. Beyond representing atoms and chemical bonds in biomolecules with graph models, advanced topological constructs, such as *simplicial complexes*, *cell complexes*, and *hypergraphs*, provide a more comprehensive framework for encoding and analyzing intrinsic molecular structures. Mathematically, while cell complexes and hypergraphs provide flexible and efficient representations for higher‐dimensional groupings of data points, determining these groups and their face relations often relies on additional assumptions, such as K‐nearest neighbors clustering [44]. By contrast, simplicial complexes capture all combinatorial and face relationships among data points without requiring such assumptions. Accordingly, we adopt simplicial complex representations to encode the topological structure of the data in this work.

#### 4.2.1 Simplicial Complex Representation

As a generalization of a graph structure, which consists of vertices and edges as its 0‐ and 1‐dimensional elements, an *(abstract) simplicial complex* extends this framework to include higher‐dimensional objects called *simplices*. Mathematically, given a non‐empty finite set $V=\{v_{1},v_{2},\text{…},v_{n}\}$ with n∈N, an (abstract) simplicial complex over V is a collection Δ of non‐empty subsets of V such that, for every σ∈Δ and every non‐empty subset τ⊆σ, we have τ∈Δ. A simplex σ∈Δ is called a q‐*simplex* if |σ|=q+1, where |σ| denotes the cardinality of σ. In this context, the conventional vertices and edges of a graph can be viewed as 0‐simplices and 1‐simplices, respectively. Additionally, 2‐simplices and 3‐simplices represent, respectively, triangles and tetrahedra, while higher‐dimensional simplices, defined analogously, abstractly encode higher‐order interactions among vertices in V. In particular, by fixing a total order $v_{1}<v_{2}<\cdots <v_{n}$ on V, every q‐simplex σ can be uniquely expressed as the ordered sequence $\left[v_{i_{0}},v_{i_{1}},\text{…},v_{i_{q}}\right]$, called an *oriented* q‐*simplex*, where $v_{i_{0}},v_{i_{1}},\text{…},v_{i_{q}}\in V$ and $v_{i_{0}}<v_{i_{1}}<\cdots <v_{i_{q}}$. The set of all q‐simplices of Δ is denoted by $\Delta_{\left(q\right)}$, and a simplex $\sigma \in \Delta_{\left(q\right)}$ is also written as $\sigma^{q}$ to indicate its dimension.

Analogous to the relationship between an edge and its endpoints in a graph, a *face relation* between simplices is defined in this setting. Specifically, a p‐simplex $\tau^{p}=\left[w_{0},w_{1},\cdots ,w_{p}\right]$ is called an p‐*face* of a q‐simplex $\sigma^{q}=\left[v_{0},v_{1},\cdots ,v_{q}\right]$ if $\left\{w_{0},w_{1},\cdots ,w_{p}\right\}\subseteq \left\{v_{0},v_{1},\cdots ,v_{q}\right\}$. In this context, a q‐simplex $\sigma^{q}=\left[v_{0},v_{1},\cdots ,v_{q}\right]$ has exactly q+1 faces of dimension q−1. Namely, all (q−1)‐faces of $\sigma^{q}$ can explicitly represented by

$$\left[\hat{v_{0}},v_{1},\text{…},v_{q}\right],\;\left[v_{0},\hat{v_{1}},\text{…},v_{q}\right],\;\text{…}\;,\;\left[v_{0},v_{1},\text{…},\hat{v_{q}}\right],$$

where $\left[v_{0},\cdots ,\hat{v_{i}},\cdots ,v_{q}\right]$ denotes the oriented (q−1)‐simplex obtained by removing vertex $v_{i}$. We define two types of adjacency relations between simplices. Two q‐simplices $\sigma^{q}$ and $\tau^{q}$ are said to be *upper adjacent*, denoted $\sigma^{q}⌣\tau^{q}$, if they are both faces of a common (q+1)‐simplex. They are said to be *lower adjacent*, denoted $\sigma^{q}⌣\tau^{q}$, if they share a common (q−1)‐face. In particular, for each q‐simplex $\sigma \in \Delta_{\left(q\right)}$, we define its *neighborhood* $N^{\left(q\right)}\left(\sigma \right)$ as the set of q‐simplices that are adjacent to σ via either upper or lower adjacency, namely,

(1) $$N^{\left(q\right)}\left(\sigma \right)=\left\{\tau \in \Delta_{\left(q\right)}|\sigma ⌣\tau \;\text{or}\;\sigma ⌣\tau \right\}.$$

Note that if σ is a 0‐simplex (i.e., a vertex), the neighborhood $N^{\left(0\right)}\left(\sigma \right)$ consists of all 0‐simplices that are upper adjacent to σ. This naturally generalizes the notion of a vertex neighborhood in the conventional graph model.

This topological framework encodes adjacency relationships among simplices in a simplicial complex, which we will leverage to build multiscale topological representations of drug molecules and cell lines (see Section 4.3). Specifically, we apply this framework to simplicial complexes generated from similarity‐based k‐nearest‐neighbor graphs and investigate how varying k (the number of neighbors in the graph) produces a multiscale filtration that captures both local and global structural patterns.

#### 4.2.2 Filtration‐Based Multiscale Representation

To capture the multiscale structural properties of drug molecules and cell lines, we construct simplicial complexes using a filtration process based on similarity‐driven kNN graphs. For drugs, molecular similarities are computed from structural fingerprints using cosine similarity between fingerprint vectors. For cell lines, similarities are derived similarly from gene expression profiles using cosine similarity.

Given a set of entities $V=\{v_{1},v_{2},\text{…},v_{n}\}$ and a similarity function $s:V\times V\to R_{\geq 0}$, we construct a kNN graph $G_{k}=\left(V,E_{k}\right)$ where each node is connected to its neighbors the most similar k nodes. From this graph, we construct the simplicial complex $\Delta_{k}$ as the clique complex generated by the edge connections in $G_{k}$, i.e., $\sigma \in \Delta_{k}$ if and only if |σ|=1 or every pair of distinct vertices in σ forms an edge in E. In other words, the clique complex $\Delta_{k}$ encodes every fully connected subgraph of $G_{k}$ as a simplex. Furthermore, by considering an increasing sequence of values $k_{1}<k_{2}<\cdots <k_{m}$, we obtain a filtration

$$\Delta_{k_{1}}\subseteq \Delta_{k_{2}}\subseteq \cdots \subseteq \Delta_{k_{m}},$$

which yields a multiscale representation that encodes higher‐order interactions between entities, capturing varying levels of similarity as measured by different k values.

Given a fixed number of neighbors k, we define the q‐*th adjacency matrix* $A_{k,q}$, whose rows and columns correspond to the q‐simplices of $\Delta_{k}$. For $\sigma_{i},\sigma_{j}\in {\left(\Delta_{k}\right)}_{\left(q\right)}$, the entry $A_{k,q}\left(i,j\right)$ is assigned according to either *upper* or *lower* adjacency, depending on the value of q.

For q=0 (vertices), adjacency is defined via *upper adjacency*:

(2) $$A_{k,0}\left(i,j\right)=\left\{\begin{array}{cc} 1, & \text{if}\;\sigma_{i}^{0}⌣\sigma_{j}^{0}\;\text{and}\;\sigma_{i}^{0}\neq \sigma_{j}^{0},\\ 0, & \text{otherwise}. \end{array}\right.$$

For q≥1 (q‐simplices with q≥1), adjacency is defined via *lower adjacency*:

(3) $$A_{k,q}\left(i,j\right)=\left\{\begin{array}{cc} 1, & \text{if}\;\sigma_{i}^{q}⌣\sigma_{j}^{q}\;\text{and}\;\sigma_{i}^{q}\neq \sigma_{j}^{q},\\ 0, & \text{otherwise}, \end{array}\right.$$

where $\sigma_{i}^{q},\sigma_{j}^{q}\in {\left(\Delta_{k}\right)}_{\left(q\right)}$ denote the i‐th and j‐th q‐simplices, respectively.

In this study, we apply the framework to both drug molecules and cell lines. To capture multiscale topological information, we construct simplicial complexes at two scales, using k=5 and k=10 nearest neighbors. These dual‐scale complexes serve as the basis for subsequent learning tasks.

### 4.3 Simplicial Attention Learning for Multiscale Representation

To effectively learn expressive features from multiscale simplicial complexes, we design a neural architecture that simultaneously captures both local topological structure and global high‐order dependencies among q‐simplices (q=0,1,2). For each dimension q, we employ a dual‐branch mechanism: a simplicial attention network that propagates information along the simplicial adjacency relations (upper or lower), and a Transformer‐style self‐attention module that enables dense, long‐range interactions between all q‐implices. The outputs from both branches are fused and transformed by a shared MLP. Finally, all higher‐order simplex features (i.e., from 1‐ and 2‐simplices) are aggregated back to 0‐simplices through topological incidence relations, yielding enriched vertex‐level representations that integrate multiscale and higher‐order structural information.

#### 4.3.1 Simplicial Attention over Order‐Wise Simplices

In order to capture high‐order topological dependencies beyond pairwise relations, we extend the Graph Attention Network (GAT) from graphs to simplicial complexes. Traditional GATs operate on node‐level (0‐simplex) graphs, where attention is computed over immediate graph neighbors. In contrast, our approach applies GAT independently to q‐implices (q∈{0,1,2}), allowing each q‐simplex to interact with its topologically defined neighbors–either via upper or lower adjacency–within the simplicial complex. This formulation enables attention‐based reasoning on higher‐dimensional structures, thus enriching the representational capacity beyond graph‐level modeling.

Given a simplicial complex $\Delta =\Delta_{k}$ for some number of neighbors k, with $|\Delta_{\left(q\right)}|=N_{q}$, let $X^{\left(q\right)}\in R^{d\times N_{q}}$ denote the corresponding feature matrix. For each attention head h∈{1,⋯,H}, the attention coefficient between the i‐th and j‐th q‐simplices is computed as:

(4) $$\alpha_{ij}^{\left(q,h\right)}=\frac{\exp\left(\mathrm{LeakyReLU}\left(a_{h}^{⊤}\left[W_{h}^{\left(q\right)}x_{i}^{\left(q\right)}\;∥\;W_{h}^{\left(q\right)}x_{j}^{\left(q\right)}\right]\right)\right)}{\sum_{\sigma_{j^{\prime }}\in N^{\left(q\right)}\left(\sigma_{i}\right)}\exp\left(\mathrm{LeakyReLU}\left(a_{h}^{⊤}\left[W_{h}^{\left(q\right)}x_{i}^{\left(q\right)}\;∥\;W_{h}^{\left(q\right)}x_{j^{\prime }}^{\left(q\right)}\right]\right)\right)},$$

where $x_{i}^{\left(q\right)}$ is the feature vector of the i‐th q‐simplex, $W_{h}^{\left(q\right)}\in R^{d^{\prime }\times d}$ is the learnable weight matrix, and $a_{h}\in R^{2d^{\prime }}$ is the attention vector for head h. As defined in (1), the neighborhood $N^{\left(q\right)}\left(\sigma_{i}\right)$ is the set of all q‐simplices adjacent to $\sigma_{i}$ via either upper or lower adjacency.

Each head computes the output for simplex $\sigma_{i}$ as:

(5) $$z_{i}^{\left(q,h\right)}=\sum_{\sigma_{j}\in N^{\left(q\right)}\left(\sigma_{i}\right)}\alpha_{ij}^{\left(q,h\right)}W_{h}^{\left(q\right)}x_{j}^{\left(q\right)}.$$

The outputs of all heads are concatenated and linearly projected back to dimension d:

(6) $${\tilde{x}}_{i}^{\left(q\right)}=W_{\text{out}}^{\left(q\right)}\left[z_{i}^{\left(q,1\right)}\;∥\;z_{i}^{\left(q,2\right)}\;∥\cdots ∥\;z_{i}^{\left(q,H\right)}\right],$$

where $W_{\text{out}}^{\left(q\right)}\in R^{d\times (H\cdot d^{\prime })}$ is a learnable output projection matrix. The updated features across all $N_{q}$ q‐simplices form the matrix ${\tilde{X}}^{\left(q\right)}\in R^{d\times N_{q}}$.

Furthermore, to enhance expressivity, ${\tilde{X}}^{\left(q\right)}$ is passed through an MLP with residual connection and layer normalization:

(7) $$X_{\text{local}}^{\left(q\right)}={\mathrm{MLP}}^{\left(q\right)}\left({\tilde{X}}^{\left(q\right)}\right).$$

This pipeline equips each q‐simplex with context‐aware representations by attending over its topologically relevant neighbors, generalizing the concept of attention from graph nodes to higher‐order structures in a simplicial complex.

#### 4.3.2 Simplicial Self‐Attention for Global High‐Order Interactions

While the simplicial GAT captures localized interactions based on topological adjacency (i.e., upper or lower adjacency within the simplicial complex), many important structural patterns may exist beyond immediate neighbors. To address this, we introduce a Transformer‐style self‐attention mechanism on simplicial complexes. Specifically, for each q‐simplex order (q=0,1,2), we compute dense self‐attention across all q‐simplices, enabling the model to capture global high‐order dependencies–beyond topological adjacency–within the same dimensional space.

Specifically, let $X^{\left(q\right)}\in R^{d\times N_{q}}$ be the feature matrix for all q‐simplices. For each head h=1,⋯,H, we compute:

(8) $$\begin{array}{cc} Q_{h}^{\left(q\right)} & =W_{Q,h}^{\left(q\right)}X^{\left(q\right)},\\ K_{h}^{\left(q\right)} & =W_{K,h}^{\left(q\right)}X^{\left(q\right)},\\ V_{h}^{\left(q\right)} & =W_{V,h}^{\left(q\right)}X^{\left(q\right)},\\ Z_{h}^{\left(q\right)} & =\mathrm{Softmax}\left(\frac{{\left(Q_{h}^{\left(q\right)}\right)}^{⊤}K_{h}^{\left(q\right)}}{\sqrt{d}}\right){\left(V_{h}^{\left(q\right)}\right)}^{⊤}. \end{array}$$

Each head computes self‐attention among all q‐simplices, regardless of their topological connection. The outputs from all heads are concatenated and projected:

(9) $$Z^{\left(q\right)}=W_{\text{out}}^{\left(q\right)}\left[Z_{1}^{\left(q\right)}|Z_{2}^{\left(q\right)}|\cdots |Z_{H}^{\left(q\right)}\right],$$

followed by a shared MLP transformation with residual connections and layer normalization:

(10) $$X_{\text{attn}}^{\left(q\right)}={\mathrm{MLP}}_{\text{attn}}^{\left(q\right)}\left(Z^{\left(q\right)}\right).$$

Unlike the localized simplicial GAT, this self‐attention branch enables long‐range, order‐wise message passing among all q‐simplices in the complex. It provides complementary non‐local information that is not constrained by adjacency, thus enhancing the model's capacity to capture global topological and functional dependencies in high‐dimensional chemical or biological structures.

#### 4.3.3 Fusion and Cross‐Order Aggregation

We fuse the two information sources via element‐wise addition:

(11) $$H^{\left(q\right)}=X_{\text{local}}^{\left(q\right)}+X_{\text{attn}}^{\left(q\right)}.$$

Next, to integrate higher‐order topology into 0‐simplex representations, we average information from all 1‐ and 2‐simplices that contain a given 0‐simplex. For each vertex $v_{i}$, let $I^{\left(1\right)}\left(v_{i}\right)$ and $I^{\left(2\right)}\left(v_{i}\right)$ denote its incident 1‐ and 2‐simplices; that is, $\sigma^{q}\in I^{\left(q\right)}\left(v_{i}\right)$ if and only if $\sigma^{q}\in \Delta_{\left(q\right)}$ and $v_{i}\in \sigma^{q}$ (q=1,2).

Then the final representation is:

(12) $${\hat{h}}_{i}^{\left(0\right)}=H_{i}^{\left(0\right)}+\frac{1}{|I^{\left(1\right)}\left(v_{i}\right)|}\sum_{\sigma_{j}^{1}\in I^{\left(1\right)}\left(v_{i}\right)}H_{j}^{\left(1\right)}+\frac{1}{|I^{\left(2\right)}\left(v_{i}\right)|}\sum_{\sigma_{j}^{2}\in I^{\left(2\right)}\left(v_{i}\right)}H_{j}^{\left(2\right)}.$$

This yields a final enriched representation for each 0‐simplex that integrates local and global, low‐order and high‐order topological information.

#### 4.3.4 Initial Representation from Simplex Message

To enable learning on the simplicial complexes, we initialize features for all q‐simplices (q=0,1,2) in both the drug and cell line complexes. For 0‐simplices, which represent drug molecules or cell lines, the initial features are directly taken from domain‐specific descriptors: molecular fingerprints for drugs, and normalized gene expression profiles for cell lines.

For 1‐implices and 2‐simplices, which encode pairwise and triplet relations respectively, we derive features based on pairwise distance values. Specifically, for each 1‐simplex connecting i‐th and j‐th entities, we compute a distance value $d_{ij}$ and apply radial basis function (RBF) encoding over a fixed set of centers $c_{l}$:

(13) $$x_{ij}^{\left(1\right)}=e^{-{\left(d_{ij}-c_{l}\right)}^{2}},\;c_{l}\in \left[0,10\right],\;\text{step}=0.1.$$

For each 2‐simplex formed by entities i, j, and k, we extract the three pairwise distances $d_{ij},d_{ik},d_{jk}$ and concatenate their RBF encodings:

(14) $$x_{ijk}^{\left(2\right)}=\left[e^{-{\left(d_{ij}-c_{l}\right)}^{2}}|e^{-{\left(d_{ik}-c_{l}\right)}^{2}}|e^{-{\left(d_{jk}-c_{l}\right)}^{2}}\right],\;c_{l}\in \left[0,4\right],\;\text{step}=0.1.$$

This initialization process yields continuous, fixed‐length feature vectors for all simplices, encoding both domain knowledge (fingerprints or expression) and topological geometry (distance‐based RBFs), providing a consistent foundation for downstream simplicial neural encoders.

### 4.4 Prediction and Optimization

After extracting the simplicial features, we obtain enriched representations for both drugs and cell lines from their respective multiscale simplicial complexes. Let ${\hat{h}}_{i}^{\left(0\right)}\left(i=1,2,\text{…}n_{D}\right)$ denote the 0‐simplex features of a drug molecule, and ${\hat{h}}_{j}^{\left(0\right)}\left(j=1,2,\text{…}n_{C}\right)$ for a cell line. We apply mean pooling to obtain fixed‐size vectors:

(15) $$d=\frac{1}{n_{D}}\sum_{i=1}^{n_{D}}{\hat{h}}_{i}^{\left(0\right)},\;c=\frac{1}{n_{C}}\sum_{j=1}^{n_{C}}{\hat{h}}_{j}^{\left(0\right)},$$

where $d,c\in R^{d}$ are the final feature vectors for the drug and cell line, respectively.

We then concatenate the two vectors and feed them into a fully connected feedforward network to predict drug response:

(16) $$\hat{y}=\mathrm{MLP}\left(z\right),\;\text{where}\;z=\left[d\;|\;c\right]\in R^{2d}.$$

The MLP consists of three linear layers with nonlinear activation (e.g., ReLU) and dropout applied between layers. The output $\hat{y}\in R$ is a scalar indicating the predicted response of a given drug‐cell line pair.

To train the model, we use the mean squared error (MSE) loss between predicted responses $\hat{y}$ and the ground truth responses y:

(17) $$L_{\mathrm{MSE}}=\frac{1}{N}\sum_{i=1}^{N}{\left(y_{i}-{\hat{y}}_{i}\right)}^{2},$$

where N is the number of training samples.

This framework allows the model to jointly leverage the multiscale topological structures of both drugs and cell lines for accurate prediction of cancer drug response.

## Conflicts of Interest

The author declare no conflicts of interest.

## Supporting information


**Supporting File**: advs75816‐sup‐0001‐SuppMat.pdf.

## Data Availability

Data sharing not applicable to this article as no datasets were generated or analysed during the current study.
