# Supplementary material for: Topology‐Aware Deep Learning on Higher‐Order Structures for Drug Response Prediction
Source: Adv Sci (Weinh). 2026 May 27;13(46):e75816. doi: 10.1002/advs.75816 (PMC13335842; doi:10.1002/advs.75816)
Supplement: Supplementary file 1 [file ADVS-13-e75816-s001.pdf]

# Supplementary Material:

## Topology-Aware Deep Learning on Higher-Order Structures for Drug Response Prediction

Cong Shen[1*], Guancen Lin[1], Chuan-Shen Hu[2], Yong Wang[1,3,4,5*]

[1*]State Key Laboratory of Mathematical Sciences, Academy of Mathematics and Systems Science, Chinese Academy of Sciences, Beijing, 100190, Beijing, China.
[2]Department of Applied Mathematics, National University of Kaohsiung, Kaohsiung, 81148, Kaohsiung, Taiwan.
[3]School of Mathematics, University of Chinese Academy of Sciences, Chinese Academy of Sciences, Beijing, 100049, Beijing, China.
[4]Center for Excellence in Animal Evolution and Genetics, Chinese Academy of Sciences, Kunming, 650223, Yunnan, China.
[5]Key Laboratory of Systems Biology, Hangzhou Institute for Advanced Study, University of Chinese Academy of Sciences, Chinese Academy of Sciences, Hangzhou, 330106, Zhejiang, China.

*Corresponding author(s). E-mail(s): cshen@amss.ac.cn; ywang@amss.ac.cn;
Contributing authors: linguancen@amss.ac.cn; chuanshenhu1@nuk.edu.tw;

**Table S1** Performance comparison of different methods on six datasets under three evaluation metrics. For PCC, higher values indicate better performance, while for RMSE and MAE, lower values are preferred. The best results are highlighted in bold. Values in parentheses denote the standard deviation over five runs.

| Metric | Methods | TGSA | GDSC1 | GDSC2 | CCLE | CTRP1 | CTRP2 |
|---|---|---|---|---|---|---|---|
| PCC | MultiDRP | $0.9354_{(0.001)}$ | $0.9209_{(0.003)}$ | $0.9349_{(0.003)}$ | $0.8760_{(0.001)}$ | $0.7962_{(0.002)}$ | $0.8801_{(0.002)}$ |
| | MSDRP | $0.9335_{(0.006)}$ | $0.9225_{(0.001)}$ | $0.9369_{(0.001)}$ | $0.8722_{(0.002)}$ | $0.8194_{(0.001)}$ | $0.8954_{(0.003)}$ |
| | PANCDR | $0.8966_{(0.000)}$ | $0.9076_{(0.001)}$ | $0.9352_{(0.002)}$ | $0.8637_{(0.001)}$ | $0.7083_{(0.001)}$ | $0.8977_{(0.002)}$ |
| | GraphCDR | $0.8957_{(0.002)}$ | $0.8844_{(0.004)}$ | $0.9055_{(0.002)}$ | $0.8234_{(0.004)}$ | $0.7257_{(0.000)}$ | $0.7556_{(0.004)}$ |
| | SubCDR | $0.9205_{(0.001)}$ | $0.9264_{(0.000)}$ | $0.9327_{(0.003)}$ | $0.8611_{(0.002)}$ | $0.7735_{(0.001)}$ | $0.8715_{(0.001)}$ |
| | A-DGN | $0.9310_{(0.001)}$ | $0.9129_{(0.004)}$ | $0.9318_{(0.001)}$ | $0.8687_{(0.002)}$ | $0.8026_{(0.002)}$ | $0.8771_{(0.001)}$ |
| | ARMA | $0.9295_{(0.003)}$ | $0.9258_{(0.001)}$ | $0.9372_{(0.002)}$ | $0.8761_{(0.001)}$ | $0.7983_{(0.002)}$ | $0.8980_{(0.003)}$ |
| | EGC | $0.9295_{(0.004)}$ | $0.9266_{(0.002)}$ | $0.9345_{(0.004)}$ | $0.8743_{(0.003)}$ | $0.8028_{(0.001)}$ | $0.8956_{(0.002)}$ |
| | GraphGPS | $0.9353_{(0.005)}$ | $0.9263_{(0.003)}$ | $0.9402_{(0.002)}$ | $0.8761_{(0.003)}$ | $0.8159_{(0.005)}$ | $0.9025_{(0.002)}$ |
| | SSGC | $0.9236_{(0.000)}$ | $0.9106_{(0.003)}$ | $0.9294_{(0.000)}$ | $0.8694_{(0.000)}$ | $0.8027_{(0.002)}$ | $0.8798_{(0.004)}$ |
| | HGNN | $0.9063_{(0.002)}$ | $0.8910_{(0.004)}$ | $0.9041_{(0.002)}$ | $0.8517_{(0.001)}$ | $0.6991_{(0.001)}$ | $0.8609_{(0.004)}$ |
| | HGNN$^{+}$ | $0.8626_{(0.003)}$ | $0.8597_{(0.002)}$ | $0.8685_{(0.001)}$ | $0.8150_{(0.001)}$ | $0.5033_{(0.004)}$ | $0.8175_{(0.002)}$ |
| | BScNets | $0.9069_{(0.003)}$ | $0.9007_{(0.000)}$ | $0.9119_{(0.001)}$ | $0.8730_{(0.003)}$ | $0.7327_{(0.003)}$ | $0.8504_{(0.001)}$ |
| | HiGCN | $0.9009_{(0.001)}$ | $0.9010_{(0.001)}$ | $0.9155_{(0.004)}$ | $0.8717_{(0.005)}$ | $0.7126_{(0.002)}$ | $0.8387_{(0.003)}$ |
| | MolGT | $0.8708_{(0.005)}$ | $0.8873_{(0.000)}$ | $0.8974_{(0.002)}$ | $0.8308_{(0.004)}$ | $0.6678_{(0.004)}$ | $0.8108_{(0.001)}$ |
| | TopDr | $\mathbf{0.9418}_{(0.002)}$ | $\mathbf{0.9318}_{(0.001)}$ | $\mathbf{0.9423}_{(0.000)}$ | $\mathbf{0.8791}_{(0.001)}$ | $\mathbf{0.8319}_{(0.001)}$ | $\mathbf{0.9026}_{(0.003)}$ |
| RMSE | MultiDRP | $1.0013_{(0.009)}$ | $1.0441_{(0.012)}$ | $1.0153_{(0.030)}$ | $0.7539_{(0.023)}$ | $1.0354_{(0.012)}$ | $1.2539_{(0.022)}$ |
| | MSDRP | $1.0005_{(0.012)}$ | $1.0274_{(0.031)}$ | $0.9956_{(0.029)}$ | $0.7719_{(0.018)}$ | $0.9900_{(0.024)}$ | $1.1764_{(0.015)}$ |
| | PANCDR | $1.3047_{(0.021)}$ | $1.1240_{(0.030)}$ | $1.0243_{(0.014)}$ | $0.8106_{(0.015)}$ | $1.2346_{(0.015)}$ | $1.1582_{(0.013)}$ |
| | GraphCDR | $1.4247_{(0.010)}$ | $1.2358_{(0.022)}$ | $1.2572_{(0.012)}$ | $0.9679_{(0.022)}$ | $1.1950_{(0.016)}$ | $1.7349_{(0.028)}$ |
| | SubCDR | $1.2384_{(0.015)}$ | $1.0200_{(0.018)}$ | $1.0549_{(0.012)}$ | $\mathbf{0.6636}_{(0.009)}$ | $1.2058_{(0.014)}$ | $1.6836_{(0.022)}$ |
| | A-DGN | $1.0331_{(0.018)}$ | $1.0783_{(0.012)}$ | $1.0411_{(0.030)}$ | $0.8021_{(0.017)}$ | $1.0216_{(0.014)}$ | $1.2617_{(0.007)}$ |
| | ARMA | $1.0456_{(0.015)}$ | $0.9967_{(0.020)}$ | $1.0015_{(0.028)}$ | $0.7812_{(0.014)}$ | $1.0378_{(0.017)}$ | $1.1592_{(0.016)}$ |
| | EGC | $1.0456_{(0.019)}$ | $1.0078_{(0.009)}$ | $1.0171_{(0.016)}$ | $0.7867_{(0.023)}$ | $1.0217_{(0.024)}$ | $1.1729_{(0.021)}$ |
| | GraphGPS | $0.9995_{(0.006)}$ | $0.9951_{(0.014)}$ | $0.9770_{(0.005)}$ | $0.7801_{(0.016)}$ | $0.9895_{(0.020)}$ | $\mathbf{1.1316}_{(0.027)}$ |
| | SSGC | $1.0837_{(0.008)}$ | $1.0922_{(0.017)}$ | $1.0586_{(0.007)}$ | $0.8005_{(0.003)}$ | $1.0185_{(0.010)}$ | $1.2530_{(0.016)}$ |
| | HGNN | $1.2029_{(0.022)}$ | $1.2086_{(0.026)}$ | $1.2137_{(0.016)}$ | $0.8264_{(0.012)}$ | $1.2324_{(0.025)}$ | $1.3449_{(0.010)}$ |
| | HGNN$^{+}$ | $1.4390_{(0.016)}$ | $1.3595_{(0.022)}$ | $1.4099_{(0.021)}$ | $0.9133_{(0.033)}$ | $1.4897_{(0.013)}$ | $1.5218_{(0.011)}$ |
| | BScNets | $1.1939_{(0.011)}$ | $1.1573_{(0.028)}$ | $1.1659_{(0.028)}$ | $0.7683_{(0.018)}$ | $1.1735_{(0.013)}$ | $1.3943_{(0.022)}$ |
| | HiGCN | $1.2811_{(0.014)}$ | $1.1565_{(0.015)}$ | $1.1478_{(0.017)}$ | $0.7752_{(0.009)}$ | $1.2391_{(0.016)}$ | $1.4641_{(0.020)}$ |
| | MolGT | $1.4012_{(0.007)}$ | $1.2501_{(0.011)}$ | $1.2273_{(0.013)}$ | $0.9284_{(0.007)}$ | $1.3266_{(0.021)}$ | $1.5651_{(0.013)}$ |
| | TopDr | $\mathbf{0.9484}_{(0.016)}$ | $\mathbf{0.9762}_{(0.012)}$ | $\mathbf{0.9608}_{(0.009)}$ | $0.7454_{(0.011)}$ | $\mathbf{0.9562}_{(0.013)}$ | $1.1331_{(0.005)}$ |
| MAE | MultiDRP | $0.7346_{(0.004)}$ | $0.7750_{(0.007)}$ | $0.7515_{(0.019)}$ | $0.4688_{(0.006)}$ | $0.7745_{(0.012)}$ | $0.8769_{(0.011)}$ |
| | MSDRP | $0.7409_{(0.015)}$ | $0.7639_{(0.012)}$ | $0.7425_{(0.022)}$ | $0.4735_{(0.012)}$ | $0.7404_{(0.022)}$ | $0.8184_{(0.017)}$ |
| | PANCDR | $0.9802_{(0.022)}$ | $0.8339_{(0.024)}$ | $0.7762_{(0.015)}$ | $0.4934_{(0.022)}$ | $0.9577_{(0.044)}$ | $0.8171_{(0.019)}$ |
| | GraphCDR | $1.1301_{(0.034)}$ | $0.9189_{(0.031)}$ | $0.9406_{(0.013)}$ | $0.6161_{(0.029)}$ | $0.9041_{(0.021)}$ | $1.2216_{(0.025)}$ |
| | SubCDR | $0.8442_{(0.021)}$ | $0.7566_{(0.028)}$ | $0.7780_{(0.017)}$ | $0.4857_{(0.012)}$ | $0.8397_{(0.022)}$ | $0.9221_{(0.023)}$ |
| | A-DGN | $0.7709_{(0.013)}$ | $0.8098_{(0.013)}$ | $0.7820_{(0.014)}$ | $0.4889_{(0.011)}$ | $0.7760_{(0.021)}$ | $0.9091_{(0.013)}$ |
| | ARMA | $0.7836_{(0.009)}$ | $0.7405_{(0.019)}$ | $0.7464_{(0.025)}$ | $0.4771_{(0.007)}$ | $0.7750_{(0.016)}$ | $0.8207_{(0.015)}$ |
| | EGC | $0.7836_{(0.015)}$ | $0.7486_{(0.007)}$ | $0.7586_{(0.009)}$ | $0.4814_{(0.015)}$ | $0.7662_{(0.014)}$ | $0.8314_{(0.018)}$ |
| | GraphGPS | $0.7533_{(0.017)}$ | $0.7383_{(0.012)}$ | $0.7267_{(0.011)}$ | $0.4791_{(0.021)}$ | $0.7427_{(0.021)}$ | $0.8078_{(0.036)}$ |
| | SSGC | $0.8194_{(0.023)}$ | $0.8191_{(0.011)}$ | $0.7973_{(0.015)}$ | $0.4876_{(0.027)}$ | $0.7584_{(0.028)}$ | $0.8914_{(0.022)}$ |
| | HGNN | $0.9063_{(0.041)}$ | $0.9072_{(0.015)}$ | $0.9128_{(0.026)}$ | $0.5013_{(0.031)}$ | $0.9410_{(0.012)}$ | $0.9478_{(0.012)}$ |
| | HGNN$^{+}$ | $1.0921_{(0.031)}$ | $1.0197_{(0.034)}$ | $1.0676_{(0.028)}$ | $0.5426_{(0.038)}$ | $1.1767_{(0.016)}$ | $1.0786_{(0.016)}$ |
| | BScNets | $0.8936_{(0.019)}$ | $0.8614_{(0.012)}$ | $0.8653_{(0.019)}$ | $0.4702_{(0.007)}$ | $0.8877_{(0.013)}$ | $0.9763_{(0.011)}$ |
| | HiGCN | $1.2811_{(0.027)}$ | $0.8622_{(0.014)}$ | $0.8593_{(0.025)}$ | $0.4734_{(0.006)}$ | $0.9643_{(0.017)}$ | $1.0335_{(0.010)}$ |
| | MolGT | $1.0905_{(0.022)}$ | $0.9436_{(0.018)}$ | $0.9412_{(0.031)}$ | $0.5136_{(0.013)}$ | $1.0992_{(0.015)}$ | $1.0161_{(0.014)}$ |
| | TopDr | $\mathbf{0.7114}_{(0.012)}$ | $\mathbf{0.7187}_{(0.011)}$ | $\mathbf{0.7109}_{(0.014)}$ | $\mathbf{0.4671}_{(0.009)}$ | $\mathbf{0.7058}_{(0.008)}$ | $\mathbf{0.7900}_{(0.016)}$ |

**Table S2** Statistical significance analysis (p-values) of different models across multiple datasets. Lower p-values indicate stronger statistical significance.

| Metric | Methods | TGSA | GDSC1 | GDSC2 | CCLE | CTRP1 | CTRP2 |
|---|---|---|---|---|---|---|---|
| PCC | MultiDRP | 3.21E-02 | 8.36E-06 | 1.54E-03 | 2.87E-02 | 7.42E-05 | 4.13E-04 |
| | MSDRP | 9.12E-03 | 2.66E-02 | 5.18E-07 | 1.33E-04 | 3.75E-02 | 6.41E-06 |
| | PANCDR | 4.62E-04 | 7.25E-03 | 2.98E-02 | 6.87E-06 | 1.54E-02 | 8.12E-05 |
| | GraphCDR | 1.82E-02 | 9.74E-06 | 3.64E-02 | 7.11E-04 | 2.05E-03 | 5.77E-03 |
| | SubCDR | 6.55E-03 | 1.24E-04 | 8.17E-06 | 3.92E-02 | 7.65E-03 | 2.88E-04 |
| | A-DGN | 4.11E-05 | 2.44E-02 | 9.63E-04 | 1.76E-06 | 2.94E-02 | 6.83E-07 |
| | ARMA | 7.83E-03 | 5.12E-05 | 3.11E-02 | 8.63E-04 | 1.07E-02 | 2.55E-03 |
| | EGC | 2.56E-02 | 4.91E-04 | 6.77E-06 | 3.85E-02 | 9.41E-03 | 1.62E-04 |
| | GraphGPS | 8.75E-05 | 3.52E-02 | 1.43E-03 | 5.18E-06 | 2.64E-02 | 7.93E-05 |
| | SSGC | 1.36E-02 | 6.88E-04 | 2.75E-02 | 9.01E-06 | 3.12E-03 | 4.44E-02 |
| | HGNN | 2.91E-02 | 8.43E-05 | 7.11E-04 | 1.95E-02 | 5.33E-03 | 9.77E-06 |
| | $HGNN^{+}$ | 7.62E-04 | 3.14E-02 | 1.25E-05 | 6.82E-03 | 2.48E-02 | 8.94E-07 |
| | BScNets | 1.99E-02 | 6.17E-06 | 3.87E-02 | 2.56E-03 | 4.83E-04 | 7.61E-03 |
| | HiGCN | 5.11E-03 | 1.88E-04 | 7.25E-06 | 2.74E-02 | 1.33E-02 | 4.17E-05 |
| | MolGT | 3.42E-02 | 7.66E-05 | 2.94E-03 | 1.22E-02 | 6.54E-04 | 8.73E-06 |
| RMSE | MultiDRP | 1.73E-02 | 2.14E-05 | 8.33E-04 | 3.05E-02 | 6.77E-06 | 1.92E-03 |
| | MSDRP | 4.82E-03 | 3.22E-02 | 9.11E-06 | 7.84E-04 | 2.63E-02 | 5.77E-05 |
| | PANCDR | 2.94E-04 | 6.31E-03 | 3.11E-02 | 4.26E-05 | 8.72E-03 | 1.33E-04 |
| | GraphCDR | 9.88E-03 | 1.74E-06 | 2.81E-02 | 6.77E-04 | 2.17E-03 | 4.01E-05 |
| | SubCDR | 5.63E-03 | 2.18E-04 | 6.55E-06 | 3.61E-02 | 8.14E-03 | 3.09E-04 |
| | A-DGN | 3.17E-05 | 2.58E-02 | 7.93E-04 | 1.22E-06 | 2.86E-02 | 4.63E-07 |
| | ARMA | 8.11E-03 | 4.63E-05 | 2.77E-02 | 7.45E-04 | 9.55E-03 | 3.66E-03 |
| | EGC | 2.75E-02 | 3.14E-04 | 8.12E-06 | 3.55E-02 | 6.84E-03 | 2.47E-04 |
| | GraphGPS | 6.88E-05 | 3.14E-02 | 1.77E-03 | 4.52E-06 | 2.45E-02 | 9.33E-05 |
| | SSGC | 1.44E-02 | 7.91E-04 | 2.48E-02 | 7.88E-06 | 3.22E-03 | 4.21E-02 |
| | HGNN | 2.66E-02 | 9.22E-05 | 6.44E-04 | 2.05E-02 | 5.92E-03 | 8.33E-06 |
| | $HGNN^{+}$ | 5.92E-04 | 3.27E-02 | 1.88E-05 | 5.11E-03 | 2.14E-02 | 7.12E-07 |
| | BScNets | 1.85E-02 | 5.33E-06 | 3.22E-02 | 2.66E-03 | 5.11E-04 | 8.55E-03 |
| | HiGCN | 6.44E-03 | 2.47E-04 | 8.63E-06 | 2.41E-02 | 1.18E-02 | 5.77E-05 |
| | MolGT | 3.14E-02 | 8.12E-05 | 3.88E-03 | 1.01E-02 | 7.92E-04 | 6.11E-06 |
| MAE | MultiDRP | 1.82E-02 | 3.14E-05 | 9.11E-04 | 2.88E-02 | 5.44E-06 | 1.72E-03 |
| | MSDRP | 4.66E-03 | 3.05E-02 | 7.88E-06 | 6.77E-04 | 2.55E-02 | 4.92E-05 |
| | PANCDR | 3.11E-04 | 6.02E-03 | 2.95E-02 | 5.12E-05 | 9.33E-03 | 1.11E-04 |
| | GraphCDR | 9.21E-03 | 1.52E-06 | 2.66E-02 | 7.14E-04 | 2.44E-03 | 3.88E-05 |
| | SubCDR | 5.01E-03 | 2.05E-04 | 6.14E-06 | 3.44E-02 | 7.77E-03 | 3.66E-04 |
| | A-DGN | 2.88E-05 | 2.36E-02 | 8.22E-04 | 1.09E-06 | 2.77E-02 | 5.02E-07 |
| | ARMA | 7.74E-03 | 4.91E-05 | 2.66E-02 | 8.33E-04 | 8.44E-03 | 3.55E-03 |
| | EGC | 2.61E-02 | 3.77E-04 | 7.77E-06 | 3.22E-02 | 7.11E-03 | 2.18E-04 |
| | GraphGPS | 7.92E-05 | 3.02E-02 | 1.55E-03 | 5.01E-06 | 2.22E-02 | 8.66E-05 |
| | SSGC | 1.28E-02 | 6.55E-04 | 2.33E-02 | 8.44E-06 | 3.44E-03 | 4.09E-02 |
| | HGNN | 2.52E-02 | 8.33E-05 | 6.11E-04 | 2.11E-02 | 5.55E-03 | 9.11E-06 |
| | $HGNN^{+}$ | 6.11E-04 | 3.11E-02 | 1.55E-05 | 5.44E-03 | 2.05E-02 | 6.88E-07 |
| | BScNets | 1.72E-02 | 4.91E-06 | 3.05E-02 | 2.77E-03 | 5.44E-04 | 7.99E-03 |
| | HiGCN | 6.11E-03 | 2.22E-04 | 8.11E-06 | 2.33E-02 | 1.25E-02 | 6.44E-05 |
| | MolGT | 3.02E-02 | 7.77E-05 | 3.55E-03 | 1.11E-02 | 7.11E-04 | 5.88E-06 |

**Table S3** Pathway enrichment metrics of top-ranked 1-simplex drug pairs across four gene set libraries

| Top N | Groups | Drug list | Enrichment significance | | | |
|---|---|---|---|---|---|---|
| | | | **KEGG** | **GP-BP** | **Reactome** | **WikiPathways** |
| Top 1 | Group A | PLX-4720, Selumetinib | 5.86 | 4.78 | 5.95 | 4.27 |
| | Group B | Olaparib, Niraparib | 4.30 | 3.16 | 2.78 | 3.45 |
| Top 2 | Group A | Irinotecan, YK-4-279 | 6.21 | 3.67 | 2.42 | 3.86 |
| | Group B | Olaparib, Niraparib | 5.28 | 3.69 | 2.86 | 4.06 |
| Top 3 | Group A | Dasatinib, Selumetinib | 3.07 | 3.25 | 5.12 | 3.75 |
| | Group B | Venetoclax, Pictilisib | 1.55 | 1.68 | 1.55 | 1.85 |
| Top 4 | Group A | Gefitinib, Cediranib | 6.34 | 5.15 | 4.36 | 4.72 |
| | Group B | Niraparib, Temozolomide | 1.59 | 1.71 | 1.40 | 1.75 |
| Top 5 | Group A | Osimertinib, Nilotinib | 1.53 | 0.90 | 1.15 | 1.78 |
| | Group B | Daporinad, Entinostat | 1.47 | 1.56 | 1.21 | 1.88 |
| | | | **Overlapping pathways** | | | |
| | | | **KEGG** | **GP-BP** | **Reactome** | **WikiPathways** |
| Top 1 | Group A<br>Group B | PLX-4720, Selumetinib<br>Olaparib, Niraparib | 15 | 53 | 31 | 42 |
| Top 2 | Group A<br>Group B | Irinotecan, YK-4-279<br>Olaparib, Niraparib | 10 | 41 | 26 | 32 |
| Top 3 | Group A<br>Group B | Dasatinib, Selumetinib<br>Venetoclax, Pictilisib | 49 | 93 | 32 | 75 |
| Top 4 | Group A<br>Group B | Gefitinib, Cediranib<br>Niraparib, Temozolomide | 7 | 30 | 21 | 19 |
| Top 5 | Group A<br>Group B | Osimertinib, Nilotinib<br>Daporinad, Entinostat | 5 | 17 | 20 | 6 |
| | | | **Jaccard similarity** | | | |
| | | | **KEGG** | **GP-BP** | **Reactome** | **WikiPathways** |
| Top 1 | Group A<br>Group B | PLX-4720, Selumetinib<br>Olaparib, Niraparib | 0.139 | 0.156 | 0.215 | 0.227 |
| Top 2 | Group A<br>Group B | Irinotecan, YK-4-279<br>Olaparib, Niraparib | 0.476 | 0.203 | 0.342 | 0.457 |
| Top 3 | Group A<br>Group B | Dasatinib, Selumetinib<br>Venetoclax, Pictilisib | 0.308 | 0.106 | 0.086 | 0.267 |
| Top 4 | Group A<br>Group B | Gefitinib, Cediranib<br>Niraparib, Temozolomide | 0.109 | 0.096 | 0.174 | 0.157 |
| Top 5 | Group A<br>Group B | Osimertinib, Nilotinib<br>Daporinad, Entinostat | 0.064 | 0.043 | 0.105 | 0.044 |

**Table S4** Random-baseline pathway enrichment statistics for drug simplicial interactions. For each simplex order, random simplex pairs were sampled repeatedly, and the mean enrichment significance, number of overlapping pathways, and Jaccard similarity were computed across four gene set libraries. Enrichment significance is reported as the average $-\log_{10}(\text{adjusted } p)$ over overlapping pathways.

| Simplex order | Metric | KEGG | GO-BP | Reactome | WikiPathways | Average |
|---|---|---|---|---|---|---|
| 1-simplex | Enrichment significance | 0.733 | 1.069 | 1.361 | 1.414 | 1.144 |
| | Overlapping pathways | 3.900 | 6.480 | 6.160 | 4.880 | 5.355 |
| | Jaccard similarity | 0.052 | 0.040 | 0.074 | 0.047 | 0.053 |
| 2-simplex | Enrichment significance | 1.229 | 1.879 | 1.477 | 1.495 | 1.520 |
| | Overlapping pathways | 5.060 | 9.980 | 7.480 | 5.780 | 7.075 |
| | Jaccard similarity | 0.078 | 0.039 | 0.062 | 0.058 | 0.059 |

**Table S5** Representative simplex interactions with low structural similarity but strong functional enrichment. Structural similarity is computed based on molecular fingerprints (Tanimoto similarity). Despite low structural similarity, these interactions exhibit strong pathway enrichment, indicating that TopDr captures functionally meaningful relationships beyond chemical similarity.

| | Case 1 | Case 2 | Case 3 |
|---|---|---|---|
| Rank | 9 | 17 | 16 |
| Simplex Order | 1-simplex | 1-simplex | 2-simplex |
| Group A | (ABT-737, Venetoclax) | (Mirdametinib, PLX-4720) | (Paclitaxel, Epirubicin, Docetaxel) |
| Group B | (Linsitinib, OSI-027) | (Gefitinib, Erlotinib) | (Ribociclib, BI-2536, Palbociclib) |
| Structural Similarity (Tanimoto) | 0.148 | 0.143 | 0.119 |
| Enrichment Significance | 1.824 | 2.132 | 1.633 |
| # Overlapping Pathways | 14.5 | 34.5 | 8.0 |
| Jaccard Similarity | 0.110 | 0.259 | 0.082 |

**Table S6** Pathway enrichment metrics of top-ranked 2-simplex drug pairs across four gene set libraries

| Top N | Groups | Drug list | Enrichment significance | | | |
|---|---|---|---|---|---|---|
| | | | KEGG | GP-BP | Reactome | WikiPathways |
| Top 1 | Group A | Vincristine, Vinorelbine, Vinblastine | 1.74 | 2.26 | 2.36 | 2.05 |
| | Group B | Ribociclib, BI 2536, Palbociclib | 2.38 | 1.64 | 1.59 | 2.38 |
| Top 2 | Group A | Paclitaxel, Epirubicin, Docetaxel | 1.45 | 2.01 | 1.86 | 2.00 |
| | Group B | Vincristine, Irinotecan, Vinorelbine | 2.00 | 1.88 | 1.38 | 2.22 |
| Top 3 | Group A | Lapatinib, Erlotinib, AZD-3759 | 4.78 | 4.48 | 4.90 | 4.90 |
| | Group B | Zoledronic acid, Cyclophosphamide, Fluorouracil | 4.48 | 3.50 | 4.89 | 3.91 |
| Top 4 | Group A | Paclitaxel, Epirubicin, Docetaxel | 1.47 | 1.95 | 1.80 | 2.03 |
| | Group B | Vincristine, Vinorelbine, Cediranib | 1.94 | 1.83 | 1.36 | 2.16 |
| Top 5 | Group A | Olaparib, Talazoparib, Fluorouracil | 3.98 | 3.30 | 4.34 | 3.83 |
| | Group B | ABT-737, Venetoclax, Navitoclax | 2.55 | 1.88 | 1.36 | 2.01 |
| | | | Overlapping pathways | | | |
| | | | KEGG | GP-BP | Reactome | WikiPathways |
| Top 1 | Group A | Vincristine, Vinorelbine, Vinblastine | 37 | 68 | 48 | 50 |
| | Group B | Ribociclib, BI 2536, Palbociclib | | | | |
| Top 2 | Group A | Paclitaxel, Epirubicin, Docetaxel | 69 | 124 | 57 | 91 |
| | Group B | Vincristine, Irinotecan, Vinorelbine | | | | |
| Top 3 | Group A | Lapatinib, Erlotinib, AZD-3759 | 17 | 60 | 31 | 43 |
| | Group B | Zoledronic acid, Cyclophosphamide, Fluorouracil | | | | |
| Top 4 | Group A | Paclitaxel, Epirubicin, Docetaxel | 66 | 124 | 50 | 87 |
| | Group B | Vincristine, Vinorelbine, Cediranib | | | | |
| Top 5 | Group A | Olaparib, Talazoparib, Fluorouracil | 17 | 50 | 24 | 38 |
| | Group B | ABT-737, Venetoclax, Navitoclax | | | | |
| | | | Jaccard similarity | | | |
| | | | KEGG | GP-BP | Reactome | WikiPathways |
| Top 1 | Group A | Vincristine, Vinorelbine, Vinblastine | 0.257 | 0.179 | 0.189 | 0.187 |
| | Group B | Ribociclib, BI 2536, Palbociclib | | | | |
| Top 2 | Group A | Paclitaxel, Epirubicin, Docetaxel | 0.476 | 0.347 | 0.237 | 0.376 |
| | Group B | Vincristine, Irinotecan, Vinorelbine | | | | |
| Top 3 | Group A | Lapatinib, Erlotinib, AZD-3759 | 0.239 | 0.155 | 0.226 | 0.309 |
| | Group B | Zoledronic acid, Cyclophosphamide, Fluorouracil | | | | |
| Top 4 | Group A | Paclitaxel, Epirubicin, Docetaxel | 0.452 | 0.289 | 0.206 | 0.348 |
| | Group B | Vincristine, Vinorelbine, Cediranib | | | | |
| Top 5 | Group A | Olaparib, Talazoparib, Fluorouracil | 0.160 | 0.106 | 0.158 | 0.197 |
| | Group B | ABT-737, Venetoclax, Navitoclax | | | | |

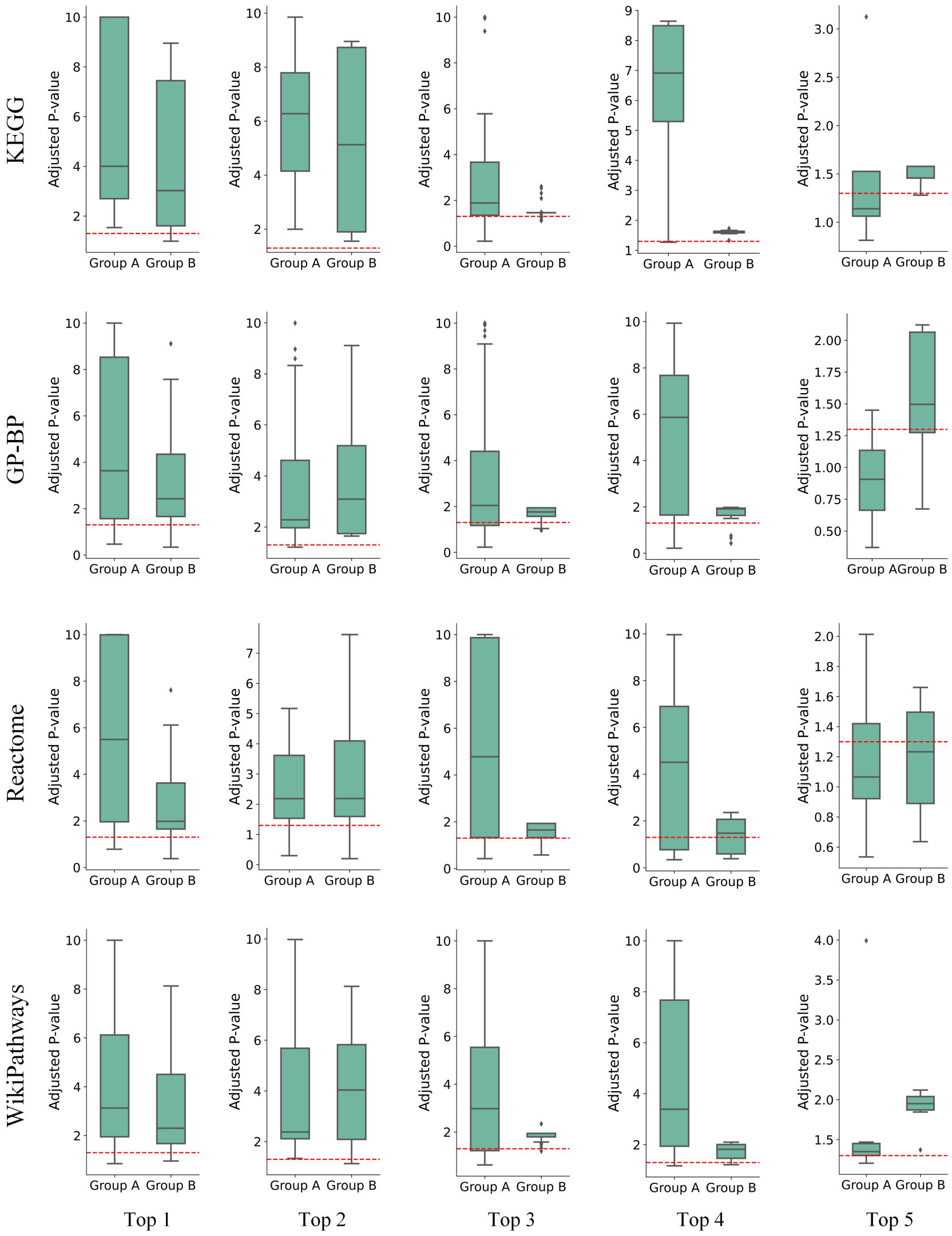


**Fig. S1 Box plots of enrichment significance (adjusted p-values) across top-ranked 1-simplex drug pairs on the TGSA dataset.** Each row corresponds to one of the top five 1-simplex drug pair groups (Top 1–Top 5) ranked by attention scores, and each column represents a distinct pathway database (KEGG, GO Biological Process, Reactome, WikiPathways). Box plots show the distribution of adjusted p-values for Group A and Group B in each drug pair. The red dashed line marks the threshold for statistical significance (adjusted p-value = 1.3, corresponding to p = 0.05).

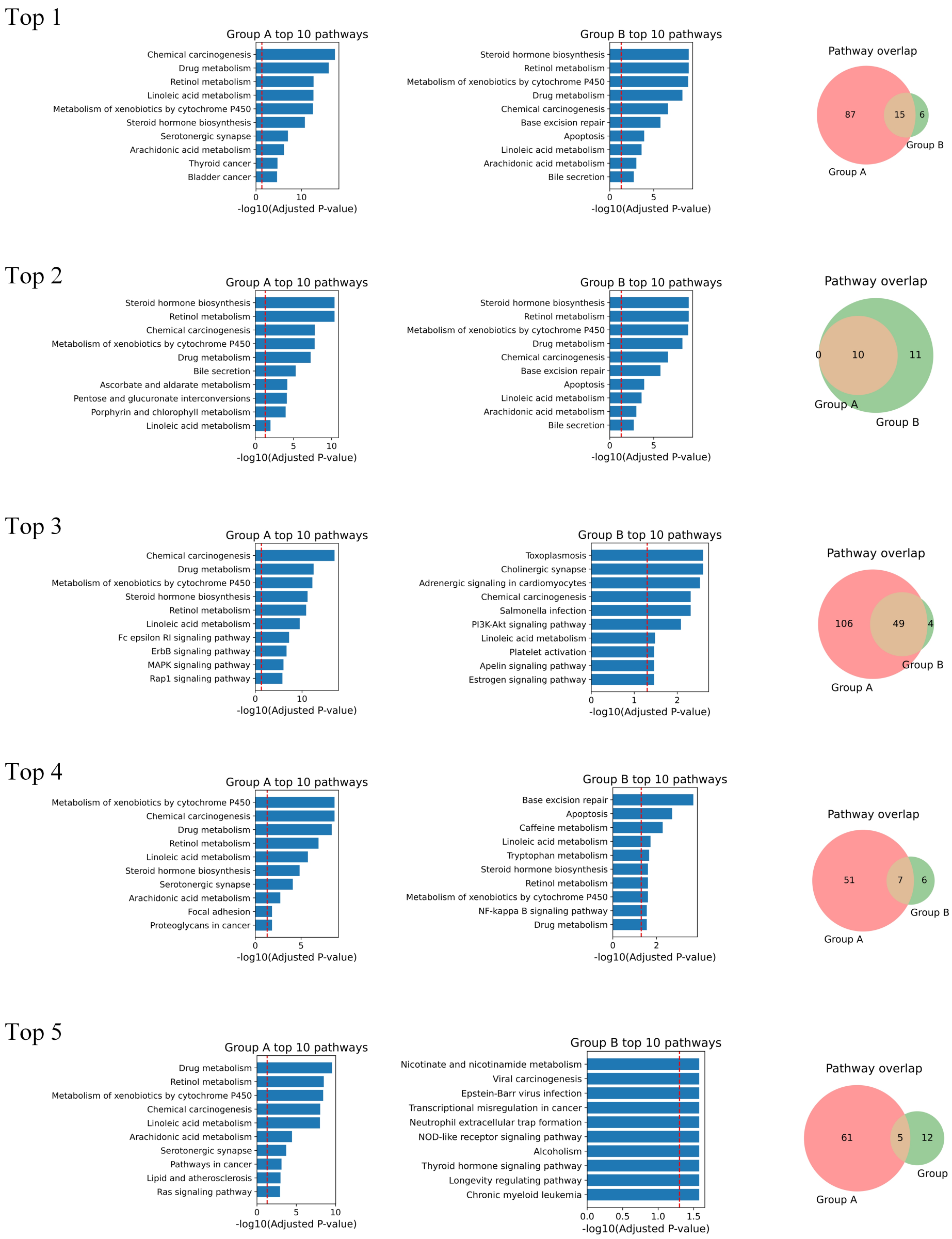


**Fig. S2 Top enriched KEGG pathways of top-ranked 1-simplex drug pairs in the TGSA dataset.** The top five 1-simplex drug pairs with the highest attention scores were selected from the attention matrix learned on the TGSA dataset. For each pair, pathway enrichment analysis was performed on drug targets using the KEGG gene set, and the top 10 enriched pathways (ranked by adjusted p-value) were shown for both Group A and Group B. The red dashed line indicates the statistical significance threshold (adjusted p-value = 1.3, corresponding to $p = 0.05$). The rightmost column presents Venn diagrams illustrating the overlap of enriched pathways between the two groups.

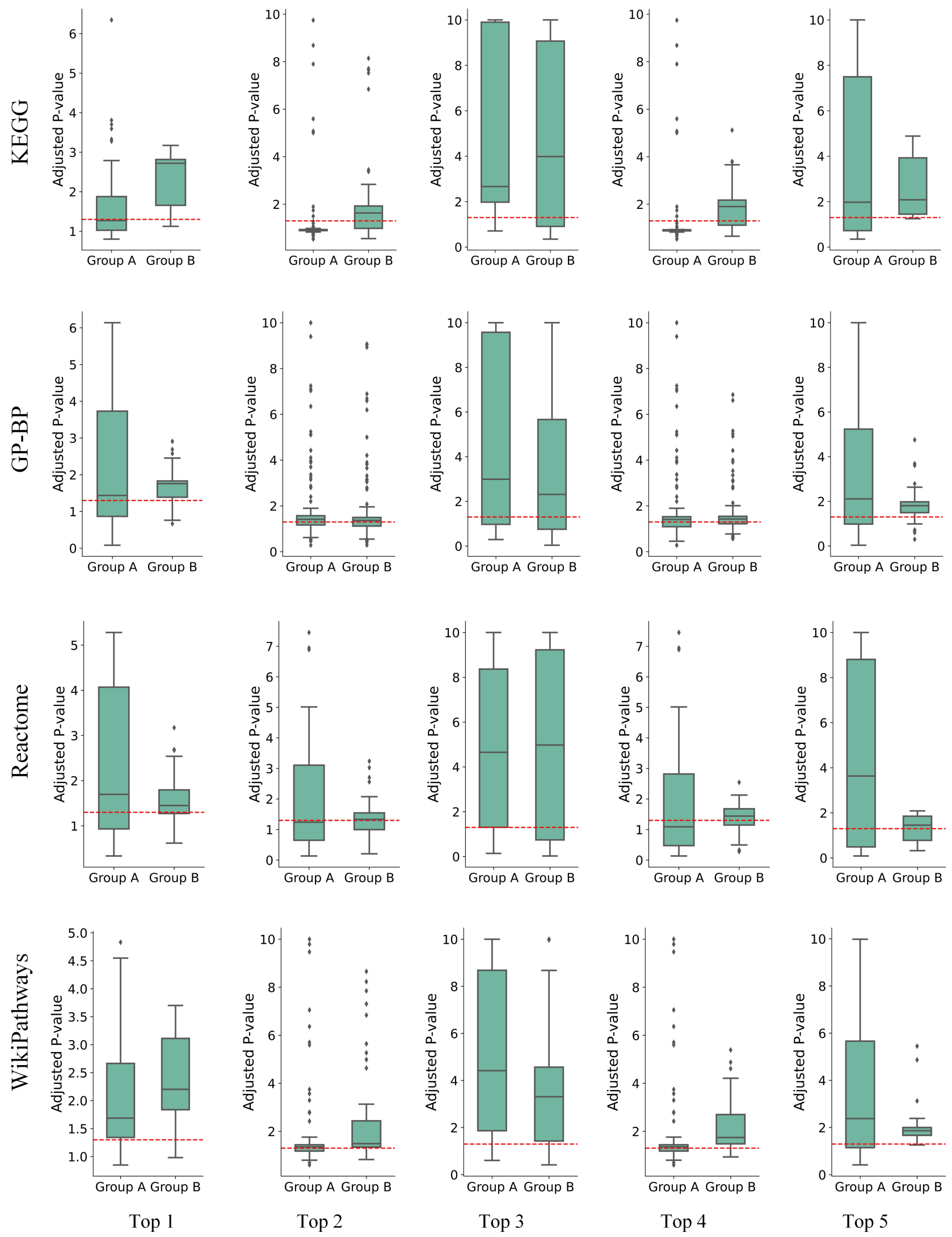


**Fig. S3 Box plots of enrichment significance (adjusted p-values) across top-ranked 2-simplex drug pairs on the TGSA dataset.** Each row corresponds to one of the top five 2-simplex drug pair groups (Top 1–Top 5) ranked by attention scores, and each column represents a distinct pathway database (KEGG, GO Biological Process, Reactome, WikiPathways). Box plots show the distribution of adjusted p-values for Group A and Group B in each drug pair. The red dashed line marks the threshold for statistical significance (adjusted p-value = 1.3, corresponding to p = 0.05).

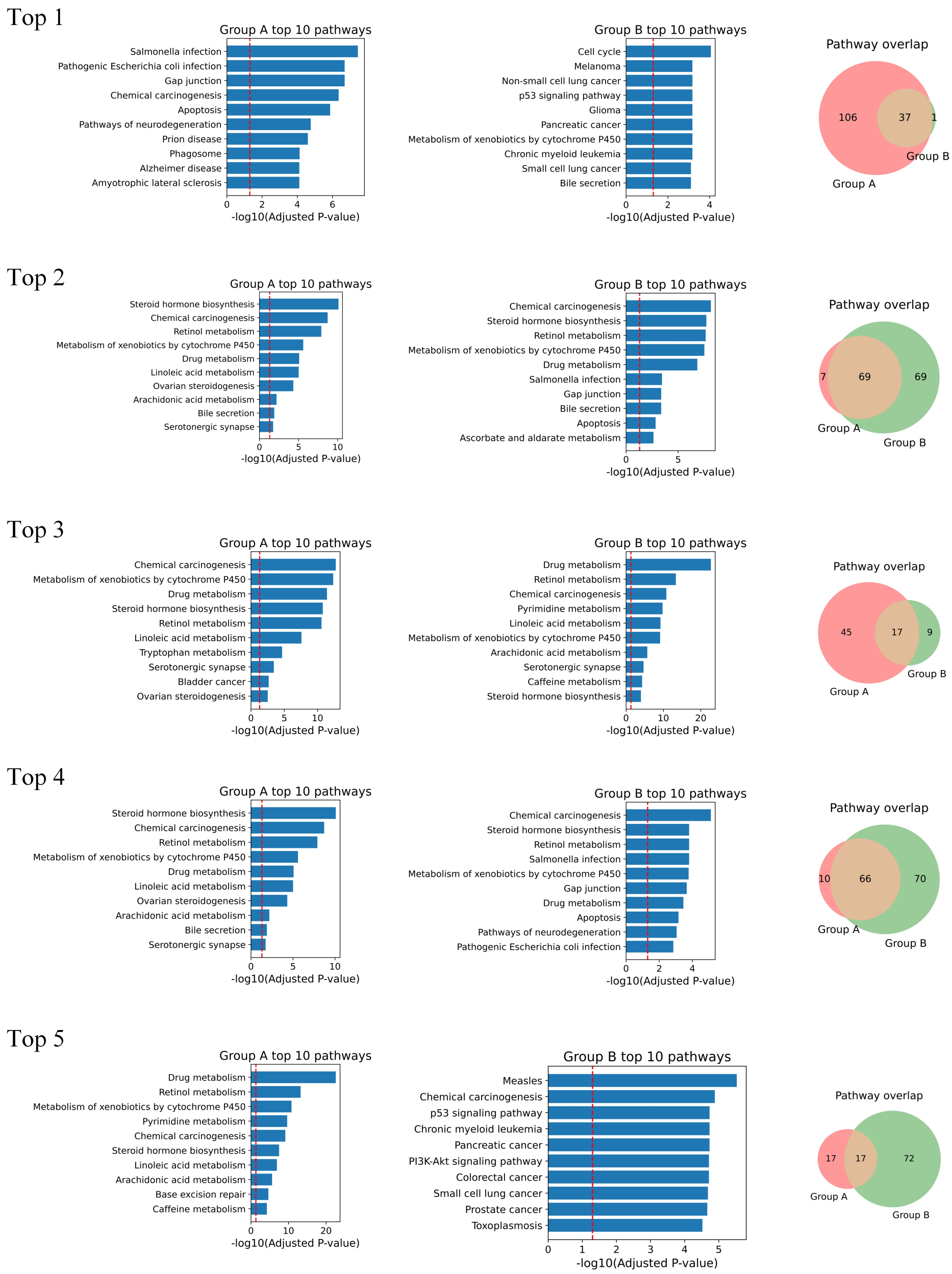


**Fig. S4 Top enriched KEGG pathways of top-ranked 2-simplex drug pairs in the TGSA dataset.** The top five 2-simplex drug pairs with the highest attention scores were selected from the attention matrix learned on the TGSA dataset. For each pair, pathway enrichment analysis was performed on drug targets using the KEGG gene set, and the top 10 enriched pathways (ranked by adjusted p-value) were shown for both Group A and Group B. The red dashed line indicates the statistical significance threshold (adjusted p-value $= 1.3$, corresponding to $p = 0.05$). The rightmost column presents Venn diagrams illustrating the overlap of enriched pathways between the two groups.

**Table S7** Pathway enrichment metrics of top-ranked 1-simplex cell line pairs across four gene set libraries

| Top N | Groups | Cell line list | Enrichment significance | | | |
|---|---|---|---|---|---|---|
| | | | **KEGG** | **GP-BP** | **Reactome** | **WikiPathways** |
| Top 1 | Group A | TYK-nu, LU-65 | 1.15 | 1.32 | 0.95 | 0.96 |
| | Group B | Hs-746T, SNG-M | 1.05 | 1.51 | 1.06 | 1.27 |
| Top 2 | Group A | HCC38, A427 | 1.36 | 1.39 | 1.31 | 0.98 |
| | Group B | HEC-1, HO-1-u-1 | 1.88 | 1.79 | 1.80 | 1.61 |
| Top 3 | Group A | HuH-7, TOV-21G | 1.25 | 1.74 | 1.17 | 2.15 |
| | Group B | Hep3B2-1-7, GOTO | 1.24 | 1.60 | 1.06 | 1.40 |
| Top 4 | Group A | SF539, TE-4 | 1.15 | 1.39 | 1.54 | 1.12 |
| | Group B | MDA-MB-468, 8505C | 1.31 | 1.60 | 1.10 | 1.93 |
| Top 5 | Group A | U-87-MG, KALS-1 | 1.30 | 1.44 | 1.34 | 1.16 |
| | Group B | LXF-289, A2058 | 2.10 | 1.71 | 1.76 | 1.37 |
| | | | **Overlapping pathways** | | | |
| | | | **KEGG** | **GP-BP** | **Reactome** | **WikiPathways** |
| Top 1 | Group A<br>Group B | TYK-nu, LU-65<br>Hs-746T, SNG-M | 7 | 121 | 42 | 5 |
| Top 2 | Group A<br>Group B | HCC38, A427<br>HEC-1, HO-1-u-1 | 23 | 232 | 97 | 8 |
| Top 3 | Group A<br>Group B | HuH-7, TOV-21G<br>Hep3B2-1-7, GOTO | 9 | 116 | 69 | 5 |
| Top 4 | Group A<br>Group B | SF539, TE-4<br>MDA-MB-468, 8505C | 5 | 110 | 39 | 4 |
| Top 5 | Group A<br>Group B | U-87-MG, KALS-1<br>LXF-289, A2058 | 20 | 236 | 93 | 16 |
| | | | **Jaccard similarity** | | | |
| | | | **KEGG** | **GP-BP** | **Reactome** | **WikiPathways** |
| Top 1 | Group A<br>Group B | TYK-nu, LU-65<br>Hs-746T, SNG-M | 0.135 | 0.301 | 0.304 | 0.172 |
| Top 2 | Group A<br>Group B | HCC38, A427<br>HEC-1, HO-1-u-1 | 0.719 | 0.554 | 0.497 | 0.222 |
| Top 3 | Group A<br>Group B | HuH-7, TOV-21G<br>Hep3B2-1-7, GOTO | 0.118 | 0.372 | 0.375 | 0.161 |
| Top 4 | Group A<br>Group B | SF539, TE-4<br>MDA-MB-468, 8505C | 0.083 | 0.272 | 0.205 | 0.071 |
| Top 5 | Group A<br>Group B | U-87-MG, KALS-1<br>LXF-289, A2058 | 0.625 | 0.576 | 0.517 | 0.444 |

**Table S8** Pathway enrichment metrics of top-ranked 2-simplex cell line pairs across four gene set libraries

| Top N | Groups | Cell line list | Enrichment significance | | | |
|---|---|---|---|---|---|---|
| | | | **KEGG** | **GP-BP** | **Reactome** | **WikiPathways** |
| Top 1 | Group A | NCI-H1693, IST-MES1, SNU-C1 | 1.93 | 1.85 | 2.13 | 1.67 |
| | Group B | HSC-4, LS-180, SW982 | 1.17 | 1.49 | 1.27 | 1.27 |
| Top 2 | Group A | MFE-280, SW900, SNG-M | 1.12 | 1.45 | 1.42 | 1.43 |
| | Group B | BHT-101, NCI-H3122, HCC202 | 0.99 | 1.46 | 1.23 | 1.07 |
| Top 3 | Group A | Calu-6, HCC70, OC-314 | 0.95 | 1.44 | 1.26 | 1.24 |
| | Group B | CAPAN-2, TYK-nu, LU-65 | 1.05 | 1.35 | 1.15 | 1.06 |
| Top 4 | Group A | CFPAC-1, NCI-H211, HCC1569 | 1.51 | 1.36 | 1.48 | 1.10 |
| | Group B | RD, NCI-H209, IST-MES1 | 1.70 | 1.67 | 1.80 | 1.46 |
| Top 5 | Group A | NCI-H69, SNU-423, NCI-H1623 | 1.58 | 1.52 | 1.64 | 1.33 |
| | Group B | TYK-nu, LU-65, FLO-1 | 1.10 | 1.37 | 1.19 | 0.97 |
| | | | **Overlapping pathways** | | | |
| | | | **KEGG** | **GP-BP** | **Reactome** | **WikiPathways** |
| Top 1 | Group A<br>Group B | NCI-H1693, IST-MES1, SNU-C1<br>HSC-4, LS-180, SW982 | 26 | 256 | 124 | 12 |
| Top 2 | Group A<br>Group B | MFE-280, SW900, SNG-M<br>BHT-101, NCI-H3122, HCC202 | 27 | 231 | 126 | 11 |
| Top 3 | Group A<br>Group B | Calu-6, HCC70, OC-314<br>CAPAN-2, TYK-nu, LU-65 | 27 | 236 | 106 | 12 |
| Top 4 | Group A<br>Group B | CFPAC-1, NCI-H211, HCC1569<br>RD, NCI-H209, IST-MES1 | 27 | 264 | 121 | 12 |
| Top 5 | Group A<br>Group B | NCI-H69, SNU-423, NCI-H1623<br>TYK-nu, LU-65, FLO-1 | 26 | 255 | 102 | 9 |
| | | | **Jaccard similarity** | | | |
| | | | **KEGG** | **GP-BP** | **Reactome** | **WikiPathways** |
| Top 1 | Group A<br>Group B | NCI-H1693, IST-MES1, SNU-C1<br>HSC-4, LS-180, SW982 | 0.366 | 0.821 | 0.642 | 0.308 |
| Top 2 | Group A<br>Group B | MFE-280, SW900, SNG-M<br>BHT-101, NCI-H3122, HCC202 | 0.290 | 0.562 | 0.516 | 0.169 |
| Top 3 | Group A<br>Group B | Calu-6, HCC70, OC-314<br>CAPAN-2, TYK-nu, LU-65 | 0.818 | 0.589 | 0.582 | 0.429 |
| Top 4 | Group A<br>Group B | CFPAC-1, NCI-H211, HCC1569<br>RD, NCI-H209, IST-MES1 | 0.252 | 0.523 | 0.465 | 0.154 |
| Top 5 | Group A<br>Group B | NCI-H69, SNU-423, NCI-H1623<br>TYK-nu, LU-65, FLO-1 | 0.329 | 0.560 | 0.507 | 0.170 |

**Table S9** Performance Comparison under Perturbed Molecular Fingerprints (20% Random Noise)

| Metric | Methods | TGSA | GDSC1 | GDSC2 | CCLE | CTRP1 | CTRP2 |
|---|---|---|---|---|---|---|---|
| PCC | MultiDRP | $0.9323_{(0.004)}$ | $0.9239_{(0.007)}$ | $\mathbf{0.9409}_{(0.004)}$ | $0.8761_{(0.003)}$ | $0.8063_{(0.004)}$ | $0.8992_{(0.005)}$ |
| | MSDRP | $0.9088_{(0.002)}$ | $0.9237_{(0.004)}$ | $0.9358_{(0.005)}$ | $0.8683_{(0.002)}$ | $0.8338_{(0.003)}$ | $0.8926_{(0.004)}$ |
| | A-DGN | $0.9220_{(0.007)}$ | $0.9148_{(0.010)}$ | $0.9299_{(0.008)}$ | $0.8706_{(0.001)}$ | $0.7956_{(0.007)}$ | $0.8749_{(0.008)}$ |
| | ARMA | $0.8760_{(0.011)}$ | $0.9295_{(0.007)}$ | $0.9351_{(0.003)}$ | $0.8760_{(0.002)}$ | $0.7976_{(0.007)}$ | $0.8962_{(0.008)}$ |
| | EGC | $0.9299_{(0.005)}$ | $0.9268_{(0.003)}$ | $0.9349_{(0.004)}$ | $0.8733_{(0.007)}$ | $0.7901_{(0.004)}$ | $0.8944_{(0.002)}$ |
| | GraphGPS | $0.9334_{(0.003)}$ | $0.9293_{(0.002)}$ | $0.9382_{(0.006)}$ | $0.8792_{(0.014)}$ | $0.8206_{(0.003)}$ | $0.8992_{(0.005)}$ |
| | SSGC | $0.9249_{(0.005)}$ | $0.9124_{(0.007)}$ | $0.9290_{(0.007)}$ | $0.8729_{(0.008)}$ | $0.8012_{(0.004)}$ | $0.8791_{(0.006)}$ |
| | HGNN | $0.9017_{(0.002)}$ | $0.8857_{(0.006)}$ | $0.9006_{(0.003)}$ | $0.8484_{(0.006)}$ | $0.6158_{(0.008)}$ | $0.8563_{(0.003)}$ |
| | HGNN$^{+}$ | $0.8248_{(0.008)}$ | $0.8204_{(0.012)}$ | $0.7682_{(0.005)}$ | $0.7465_{(0.003)}$ | $0.4437_{(0.008)}$ | $0.8131_{(0.004)}$ |
| | BScNets | $0.9038_{(0.007)}$ | $0.8898_{(0.006)}$ | $0.9059_{(0.008)}$ | $0.8725_{(0.004)}$ | $0.7316_{(0.004)}$ | $0.8462_{(0.004)}$ |
| | HiGCN | $0.8884_{(0.002)}$ | $0.8975_{(0.007)}$ | $0.9114_{(0.002)}$ | $0.8661_{(0.005)}$ | $0.7045_{(0.005)}$ | $0.8533_{(0.007)}$ |
| | TopDr | $\mathbf{0.9401}_{(0.003)}$ | $\mathbf{0.9306}_{(0.006)}$ | $0.9397_{(0.003)}$ | $\mathbf{0.8785}_{(0.002)}$ | $\mathbf{0.8256}_{(0.005)}$ | $\mathbf{0.9006}_{(0.004)}$ |
| RMSE | MultiDRP | $1.0252_{(0.022)}$ | $1.0257_{(0.018)}$ | $0.9659_{(0.041)}$ | $0.7551_{(0.025)}$ | $1.0133_{(0.014)}$ | $1.1495_{(0.022)}$ |
| | MSDRP | $1.1868_{(0.026)}$ | $1.0195_{(0.022)}$ | $1.0014_{(0.033)}$ | $0.7820_{(0.034)}$ | $0.9521_{(0.010)}$ | $1.1913_{(0.017)}$ |
| | A-DGN | $1.0991_{(0.017)}$ | $1.0852_{(0.023)}$ | $1.0499_{(0.020)}$ | $0.7741_{(0.017)}$ | $1.0383_{(0.021)}$ | $1.2781_{(0.029)}$ |
| | ARMA | $0.9944_{(0.019)}$ | $0.9879_{(0.037)}$ | $1.0118_{(0.042)}$ | $0.7544_{(0.026)}$ | $1.0317_{(0.027)}$ | $1.1735_{(0.018)}$ |
| | EGC | $1.0432_{(0.021)}$ | $1.0071_{(0.031)}$ | $1.0139_{(0.039)}$ | $0.7899_{(0.018)}$ | $1.0514_{(0.025)}$ | $1.1760_{(0.020)}$ |
| | GraphGPS | $1.0177_{(0.022)}$ | $0.9894_{(0.014)}$ | $0.9881_{(0.027)}$ | $0.7570_{(0.051)}$ | $0.9769_{(0.017)}$ | $1.1504_{(0.035)}$ |
| | SSGC | $1.0789_{(0.025)}$ | $1.0959_{(0.027)}$ | $1.0575_{(0.016)}$ | $0.7651_{(0.012)}$ | $1.0234_{(0.024)}$ | $1.2554_{(0.030)}$ |
| | HGNN | $1.2263_{(0.018)}$ | $1.2360_{(0.022)}$ | $1.2382_{(0.024)}$ | $0.8341_{(0.022)}$ | $1.3609_{(0.008)}$ | $1.3640_{(0.016)}$ |
| | HGNN$^{+}$ | $1.6047_{(0.046)}$ | $1.5285_{(0.021)}$ | $1.8211_{(0.035)}$ | $1.0488_{(0.018)}$ | $1.5496_{(0.033)}$ | $1.5493_{(0.018)}$ |
| | BScNets | $1.2161_{(0.050)}$ | $1.2156_{(0.027)}$ | $1.2069_{(0.012)}$ | $0.7704_{(0.014)}$ | $1.1747_{(0.038)}$ | $1.4084_{(0.040)}$ |
| | HiGCN | $1.3389_{(0.032)}$ | $1.1745_{(0.019)}$ | $1.1771_{(0.030)}$ | $0.7895_{(0.025)}$ | $1.2481_{(0.039)}$ | $1.3885_{(0.015)}$ |
| | TopDr | $\mathbf{0.9635}_{(0.031)}$ | $\mathbf{0.9788}_{(0.024)}$ | $\mathbf{0.9771}_{(0.041)}$ | $\mathbf{0.7513}_{(0.021)}$ | $\mathbf{0.9671}_{(0.018)}$ | $\mathbf{1.1402}_{(0.034)}$ |
| MAE | MultiDRP | $0.7617_{(0.022)}$ | $0.7642_{(0.019)}$ | $0.7139_{(0.017)}$ | $0.4705_{(0.018)}$ | $0.7512_{(0.011)}$ | $0.8045_{(0.021)}$ |
| | MSDRP | $0.8965_{(0.018)}$ | $0.7582_{(0.006)}$ | $0.7452_{(0.010)}$ | $0.4783_{(0.012)}$ | $0.6980_{(0.026)}$ | $0.8276_{(0.015)}$ |
| | A-DGN | $0.8220_{(0.011)}$ | $0.8146_{(0.022)}$ | $0.7875_{(0.005)}$ | $0.4843_{(0.028)}$ | $0.7885_{(0.024)}$ | $0.9230_{(0.018)}$ |
| | ARMA | $0.7721_{(0.013)}$ | $0.7312_{(0.014)}$ | $0.7535_{(0.012)}$ | $0.4721_{(0.031)}$ | $0.7732_{(0.013)}$ | $0.8307_{(0.011)}$ |
| | EGC | $0.7808_{(0.010)}$ | $0.7459_{(0.014)}$ | $0.7550_{(0.031)}$ | $0.4805_{(0.014)}$ | $0.7885_{(0.014)}$ | $0.8332_{(0.031)}$ |
| | GraphGPS | $0.7591_{(0.009)}$ | $0.7393_{(0.018)}$ | $0.7374_{(0.024)}$ | $0.4702_{(0.021)}$ | $0.7339_{(0.012)}$ | $0.8161_{(0.027)}$ |
| | SSGC | $0.8113_{(0.016)}$ | $0.8217_{(0.022)}$ | $0.7915_{(0.016)}$ | $0.4817_{(0.028)}$ | $0.7738_{(0.022)}$ | $0.8951_{(0.014)}$ |
| | HGNN | $0.9171_{(0.013)}$ | $0.9290_{(0.011)}$ | $0.9376_{(0.018)}$ | $0.5050_{(0.022)}$ | $1.0598_{(0.025)}$ | $0.9516_{(0.025)}$ |
| | HGNN$^{+}$ | $1.2531_{(0.019)}$ | $1.1639_{(0.016)}$ | $1.4146_{(0.015)}$ | $0.5996_{(0.014)}$ | $1.2362_{(0.022)}$ | $1.1280_{(0.013)}$ |
| | BScNets | $0.9158_{(0.015)}$ | $0.9063_{(0.013)}$ | $0.9042_{(0.016)}$ | $0.4721_{(0.015)}$ | $0.8891_{(0.016)}$ | $0.9853_{(0.014)}$ |
| | HiGCN | $1.0353_{(0.021)}$ | $0.8735_{(0.011)}$ | $0.8790_{(0.012)}$ | $0.4823_{(0.007)}$ | $0.9719_{(0.018)}$ | $0.9712_{(0.021)}$ |
| | TopDr | $\mathbf{0.7208}_{(0.001)}$ | $\mathbf{0.7215}_{(0.001)}$ | $\mathbf{0.7221}_{(0.001)}$ | $\mathbf{0.4699}_{(0.001)}$ | $\mathbf{0.7142}_{(0.001)}$ | $\mathbf{0.7988}_{(0.001)}$ |

**Table S10** Performance comparison under cross-cell line and cross-drug settings.

| Setting | Metric | Method | TGSA | GDSC1 | GDSC2 | CCLE | CTRP1 | CTRP2 |
|---|---|---|---|---|---|---|---|---|
| Cross-cell | PCC | TopDr | 0.8072 | 0.8565 | 0.9370 | 0.8584 | 0.6394 | 0.7538 |
| | | MultiDRP | 0.7941 | 0.8442 | 0.8336 | 0.8400 | 0.5871 | 0.7462 |
| | RMSE | TopDr | 1.7090 | 1.4232 | 1.6019 | 0.8783 | 1.3627 | 1.7923 |
| | | MultiDRP | 1.7344 | 1.4801 | 1.6649 | 0.9185 | 1.4162 | 1.8272 |
| | MAE | TopDr | 1.2605 | 1.0610 | 1.2469 | 0.4951 | 1.0643 | 1.2581 |
| | | MultiDRP | 1.3159 | 1.1142 | 1.2674 | 0.5365 | 1.0926 | 1.3044 |
| Cross-drug | PCC | TopDr | 0.4217 | 0.4180 | 0.6855 | 0.4500 | 0.3622 | 0.3894 |
| | | MultiDRP | 0.3925 | 0.4076 | 0.6673 | 0.4365 | 0.3588 | 0.3674 |
| | RMSE | TopDr | 2.2170 | 1.9595 | 1.9946 | 1.0310 | 1.8939 | 3.0689 |
| | | MultiDRP | 2.3513 | 2.0489 | 2.1294 | 1.2139 | 2.0012 | 3.6905 |
| | MAE | TopDr | 1.5358 | 1.5734 | 1.5923 | 0.8391 | 1.5981 | 2.2655 |
| | | MultiDRP | 1.6987 | 1.7921 | 1.6881 | 0.9128 | 1.7283 | 2.3589 |

**Table S11** Parameter sensitivity analysis with respect to the number of nearest neighbors $k$ in kNN-based construction.

| Metric | $k$ | TGSA | GDSC1 | GDSC2 | CCLE | CTRP1 | CTRP2 |
|---|---|---|---|---|---|---|---|
| PCC | $k = 5$ | 0.9407 | 0.9297 | 0.9380 | 0.8781 | 0.8224 | 0.8950 |
| | $k = 10$ | 0.9405 | 0.9291 | 0.9394 | 0.8758 | 0.8256 | 0.8887 |
| | $k = 15$ | 0.9389 | 0.9266 | 0.9366 | 0.8746 | 0.8214 | 0.8979 |
| | $k = 20$ | 0.9387 | 0.9259 | 0.9374 | 0.8789 | 0.8173 | 0.8951 |
| RMSE | $k = 5$ | 0.9602 | 0.9885 | 0.9879 | 0.7488 | 0.9811 | 1.1728 |
| | $k = 10$ | 0.9663 | 0.9923 | 0.9785 | 0.7581 | 0.9676 | 1.2046 |
| | $k = 15$ | 0.9778 | 1.0069 | 0.9986 | 0.7600 | 0.9816 | 1.1557 |
| | $k = 20$ | 0.9756 | 1.0124 | 0.9923 | 0.7476 | 0.9936 | 1.1720 |
| MAE | $k = 5$ | 0.7117 | 0.7309 | 0.7331 | 0.4684 | 0.7158 | 0.8196 |
| | $k = 10$ | 0.7135 | 0.7366 | 0.7308 | 0.4725 | 0.7104 | 0.8398 |
| | $k = 15$ | 0.7203 | 0.7488 | 0.7434 | 0.4735 | 0.7275 | 0.8080 |
| | $k = 20$ | 0.7157 | 0.7494 | 0.7366 | 0.4680 | 0.7293 | 0.8199 |

**Table S12** Sensitivity analysis of the RBF center range $c_l$ for 1-simplex feature initialization.

| Metric | $c_l$ range | TGSA | GDSC1 | GDSC2 | CCLE | CTRP1 | CTRP2 |
|---|---|---|---|---|---|---|---|
| PCC | [0,2] | 0.9390 | 0.9320 | 0.9405 | 0.8772 | 0.8186 | 0.9037 |
| | [0,4] | 0.9388 | 0.9282 | 0.9417 | 0.8791 | 0.8236 | 0.8979 |
| | [0,6] | 0.9406 | 0.9279 | 0.9408 | 0.8788 | 0.8157 | 0.9009 |
| | [0,8] | 0.9407 | 0.9322 | 0.9406 | 0.8779 | 0.8308 | 0.9024 |
| | [0,10] | 0.9418 | 0.9318 | 0.9423 | 0.8791 | 0.8319 | 0.9026 |
| RMSE | [0,2] | 0.9761 | 0.9730 | 0.9688 | 0.7524 | 0.9844 | 1.1255 |
| | [0,4] | 0.9761 | 0.9826 | 0.9589 | 0.7453 | 0.9756 | 1.1664 |
| | [0,6] | 0.9653 | 0.9849 | 0.9671 | 0.7480 | 0.9904 | 1.1398 |
| | [0,8] | 0.9600 | 0.9713 | 0.9681 | 0.7487 | 0.9560 | 1.1338 |
| | [0,10] | 0.9484 | 0.9762 | 0.9608 | 0.7454 | 0.9562 | 1.1331 |
| MAE | [0,2] | 0.7213 | 0.7197 | 0.7159 | 0.4678 | 0.7221 | 0.7835 |
| | [0,4] | 0.7157 | 0.7268 | 0.7089 | 0.4678 | 0.7170 | 0.8128 |
| | [0,6] | 0.7134 | 0.7276 | 0.7149 | 0.4683 | 0.7283 | 0.7939 |
| | [0,8] | 0.7185 | 0.7179 | 0.7182 | 0.4668 | 0.7044 | 0.7875 |
| | [0,10] | 0.7114 | 0.7187 | 0.7109 | 0.4671 | 0.7058 | 0.7900 |

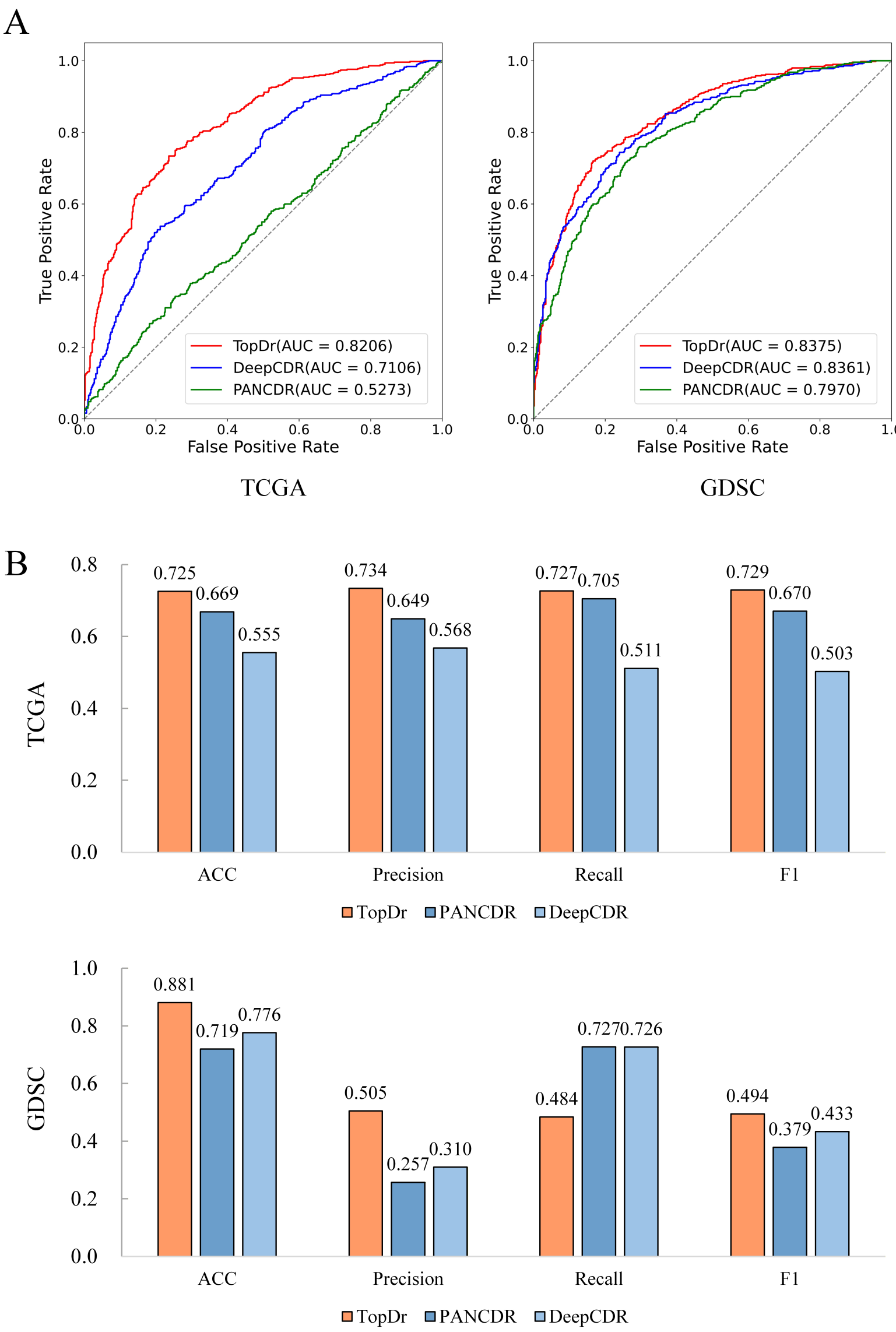


**Fig. S5 Performance comparison on discrete drug response phenotype prediction. (A)** ROC curves showing AUC for TopDr, PANCDR, and DeepCDR on TCGA and GDSC datasets. **(B)** Bar plots of classification metrics (ACC, Precision, Recall, and F1 score) across the three models on TCGA (top) and GDSC (bottom).

**Table S13** Sensitivity analysis of the RBF center range $c_l$ for 2-simplex feature initialization.

| Metric | $c_l$ range | TGSA | GDSC1 | GDSC2 | CCLE | CTRP1 | CTRP2 |
|---|---|---|---|---|---|---|---|
| PCC | [0,2] | 0.9415 | 0.9314 | 0.9406 | 0.8766 | 0.8224 | 0.9004 |
| | [0,4] | 0.9418 | 0.9318 | 0.9423 | 0.8791 | 0.8319 | 0.9026 |
| | [0,6] | 0.9398 | 0.9316 | 0.9406 | 0.8778 | 0.8277 | 0.9003 |
| | [0,8] | 0.9409 | 0.9305 | 0.9417 | 0.8759 | 0.8185 | 0.9420 |
| | [0,10] | 0.9405 | 0.9310 | 0.9413 | 0.8775 | 0.8305 | 0.8991 |
| RMSE | [0,2] | 0.9553 | 0.9776 | 0.9714 | 0.7522 | 0.9806 | 1.1421 |
| | [0,4] | 0.9484 | 0.9762 | 0.9608 | 0.7454 | 0.9562 | 1.1331 |
| | [0,6] | 0.9680 | 0.9741 | 0.9679 | 0.7502 | 0.9696 | 1.1443 |
| | [0,8] | 0.9622 | 0.9809 | 0.9601 | 0.7556 | 0.9956 | 0.9626 |
| | [0,10] | 0.9663 | 0.9783 | 0.9634 | 0.7559 | 0.9558 | 1.1506 |
| MAE | [0,2] | 0.7139 | 0.7214 | 0.7186 | 0.4686 | 0.7130 | 0.7949 |
| | [0,4] | 0.7114 | 0.7187 | 0.7109 | 0.4671 | 0.7058 | 0.7900 |
| | [0,6] | 0.7105 | 0.7172 | 0.7215 | 0.4693 | 0.7092 | 0.7973 |
| | [0,8] | 0.7159 | 0.7242 | 0.7085 | 0.4709 | 0.7337 | 0.7128 |
| | [0,10] | 0.7135 | 0.7227 | 0.7174 | 0.4720 | 0.7110 | 1.3239 |

**Table S14** Sensitivity analysis of the RBF step size in feature initialization.

| Metric | Step | TGSA | GDSC1 | GDSC2 | CCLE | CTRP1 | CTRP2 |
|---|---|---|---|---|---|---|---|
| PCC | 0.05 | 0.9408 | 0.9310 | 0.9417 | 0.8782 | 0.8262 | 0.9027 |
| | 0.1 | 0.9418 | 0.9318 | 0.9423 | 0.8791 | 0.8319 | 0.9026 |
| | 0.2 | 0.9403 | 0.9314 | 0.9419 | 0.8748 | 0.8307 | 0.8995 |
| | 0.3 | 0.9415 | 0.9306 | 0.9413 | 0.8770 | 0.8183 | 0.9022 |
| | 0.4 | 0.9408 | 0.9318 | 0.9417 | 0.8790 | 0.8212 | 0.9026 |
| | 0.5 | 0.9406 | 0.9294 | 0.9411 | 0.8804 | 0.8266 | 0.9032 |
| RMSE | 0.05 | 0.9624 | 0.9790 | 0.9603 | 0.7480 | 0.9710 | 1.1350 |
| | 0.1 | 0.9484 | 0.9762 | 0.9608 | 0.7454 | 0.9562 | 1.1331 |
| | 0.2 | 0.9639 | 0.9752 | 0.9577 | 0.7592 | 0.9590 | 1.1501 |
| | 0.3 | 0.9558 | 0.9810 | 0.9633 | 0.7520 | 0.9856 | 1.1357 |
| | 0.4 | 0.9617 | 0.9731 | 0.9614 | 0.7521 | 0.9825 | 1.1285 |
| | 0.5 | 0.9561 | 0.9736 | 0.9656 | 0.7416 | 0.9725 | 1.1269 |
| MAE | 0.05 | 0.7057 | 0.7235 | 0.7090 | 0.4657 | 0.7167 | 0.7898 |
| | 0.1 | 0.7114 | 0.7187 | 0.7109 | 0.4671 | 0.7058 | 0.7900 |
| | 0.2 | 0.7139 | 0.7203 | 0.7115 | 0.4716 | 0.7023 | 0.8028 |
| | 0.3 | 0.7101 | 0.7265 | 0.7128 | 0.4727 | 0.7204 | 0.7950 |
| | 0.4 | 0.7106 | 0.7182 | 0.7118 | 0.4708 | 0.7278 | 0.7895 |
| | 0.5 | 0.7132 | 0.7240 | 0.7134 | 0.4653 | 0.7224 | 0.7885 |

**Table S15** Summary of drug response statistics across six pharmacogenomic datasets

| Dataset | Number of Drugs | Number of Cell Lines | Number of Drug Responses |
|---|---|---|---|
| TGSA | 170 | 580 | 82,833 |
| GDSC1 | 312 | 946 | 271,705 |
| GDSC2 | 233 | 944 | 198,457 |
| CCLE | 1267 | 221 | 278,597 |
| CTRP1 | 203 | 240 | 30,194 |
| CTRP2 | 545 | 821 | 364,774 |
