# Supplementary material for: Topology‐Aware Deep Learning on Higher‐Order Structures for Drug Response Prediction
Source: Adv Sci (Weinh). 2026 May 27:e75816. Online ahead of print. doi: 10.1002/advs.75816 (PMC13335842; doi:10.1002/advs.75816)
Supplement: Supplementary file 1 — Supporting File: advs75816‐sup‐0001‐SuppMat.pdf. [file ADVS-9999-e75816-s001.pdf]

Supplementary Material:

Topology-Aware Deep Learning on Higher-Order Structures for Drug Response Prediction

Cong Shen[1*], Guancen Lin[1], Chuan-Shen Hu[2], Yong Wang[1,3,4,5*]

[1*]State Key Laboratory of Mathematical Sciences, Academy of Mathematics and Systems Science, Chinese Academy of Sciences, Beijing, 100190, Beijing, China.
[2]Department of Applied Mathematics, National University of Kaohsiung, Kaohsiung, 81148, Kaohsiung, Taiwan.
[3]School of Mathematics, University of Chinese Academy of Sciences, Chinese Academy of Sciences, Beijing, 100049, Beijing, China.
[4]Center for Excellence in Animal Evolution and Genetics, Chinese Academy of Sciences, Kunming, 650223, Yunnan, China.
[5]Key Laboratory of Systems Biology, Hangzhou Institute for Advanced Study, University of Chinese Academy of Sciences, Chinese Academy of Sciences, Hangzhou, 330106, Zhejiang, China.

*Corresponding author(s). E-mail(s): cshen@amss.ac.cn; ywang@amss.ac.cn;
Contributing authors: linguancen@amss.ac.cn; chuanshenhu1@nuk.edu.tw;

Table S1 Performance comparison of different methods on six datasets under three evaluation metrics. For PCC, higher values indicate better performance, while for RMSE and MAE, lower values are preferred. The best results are highlighted in bold. Values in parentheses denote the standard deviation over five runs.

Metric	Methods	TGSA	GDSC1	GDSC2	CCLE	CTRP1	CTRP2
PCC	MultiDRP	0.9354$_{(0.001)}$	0.9209$_{(0.003)}$	0.9349$_{(0.003)}$	0.8760$_{(0.001)}$	0.7962$_{(0.002)}$	0.8801$_{(0.002)}$
	MSDRP	0.9335$_{(0.006)}$	0.9225$_{(0.001)}$	0.9369$_{(0.001)}$	0.8722$_{(0.002)}$	0.8194$_{(0.001)}$	0.8954$_{(0.003)}$
	PANCDR	0.8966$_{(0.000)}$	0.9076$_{(0.001)}$	0.9352$_{(0.002)}$	0.8637$_{(0.001)}$	0.7083$_{(0.001)}$	0.8977$_{(0.002)}$
	GraphCDR	0.8957$_{(0.002)}$	0.8844$_{(0.004)}$	0.9055$_{(0.002)}$	0.8234$_{(0.004)}$	0.7257$_{(0.000)}$	0.7556$_{(0.004)}$
	SubCDR	0.9205$_{(0.001)}$	0.9264$_{(0.000)}$	0.9327$_{(0.003)}$	0.8611$_{(0.002)}$	0.7735$_{(0.001)}$	0.8715$_{(0.001)}$
	A-DGN	0.9310$_{(0.001)}$	0.9129$_{(0.004)}$	0.9318$_{(0.001)}$	0.8687$_{(0.002)}$	0.8026$_{(0.002)}$	0.8771$_{(0.001)}$
	ARMA	0.9295$_{(0.003)}$	0.9258$_{(0.001)}$	0.9372$_{(0.002)}$	0.8761$_{(0.001)}$	0.7983$_{(0.002)}$	0.8980$_{(0.003)}$
	EGC	0.9295$_{(0.004)}$	0.9266$_{(0.002)}$	0.9345$_{(0.004)}$	0.8743$_{(0.003)}$	0.8028$_{(0.001)}$	0.8956$_{(0.002)}$
	GraphGPS	0.9353$_{(0.005)}$	0.9263$_{(0.003)}$	0.9402$_{(0.002)}$	0.8761$_{(0.003)}$	0.8159$_{(0.005)}$	0.9025$_{(0.002)}$
	SSGC	0.9236$_{(0.000)}$	0.9106$_{(0.003)}$	0.9294$_{(0.000)}$	0.8694$_{(0.000)}$	0.8027$_{(0.002)}$	0.8798$_{(0.004)}$
	HGNN	0.9063$_{(0.002)}$	0.8910$_{(0.004)}$	0.9041$_{(0.002)}$	0.8517$_{(0.001)}$	0.6991$_{(0.001)}$	0.8609$_{(0.004)}$
	HGNN^{+}	0.8626$_{(0.003)}$	0.8597$_{(0.002)}$	0.8685$_{(0.001)}$	0.8150$_{(0.001)}$	0.5033$_{(0.004)}$	0.8175$_{(0.002)}$
	BScNets	0.9069$_{(0.003)}$	0.9007$_{(0.000)}$	0.9119$_{(0.001)}$	0.8730$_{(0.003)}$	0.7327$_{(0.003)}$	0.8504$_{(0.001)}$
	HiGCN	0.9009$_{(0.001)}$	0.9010$_{(0.001)}$	0.9155$_{(0.004)}$	0.8717$_{(0.005)}$	0.7126$_{(0.002)}$	0.8387$_{(0.003)}$
	MolGT	0.8708$_{(0.005)}$	0.8873$_{(0.000)}$	0.8974$_{(0.002)}$	0.8308$_{(0.004)}$	0.6678$_{(0.004)}$	0.8108$_{(0.001)}$
	TopDr	**0.9418**$_{(0.002)}$	**0.9318**$_{(0.001)}$	**0.9423**$_{(0.000)}$	**0.8791**$_{(0.001)}$	**0.8319**$_{(0.001)}$	**0.9026**$_{(0.003)}$
RMSE	MultiDRP	1.0013$_{(0.009)}$	1.0441$_{(0.012)}$	1.0153$_{(0.030)}$	0.7539$_{(0.023)}$	1.0354$_{(0.012)}$	1.2539$_{(0.022)}$
	MSDRP	1.0005$_{(0.012)}$	1.0274$_{(0.031)}$	0.9956$_{(0.029)}$	0.7719$_{(0.018)}$	0.9900$_{(0.024)}$	1.1764$_{(0.015)}$
	PANCDR	1.3047$_{(0.021)}$	1.1240$_{(0.030)}$	1.0243$_{(0.014)}$	0.8106$_{(0.015)}$	1.2346$_{(0.015)}$	1.1582$_{(0.013)}$
	GraphCDR	1.4247$_{(0.010)}$	1.2358$_{(0.022)}$	1.2572$_{(0.012)}$	0.9679$_{(0.022)}$	1.1950$_{(0.016)}$	1.7349$_{(0.028)}$
	SubCDR	1.2384$_{(0.015)}$	1.0200$_{(0.018)}$	1.0549$_{(0.012)}$	**0.6636**$_{(0.009)}$	1.2058$_{(0.014)}$	1.6836$_{(0.022)}$
	A-DGN	1.0331$_{(0.018)}$	1.0783$_{(0.012)}$	1.0411$_{(0.030)}$	0.8021$_{(0.017)}$	1.0216$_{(0.014)}$	1.2617$_{(0.007)}$
	ARMA	1.0456$_{(0.015)}$	0.9967$_{(0.020)}$	1.0015$_{(0.028)}$	0.7812$_{(0.014)}$	1.0378$_{(0.017)}$	1.1592$_{(0.016)}$
	EGC	1.0456$_{(0.019)}$	1.0078$_{(0.009)}$	1.0171$_{(0.016)}$	0.7867$_{(0.023)}$	1.0217$_{(0.024)}$	1.1729$_{(0.021)}$
	GraphGPS	0.9995$_{(0.006)}$	0.9951$_{(0.014)}$	0.9770$_{(0.005)}$	0.7801$_{(0.016)}$	0.9895$_{(0.020)}$	**1.1316**$_{(0.027)}$
	SSGC	1.0837$_{(0.008)}$	1.0922$_{(0.017)}$	1.0586$_{(0.007)}$	0.8005$_{(0.003)}$	1.0185$_{(0.010)}$	1.2530$_{(0.016)}$
	HGNN	1.2029$_{(0.022)}$	1.2086$_{(0.026)}$	1.2137$_{(0.016)}$	0.8264$_{(0.012)}$	1.2324$_{(0.025)}$	1.3449$_{(0.010)}$
	HGNN^{+}	1.4390$_{(0.016)}$	1.3595$_{(0.022)}$	1.4099$_{(0.021)}$	0.9133$_{(0.033)}$	1.4897$_{(0.013)}$	1.5218$_{(0.011)}$
	BScNets	1.1939$_{(0.011)}$	1.1573$_{(0.028)}$	1.1659$_{(0.028)}$	0.7683$_{(0.018)}$	1.1735$_{(0.013)}$	1.3943$_{(0.022)}$
	HiGCN	1.2811$_{(0.014)}$	1.1565$_{(0.015)}$	1.1478$_{(0.017)}$	0.7752$_{(0.009)}$	1.2391$_{(0.016)}$	1.4641$_{(0.020)}$
	MolGT	1.4012$_{(0.007)}$	1.2501$_{(0.011)}$	1.2273$_{(0.013)}$	0.9284$_{(0.007)}$	1.3266$_{(0.021)}$	1.5651$_{(0.013)}$
	TopDr	**0.9484**$_{(0.016)}$	**0.9762**$_{(0.012)}$	**0.9608**$_{(0.009)}$	0.7454$_{(0.011)}$	**0.9562**$_{(0.013)}$	1.1331$_{(0.005)}$
MAE	MultiDRP	0.7346$_{(0.004)}$	0.7750$_{(0.007)}$	0.7515$_{(0.019)}$	0.4688$_{(0.006)}$	0.7745$_{(0.012)}$	0.8769$_{(0.011)}$
	MSDRP	0.7409$_{(0.015)}$	0.7639$_{(0.012)}$	0.7425$_{(0.022)}$	0.4735$_{(0.012)}$	0.7404$_{(0.022)}$	0.8184$_{(0.017)}$
	PANCDR	0.9802$_{(0.022)}$	0.8339$_{(0.024)}$	0.7762$_{(0.015)}$	0.4934$_{(0.022)}$	0.9577$_{(0.044)}$	0.8171$_{(0.019)}$
	GraphCDR	1.1301$_{(0.034)}$	0.9189$_{(0.031)}$	0.9406$_{(0.013)}$	0.6161$_{(0.029)}$	0.9041$_{(0.021)}$	1.2216$_{(0.025)}$
	SubCDR	0.8442$_{(0.021)}$	0.7566$_{(0.028)}$	0.7780$_{(0.017)}$	0.4857$_{(0.012)}$	0.8397$_{(0.022)}$	0.9221$_{(0.023)}$
	A-DGN	0.7709$_{(0.013)}$	0.8098$_{(0.013)}$	0.7820$_{(0.014)}$	0.4889$_{(0.011)}$	0.7760$_{(0.021)}$	0.9091$_{(0.013)}$
	ARMA	0.7836$_{(0.009)}$	0.7405$_{(0.019)}$	0.7464$_{(0.025)}$	0.4771$_{(0.007)}$	0.7750$_{(0.016)}$	0.8207$_{(0.015)}$
	EGC	0.7836$_{(0.015)}$	0.7486$_{(0.007)}$	0.7586$_{(0.009)}$	0.4814$_{(0.015)}$	0.7662$_{(0.014)}$	0.8314$_{(0.018)}$
	GraphGPS	0.7533$_{(0.017)}$	0.7383$_{(0.012)}$	0.7267$_{(0.011)}$	0.4791$_{(0.021)}$	0.7427$_{(0.021)}$	0.8078$_{(0.036)}$
	SSGC	0.8194$_{(0.023)}$	0.8191$_{(0.011)}$	0.7973$_{(0.015)}$	0.4876$_{(0.027)}$	0.7584$_{(0.028)}$	0.8914$_{(0.022)}$
	HGNN	0.9063$_{(0.041)}$	0.9072$_{(0.015)}$	0.9128$_{(0.026)}$	0.5013$_{(0.031)}$	0.9410$_{(0.012)}$	0.9478$_{(0.012)}$
	HGNN^{+}	1.0921$_{(0.031)}$	1.0197$_{(0.034)}$	1.0676$_{(0.028)}$	0.5426$_{(0.038)}$	1.1767$_{(0.016)}$	1.0786$_{(0.016)}$
	BScNets	0.8936$_{(0.019)}$	0.8614$_{(0.012)}$	0.8653$_{(0.019)}$	0.4702$_{(0.007)}$	0.8877$_{(0.013)}$	0.9763$_{(0.011)}$
	HiGCN	1.2811$_{(0.027)}$	0.8622$_{(0.014)}$	0.8593$_{(0.025)}$	0.4734$_{(0.006)}$	0.9643$_{(0.017)}$	1.0335$_{(0.010)}$
	MolGT	1.0905$_{(0.022)}$	0.9436$_{(0.018)}$	0.9412$_{(0.031)}$	0.5136$_{(0.013)}$	1.0992$_{(0.015)}$	1.0161$_{(0.014)}$
	TopDr	**0.7114**$_{(0.012)}$	**0.7187**$_{(0.011)}$	**0.7109**$_{(0.014)}$	**0.4671**$_{(0.009)}$	**0.7058**$_{(0.008)}$	**0.7900**$_{(0.016)}$

Table S2 Statistical significance analysis (p-values) of different models across multiple datasets. Lower p-values indicate stronger statistical significance.

Metric	Methods	TGSA	GDSC1	GDSC2	CCLE	CTRP1	CTRP2
PCC	MultiDRP	3.21E-02	8.36E-06	1.54E-03	2.87E-02	7.42E-05	4.13E-04
	MSDRP	9.12E-03	2.66E-02	5.18E-07	1.33E-04	3.75E-02	6.41E-06
	PANCDR	4.62E-04	7.25E-03	2.98E-02	6.87E-06	1.54E-02	8.12E-05
	GraphCDR	1.82E-02	9.74E-06	3.64E-02	7.11E-04	2.05E-03	5.77E-03
	SubCDR	6.55E-03	1.24E-04	8.17E-06	3.92E-02	7.65E-03	2.88E-04
	A-DGN	4.11E-05	2.44E-02	9.63E-04	1.76E-06	2.94E-02	6.83E-07
	ARMA	7.83E-03	5.12E-05	3.11E-02	8.63E-04	1.07E-02	2.55E-03
	EGC	2.56E-02	4.91E-04	6.77E-06	3.85E-02	9.41E-03	1.62E-04
	GraphGPS	8.75E-05	3.52E-02	1.43E-03	5.18E-06	2.64E-02	7.93E-05
	SSGC	1.36E-02	6.88E-04	2.75E-02	9.01E-06	3.12E-03	4.44E-02
	HGNN	2.91E-02	8.43E-05	7.11E-04	1.95E-02	5.33E-03	9.77E-06
	HGNN$^+$	7.62E-04	3.14E-02	1.25E-05	6.82E-03	2.48E-02	8.94E-07
	BScNets	1.99E-02	6.17E-06	3.87E-02	2.56E-03	4.83E-04	7.61E-03
	HiGCN	5.11E-03	1.88E-04	7.25E-06	2.74E-02	1.33E-02	4.17E-05
	MolGT	3.42E-02	7.66E-05	2.94E-03	1.22E-02	6.54E-04	8.73E-06
RMSE	MultiDRP	1.73E-02	2.14E-05	8.33E-04	3.05E-02	6.77E-06	1.92E-03
	MSDRP	4.82E-03	3.22E-02	9.11E-06	7.84E-04	2.63E-02	5.77E-05
	PANCDR	2.94E-04	6.31E-03	3.11E-02	4.26E-05	8.72E-03	1.33E-04
	GraphCDR	9.88E-03	1.74E-06	2.81E-02	6.77E-04	2.17E-03	4.01E-05
	SubCDR	5.63E-03	2.18E-04	6.55E-06	3.61E-02	8.14E-03	3.09E-04
	A-DGN	3.17E-05	2.58E-02	7.93E-04	1.22E-06	2.86E-02	4.63E-07
	ARMA	8.11E-03	4.63E-05	2.77E-02	7.45E-04	9.55E-03	3.66E-03
	EGC	2.75E-02	3.14E-04	8.12E-06	3.55E-02	6.84E-03	2.47E-04
	GraphGPS	6.88E-05	3.14E-02	1.77E-03	4.52E-06	2.45E-02	9.33E-05
	SSGC	1.44E-02	7.91E-04	2.48E-02	7.88E-06	3.22E-03	4.21E-02
	HGNN	2.66E-02	9.22E-05	6.44E-04	2.05E-02	5.92E-03	8.33E-06
	HGNN$^+$	5.92E-04	3.27E-02	1.88E-05	5.11E-03	2.14E-02	7.12E-07
	BScNets	1.85E-02	5.33E-06	3.22E-02	2.66E-03	5.11E-04	8.55E-03
	HiGCN	6.44E-03	2.47E-04	8.63E-06	2.41E-02	1.18E-02	5.77E-05
	MolGT	3.14E-02	8.12E-05	3.88E-03	1.01E-02	7.92E-04	6.11E-06
MAE	MultiDRP	1.82E-02	3.14E-05	9.11E-04	2.88E-02	5.44E-06	1.72E-03
	MSDRP	4.66E-03	3.05E-02	7.88E-06	6.77E-04	2.55E-02	4.92E-05
	PANCDR	3.11E-04	6.02E-03	2.95E-02	5.12E-05	9.33E-03	1.11E-04
	GraphCDR	9.21E-03	1.52E-06	2.66E-02	7.14E-04	2.44E-03	3.88E-05
	SubCDR	5.01E-03	2.05E-04	6.14E-06	3.44E-02	7.77E-03	3.66E-04
	A-DGN	2.88E-05	2.36E-02	8.22E-04	1.09E-06	2.77E-02	5.02E-07
	ARMA	7.74E-03	4.91E-05	2.66E-02	8.33E-04	8.44E-03	3.55E-03
	EGC	2.61E-02	3.77E-04	7.77E-06	3.22E-02	7.11E-03	2.18E-04
	GraphGPS	7.92E-05	3.02E-02	1.55E-03	5.01E-06	2.22E-02	8.66E-05
	SSGC	1.28E-02	6.55E-04	2.33E-02	8.44E-06	3.44E-03	4.09E-02
	HGNN	2.52E-02	8.33E-05	6.11E-04	2.11E-02	5.55E-03	9.11E-06
	HGNN$^+$	6.11E-04	3.11E-02	1.55E-05	5.44E-03	2.05E-02	6.88E-07
	BScNets	1.72E-02	4.91E-06	3.05E-02	2.77E-03	5.44E-04	7.99E-03
	HiGCN	6.11E-03	2.22E-04	8.11E-06	2.33E-02	1.25E-02	6.44E-05
	MolGT	3.02E-02	7.77E-05	3.55E-03	1.11E-02	7.11E-04	5.88E-06

Table S3 Pathway enrichment metrics of top-ranked 1-simplex drug pairs across four gene set libraries

Top N	Groups	Drug list	Enrichment significance			
			KEGG	GP-BP	Reactome	WikiPathways
Top 1	Group A	PLX-4720, Selumetinib	5.86	4.78	5.95	4.27
	Group B	Olaparib, Niraparib	4.30	3.16	2.78	3.45
Top 2	Group A	Irinotecan, YK-4-279	6.21	3.67	2.42	3.86
	Group B	Olaparib, Niraparib	5.28	3.69	2.86	4.06
Top 3	Group A	Dasatinib, Selumetinib	3.07	3.25	5.12	3.75
	Group B	Venetoclax, Pictilisib	1.55	1.68	1.55	1.85
Top 4	Group A	Gefitinib, Cediranib	6.34	5.15	4.36	4.72
	Group B	Niraparib, Temozolomide	1.59	1.71	1.40	1.75
Top 5	Group A	Osimertinib, Nilotinib	1.53	0.90	1.15	1.78
	Group B	Daporinad, Entinostat	1.47	1.56	1.21	1.88
			**Overlapping pathways**			
			KEGG	GP-BP	Reactome	WikiPathways
Top 1	Group A<br>Group B	PLX-4720, Selumetinib<br>Olaparib, Niraparib	15	53	31	42
Top 2	Group A<br>Group B	Irinotecan, YK-4-279<br>Olaparib, Niraparib	10	41	26	32
Top 3	Group A<br>Group B	Dasatinib, Selumetinib<br>Venetoclax, Pictilisib	49	93	32	75
Top 4	Group A<br>Group B	Gefitinib, Cediranib<br>Niraparib, Temozolomide	7	30	21	19
Top 5	Group A<br>Group B	Osimertinib, Nilotinib<br>Daporinad, Entinostat	5	17	20	6
			**Jaccard similarity**			
			KEGG	GP-BP	Reactome	WikiPathways
Top 1	Group A<br>Group B	PLX-4720, Selumetinib<br>Olaparib, Niraparib	0.139	0.156	0.215	0.227
Top 2	Group A<br>Group B	Irinotecan, YK-4-279<br>Olaparib, Niraparib	0.476	0.203	0.342	0.457
Top 3	Group A<br>Group B	Dasatinib, Selumetinib<br>Venetoclax, Pictilisib	0.308	0.106	0.086	0.267
Top 4	Group A<br>Group B	Gefitinib, Cediranib<br>Niraparib, Temozolomide	0.109	0.096	0.174	0.157
Top 5	Group A<br>Group B	Osimertinib, Nilotinib<br>Daporinad, Entinostat	0.064	0.043	0.105	0.044

Table S4 Random-baseline pathway enrichment statistics for drug simplicial interactions. For each simplex order, random simplex pairs were sampled repeatedly, and the mean enrichment significance, number of overlapping pathways, and Jaccard similarity were computed across four gene set libraries. Enrichment significance is reported as the average $-\log_{10}(\text{adjusted } p)$ over overlapping pathways.

Simplex order	Metric	KEGG	GO-BP	Reactome	WikiPathways	Average
1-simplex	Enrichment significance	0.733	1.069	1.361	1.414	1.144
	Overlapping pathways	3.900	6.480	6.160	4.880	5.355
	Jaccard similarity	0.052	0.040	0.074	0.047	0.053
2-simplex	Enrichment significance	1.229	1.879	1.477	1.495	1.520
	Overlapping pathways	5.060	9.980	7.480	5.780	7.075
	Jaccard similarity	0.078	0.039	0.062	0.058	0.059

Table S5 Representative simplex interactions with low structural similarity but strong functional enrichment. Structural similarity is computed based on molecular fingerprints (Tanimoto similarity). Despite low structural similarity, these interactions exhibit strong pathway enrichment, indicating that TopDr captures functionally meaningful relationships beyond chemical similarity.

	Case 1	Case 2	Case 3
Rank	9	17	16
Simplex Order	1-simplex	1-simplex	2-simplex
Group A	(ABT-737, Venetoclax)	(Mirdametinib, PLX-4720)	(Paclitaxel, Epirubicin, Docetaxel)
Group B	(Linsitinib, OSI-027)	(Gefitinib, Erlotinib)	(Ribociclib, BI-2536, Palbociclib)
Structural Similarity (Tanimoto)	0.148	0.143	0.119
Enrichment Significance	1.824	2.132	1.633
# Overlapping Pathways	14.5	34.5	8.0
Jaccard Similarity	0.110	0.259	0.082

Table S6 Pathway enrichment metrics of top-ranked 2-simplex drug pairs across four gene set libraries

Top N	Groups	Drug list	Enrichment significance			
			**KEGG**	**GP-BP**	**Reactome**	**WikiPathways**
Top 1	Group A	Vincristine, Vinorelbine, Vinblastine	1.74	2.26	2.36	2.05
	Group B	Ribociclib, BI 2536, Palbociclib	2.38	1.64	1.59	2.38
Top 2	Group A	Paclitaxel, Epirubicin, Docetaxel	1.45	2.01	1.86	2.00
	Group B	Vincristine, Irinotecan, Vinorelbine	2.00	1.88	1.38	2.22
Top 3	Group A	Lapatinib, Erlotinib, AZD-3759	4.78	4.48	4.90	4.90
	Group B	Zoledronic acid, Cyclophosphamide, Fluorouracil	4.48	3.50	4.89	3.91
Top 4	Group A	Paclitaxel, Epirubicin, Docetaxel	1.47	1.95	1.80	2.03
	Group B	Vincristine, Vinorelbine, Cediranib	1.94	1.83	1.36	2.16
Top 5	Group A	Olaparib, Talazoparib, Fluorouracil	3.98	3.30	4.34	3.83
	Group B	ABT-737, Venetoclax, Navitoclax	2.55	1.88	1.36	2.01
			**Overlapping pathways**			
			**KEGG**	**GP-BP**	**Reactome**	**WikiPathways**
Top 1	Group A<br>Group B	Vincristine, Vinorelbine, Vinblastine<br>Ribociclib, BI 2536, Palbociclib	37	68	48	50
Top 2	Group A<br>Group B	Paclitaxel, Epirubicin, Docetaxel<br>Vincristine, Irinotecan, Vinorelbine	69	124	57	91
Top 3	Group A<br>Group B	Lapatinib, Erlotinib, AZD-3759<br>Zoledronic acid, Cyclophosphamide, Fluorouracil	17	60	31	43
Top 4	Group A<br>Group B	Paclitaxel, Epirubicin, Docetaxel<br>Vincristine, Vinorelbine, Cediranib	66	124	50	87
Top 5	Group A<br>Group B	Olaparib, Talazoparib, Fluorouracil<br>ABT-737, Venetoclax, Navitoclax	17	50	24	38
			**Jaccard similarity**			
			**KEGG**	**GP-BP**	**Reactome**	**WikiPathways**
Top 1	Group A<br>Group B	Vincristine, Vinorelbine, Vinblastine<br>Ribociclib, BI 2536, Palbociclib	0.257	0.179	0.189	0.187
Top 2	Group A<br>Group B	Paclitaxel, Epirubicin, Docetaxel<br>Vincristine, Irinotecan, Vinorelbine	0.476	0.347	0.237	0.376
Top 3	Group A<br>Group B	Lapatinib, Erlotinib, AZD-3759<br>Zoledronic acid, Cyclophosphamide, Fluorouracil	0.239	0.155	0.226	0.309
Top 4	Group A<br>Group B	Paclitaxel, Epirubicin, Docetaxel<br>Vincristine, Vinorelbine, Cediranib	0.452	0.289	0.206	0.348
Top 5	Group A<br>Group B	Olaparib, Talazoparib, Fluorouracil<br>ABT-737, Venetoclax, Navitoclax	0.160	0.106	0.158	0.197

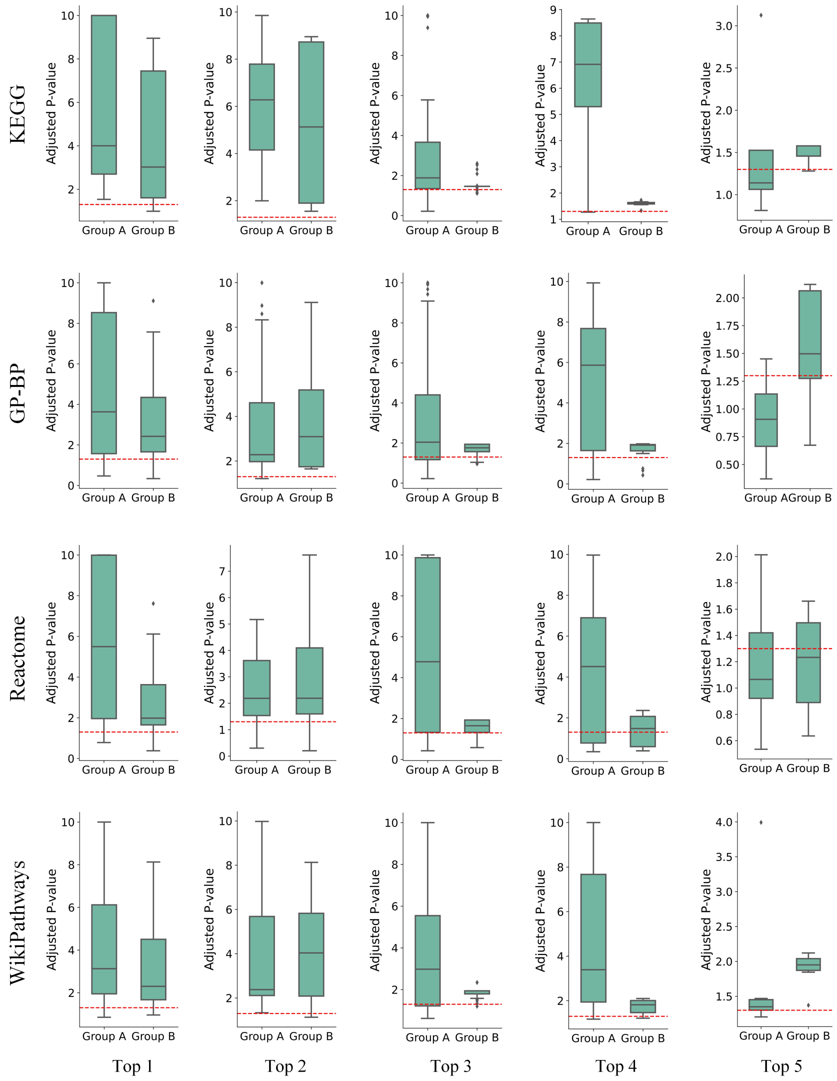

Fig. S1 Box plots of enrichment significance (adjusted p-values) across top-ranked 1-simplex drug pairs on the TGSA dataset. Each row corresponds to one of the top five 1-simplex drug pair groups (Top 1–Top 5) ranked by attention scores, and each column represents a distinct pathway database (KEGG, GO Biological Process, Reactome, WikiPathways). Box plots show the distribution of adjusted p-values for Group A and Group B in each drug pair. The red dashed line marks the threshold for statistical significance (adjusted p-value = 1.3, corresponding to p = 0.05).

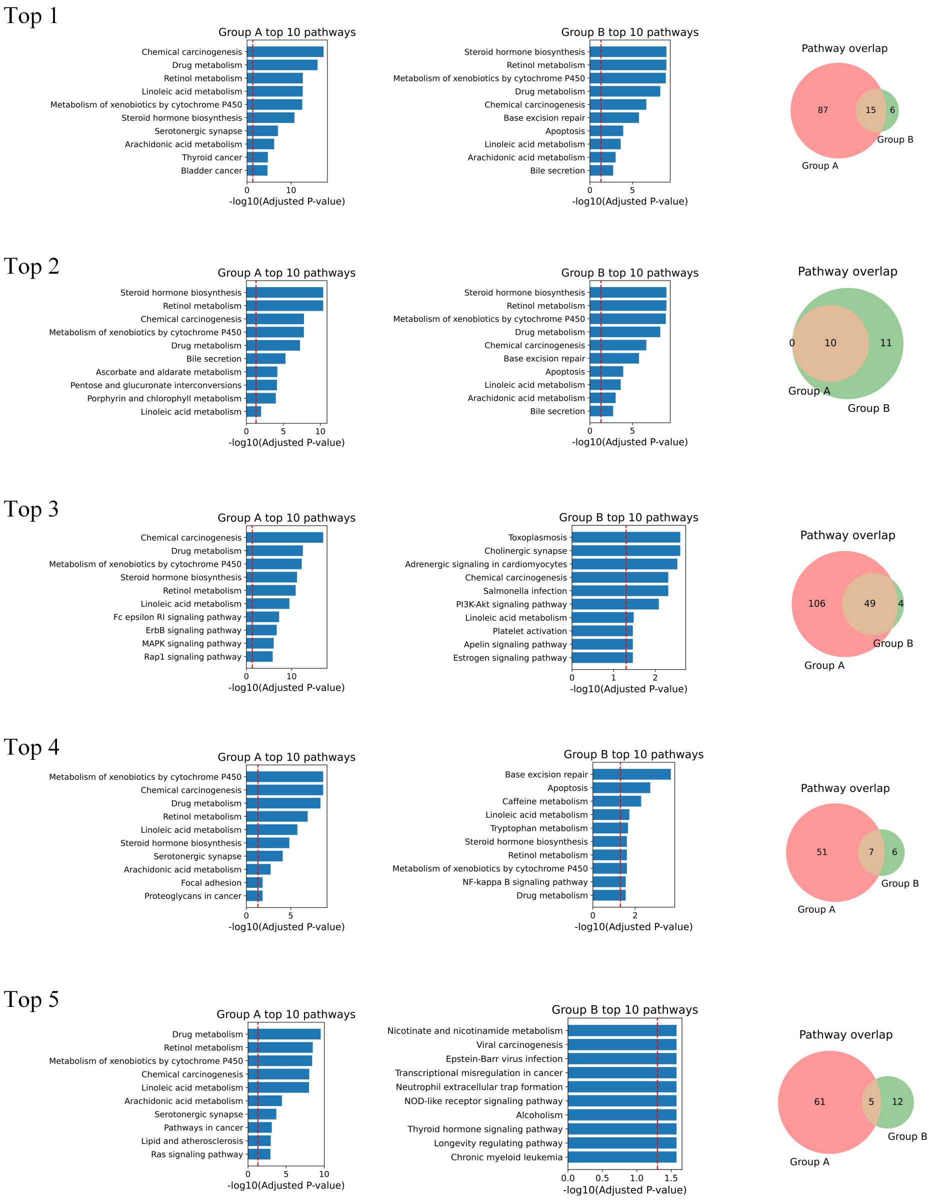

Fig. S2 Top enriched KEGG pathways of top-ranked 1-simplex drug pairs in the TGSA dataset. The top five 1-simplex drug pairs with the highest attention scores were selected from the attention matrix learned on the TGSA dataset. For each pair, pathway enrichment analysis was performed on drug targets using the KEGG gene set, and the top 10 enriched pathways (ranked by adjusted p-value) were shown for both Group A and Group B. The red dashed line indicates the statistical significance threshold (adjusted p-value = 1.3, corresponding to $p = 0.05$). The rightmost column presents Venn diagrams illustrating the overlap of enriched pathways between the two groups.

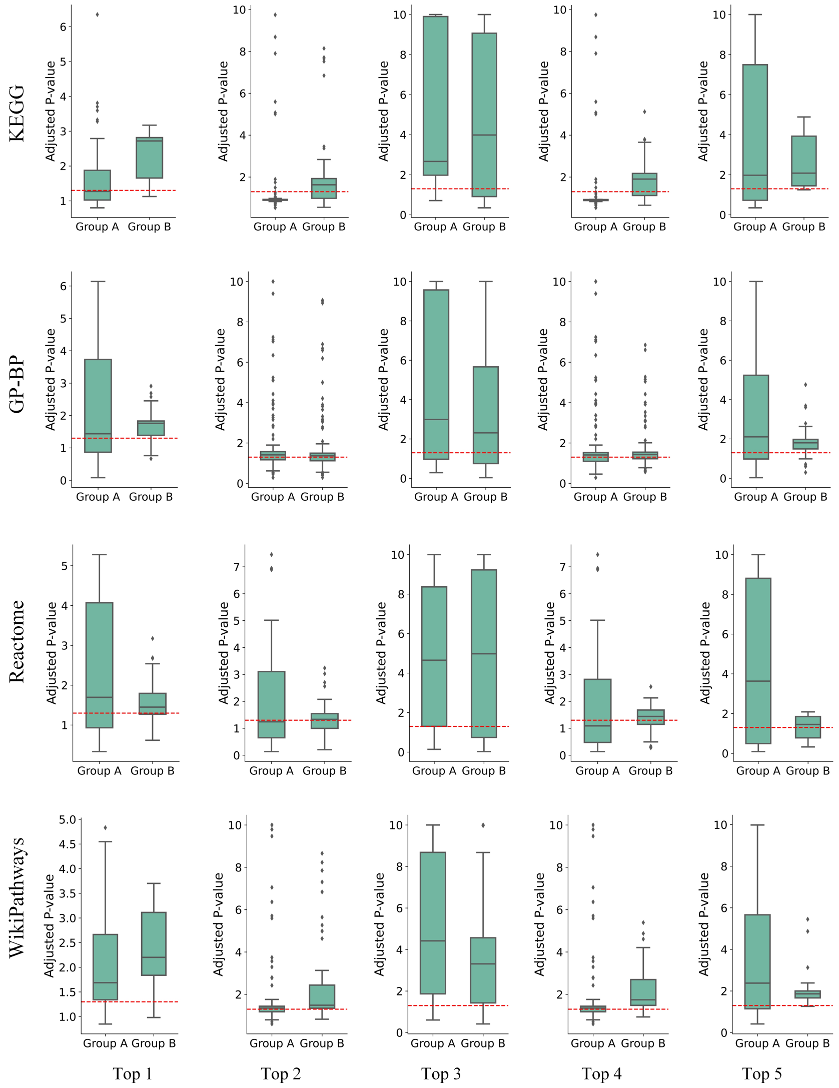

Fig. S3 Box plots of enrichment significance (adjusted p-values) across top-ranked 2-simplex drug pairs on the TGSA dataset. Each row corresponds to one of the top five 2-simplex drug pair groups (Top 1–Top 5) ranked by attention scores, and each column represents a distinct pathway database (KEGG, GO Biological Process, Reactome, WikiPathways). Box plots show the distribution of adjusted p-values for Group A and Group B in each drug pair. The red dashed line marks the threshold for statistical significance (adjusted p-value = 1.3, corresponding to p = 0.05).

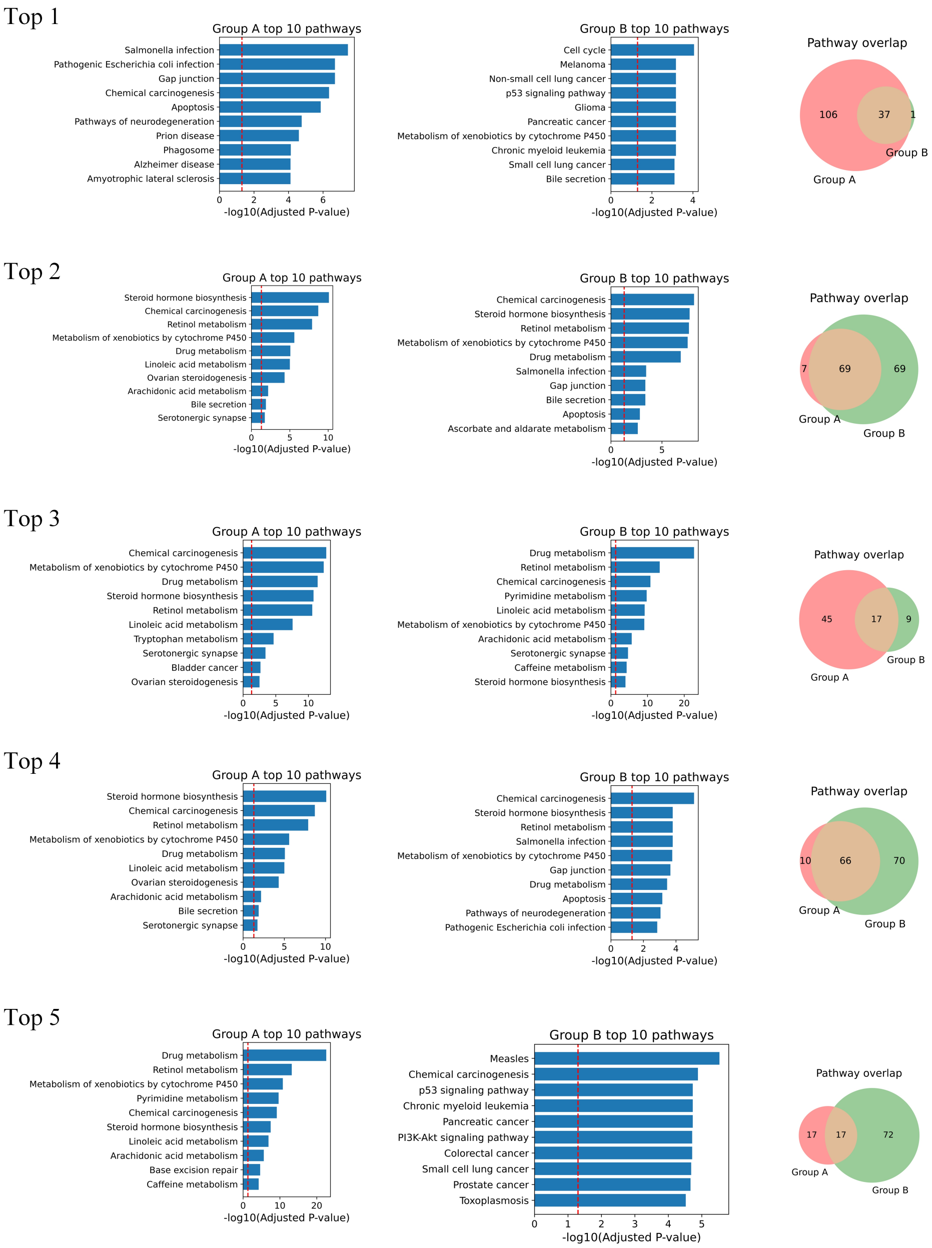

Fig. S4 Top enriched KEGG pathways of top-ranked 2-simplex drug pairs in the TGSA dataset. The top five 2-simplex drug pairs with the highest attention scores were selected from the attention matrix learned on the TGSA dataset. For each pair, pathway enrichment analysis was performed on drug targets using the KEGG gene set, and the top 10 enriched pathways (ranked by adjusted p-value) were shown for both Group A and Group B. The red dashed line indicates the statistical significance threshold (adjusted p-value = 1.3, corresponding to $p = 0.05$). The rightmost column presents Venn diagrams illustrating the overlap of enriched pathways between the two groups.

Table S7 Pathway enrichment metrics of top-ranked 1-simplex cell line pairs across four gene set libraries

Top N	Groups	Cell line list	Enrichment significance			
			**KEGG**	**GP-BP**	**Reactome**	**WikiPathways**
Top 1	Group A	TYK-nu, LU-65	1.15	1.32	0.95	0.96
	Group B	Hs-746T, SNG-M	1.05	1.51	1.06	1.27
Top 2	Group A	HCC38, A427	1.36	1.39	1.31	0.98
	Group B	HEC-1, HO-1-u-1	1.88	1.79	1.80	1.61
Top 3	Group A	HuH-7, TOV-21G	1.25	1.74	1.17	2.15
	Group B	Hep3B2-1-7, GOTO	1.24	1.60	1.06	1.40
Top 4	Group A	SF539, TE-4	1.15	1.39	1.54	1.12
	Group B	MDA-MB-468, 8505C	1.31	1.60	1.10	1.93
Top 5	Group A	U-87-MG, KALS-1	1.30	1.44	1.34	1.16
	Group B	LXF-289, A2058	2.10	1.71	1.76	1.37
			**Overlapping pathways**			
			**KEGG**	**GP-BP**	**Reactome**	**WikiPathways**
Top 1	Group A<br>Group B	TYK-nu, LU-65<br>Hs-746T, SNG-M	7	121	42	5
Top 2	Group A<br>Group B	HCC38, A427<br>HEC-1, HO-1-u-1	23	232	97	8
Top 3	Group A<br>Group B	HuH-7, TOV-21G<br>Hep3B2-1-7, GOTO	9	116	69	5
Top 4	Group A<br>Group B	SF539, TE-4<br>MDA-MB-468, 8505C	5	110	39	4
Top 5	Group A<br>Group B	U-87-MG, KALS-1<br>LXF-289, A2058	20	236	93	16
			**Jaccard similarity**			
			**KEGG**	**GP-BP**	**Reactome**	**WikiPathways**
Top 1	Group A<br>Group B	TYK-nu, LU-65<br>Hs-746T, SNG-M	0.135	0.301	0.304	0.172
Top 2	Group A<br>Group B	HCC38, A427<br>HEC-1, HO-1-u-1	0.719	0.554	0.497	0.222
Top 3	Group A<br>Group B	HuH-7, TOV-21G<br>Hep3B2-1-7, GOTO	0.118	0.372	0.375	0.161
Top 4	Group A<br>Group B	SF539, TE-4<br>MDA-MB-468, 8505C	0.083	0.272	0.205	0.071
Top 5	Group A<br>Group B	U-87-MG, KALS-1<br>LXF-289, A2058	0.625	0.576	0.517	0.444

Table S8 Pathway enrichment metrics of top-ranked 2-simplex cell line pairs across four gene set libraries

Top N	Groups	Cell line list	Enrichment significance			
			**KEGG**	**GP-BP**	**Reactome**	**WikiPathways**
Top 1	Group A	NCI-H1693, IST-MES1, SNU-C1	1.93	1.85	2.13	1.67
	Group B	HSC-4, LS-180, SW982	1.17	1.49	1.27	1.27
Top 2	Group A	MFE-280, SW900, SNG-M	1.12	1.45	1.42	1.43
	Group B	BHT-101, NCI-H3122, HCC202	0.99	1.46	1.23	1.07
Top 3	Group A	Calu-6, HCC70, OC-314	0.95	1.44	1.26	1.24
	Group B	CAPAN-2, TYK-nu, LU-65	1.05	1.35	1.15	1.06
Top 4	Group A	CFPAC-1, NCI-H211, HCC1569	1.51	1.36	1.48	1.10
	Group B	RD, NCI-H209, IST-MES1	1.70	1.67	1.80	1.46
Top 5	Group A	NCI-H69, SNU-423, NCI-H1623	1.58	1.52	1.64	1.33
	Group B	TYK-nu, LU-65, FLO-1	1.10	1.37	1.19	0.97
			**Overlapping pathways**			
			**KEGG**	**GP-BP**	**Reactome**	**WikiPathways**
Top 1	Group A<br>Group B	NCI-H1693, IST-MES1, SNU-C1<br>HSC-4, LS-180, SW982	26	256	124	12
Top 2	Group A<br>Group B	MFE-280, SW900, SNG-M<br>BHT-101, NCI-H3122, HCC202	27	231	126	11
Top 3	Group A<br>Group B	Calu-6, HCC70, OC-314<br>CAPAN-2, TYK-nu, LU-65	27	236	106	12
Top 4	Group A<br>Group B	CFPAC-1, NCI-H211, HCC1569<br>RD, NCI-H209, IST-MES1	27	264	121	12
Top 5	Group A<br>Group B	NCI-H69, SNU-423, NCI-H1623<br>TYK-nu, LU-65, FLO-1	26	255	102	9
			**Jaccard similarity**			
			**KEGG**	**GP-BP**	**Reactome**	**WikiPathways**
Top 1	Group A<br>Group B	NCI-H1693, IST-MES1, SNU-C1<br>HSC-4, LS-180, SW982	0.366	0.821	0.642	0.308
Top 2	Group A<br>Group B	MFE-280, SW900, SNG-M<br>BHT-101, NCI-H3122, HCC202	0.290	0.562	0.516	0.169
Top 3	Group A<br>Group B	Calu-6, HCC70, OC-314<br>CAPAN-2, TYK-nu, LU-65	0.818	0.589	0.582	0.429
Top 4	Group A<br>Group B	CFPAC-1, NCI-H211, HCC1569<br>RD, NCI-H209, IST-MES1	0.252	0.523	0.465	0.154
Top 5	Group A<br>Group B	NCI-H69, SNU-423, NCI-H1623<br>TYK-nu, LU-65, FLO-1	0.329	0.560	0.507	0.170

Table S9 Performance Comparison under Perturbed Molecular Fingerprints (20% Random Noise)

Metric	Methods	TGSA	GDSC1	GDSC2	CCLE	CTRP1	CTRP2
PCC	MultiDRP	$0.9323_{(0.004)}$	$0.9239_{(0.007)}$	$\mathbf{0.9409}_{(0.004)}$	$0.8761_{(0.003)}$	$0.8063_{(0.004)}$	$0.8992_{(0.005)}$
	MSDRP	$0.9088_{(0.002)}$	$0.9237_{(0.004)}$	$0.9358_{(0.005)}$	$0.8683_{(0.002)}$	$0.8338_{(0.003)}$	$0.8926_{(0.004)}$
	A-DGN	$0.9220_{(0.007)}$	$0.9148_{(0.010)}$	$0.9299_{(0.008)}$	$0.8706_{(0.001)}$	$0.7956_{(0.007)}$	$0.8749_{(0.008)}$
	ARMA	$0.8760_{(0.011)}$	$0.9295_{(0.007)}$	$0.9351_{(0.003)}$	$0.8760_{(0.002)}$	$0.7976_{(0.007)}$	$0.8962_{(0.008)}$
	EGC	$0.9299_{(0.005)}$	$0.9268_{(0.003)}$	$0.9349_{(0.004)}$	$0.8733_{(0.007)}$	$0.7901_{(0.004)}$	$0.8944_{(0.002)}$
	GraphGPS	$0.9334_{(0.003)}$	$0.9293_{(0.002)}$	$0.9382_{(0.006)}$	$0.8792_{(0.014)}$	$0.8206_{(0.003)}$	$0.8992_{(0.005)}$
	SSGC	$0.9249_{(0.005)}$	$0.9124_{(0.007)}$	$0.9290_{(0.007)}$	$0.8729_{(0.008)}$	$0.8012_{(0.004)}$	$0.8791_{(0.006)}$
	HGNN	$0.9017_{(0.002)}$	$0.8857_{(0.006)}$	$0.9006_{(0.003)}$	$0.8484_{(0.006)}$	$0.6158_{(0.008)}$	$0.8563_{(0.003)}$
	HGNN$^+$	$0.8248_{(0.008)}$	$0.8204_{(0.012)}$	$0.7682_{(0.005)}$	$0.7465_{(0.003)}$	$0.4437_{(0.008)}$	$0.8131_{(0.004)}$
	BScNets	$0.9038_{(0.007)}$	$0.8898_{(0.006)}$	$0.9059_{(0.008)}$	$0.8725_{(0.004)}$	$0.7316_{(0.004)}$	$0.8462_{(0.004)}$
	HiGCN	$0.8884_{(0.002)}$	$0.8975_{(0.007)}$	$0.9114_{(0.002)}$	$0.8661_{(0.005)}$	$0.7045_{(0.005)}$	$0.8533_{(0.007)}$
	TopDr	$\mathbf{0.9401}_{(0.003)}$	$\mathbf{0.9306}_{(0.006)}$	$0.9397_{(0.003)}$	$\mathbf{0.8785}_{(0.002)}$	$\mathbf{0.8256}_{(0.005)}$	$\mathbf{0.9006}_{(0.004)}$
RMSE	MultiDRP	$1.0252_{(0.022)}$	$1.0257_{(0.018)}$	$0.9659_{(0.041)}$	$0.7551_{(0.025)}$	$1.0133_{(0.014)}$	$1.1495_{(0.022)}$
	MSDRP	$1.1868_{(0.026)}$	$1.0195_{(0.022)}$	$1.0014_{(0.033)}$	$0.7820_{(0.034)}$	$0.9521_{(0.010)}$	$1.1913_{(0.017)}$
	A-DGN	$1.0991_{(0.017)}$	$1.0852_{(0.023)}$	$1.0499_{(0.020)}$	$0.7741_{(0.017)}$	$1.0383_{(0.021)}$	$1.2781_{(0.029)}$
	ARMA	$0.9944_{(0.019)}$	$0.9879_{(0.037)}$	$1.0118_{(0.042)}$	$0.7544_{(0.026)}$	$1.0317_{(0.027)}$	$1.1735_{(0.018)}$
	EGC	$1.0432_{(0.021)}$	$1.0071_{(0.031)}$	$1.0139_{(0.039)}$	$0.7899_{(0.018)}$	$1.0514_{(0.025)}$	$1.1760_{(0.020)}$
	GraphGPS	$1.0177_{(0.022)}$	$0.9894_{(0.014)}$	$0.9881_{(0.027)}$	$0.7570_{(0.051)}$	$0.9769_{(0.017)}$	$1.1504_{(0.035)}$
	SSGC	$1.0789_{(0.025)}$	$1.0959_{(0.027)}$	$1.0575_{(0.016)}$	$0.7651_{(0.012)}$	$1.0234_{(0.024)}$	$1.2554_{(0.030)}$
	HGNN	$1.2263_{(0.018)}$	$1.2360_{(0.022)}$	$1.2382_{(0.024)}$	$0.8341_{(0.022)}$	$1.3609_{(0.008)}$	$1.3640_{(0.016)}$
	HGNN$^+$	$1.6047_{(0.046)}$	$1.5285_{(0.021)}$	$1.8211_{(0.035)}$	$1.0488_{(0.018)}$	$1.5496_{(0.033)}$	$1.5493_{(0.018)}$
	BScNets	$1.2161_{(0.050)}$	$1.2156_{(0.027)}$	$1.2069_{(0.012)}$	$0.7704_{(0.014)}$	$1.1747_{(0.038)}$	$1.4084_{(0.040)}$
	HiGCN	$1.3389_{(0.032)}$	$1.1745_{(0.019)}$	$1.1771_{(0.030)}$	$0.7895_{(0.025)}$	$1.2481_{(0.039)}$	$1.3885_{(0.015)}$
	TopDr	$\mathbf{0.9635}_{(0.031)}$	$\mathbf{0.9788}_{(0.024)}$	$\mathbf{0.9771}_{(0.041)}$	$\mathbf{0.7513}_{(0.021)}$	$\mathbf{0.9671}_{(0.018)}$	$\mathbf{1.1402}_{(0.034)}$
MAE	MultiDRP	$0.7617_{(0.022)}$	$0.7642_{(0.019)}$	$0.7139_{(0.017)}$	$0.4705_{(0.018)}$	$0.7512_{(0.011)}$	$0.8045_{(0.021)}$
	MSDRP	$0.8965_{(0.018)}$	$0.7582_{(0.006)}$	$0.7452_{(0.010)}$	$0.4783_{(0.012)}$	$0.6980_{(0.026)}$	$0.8276_{(0.015)}$
	A-DGN	$0.8220_{(0.011)}$	$0.8146_{(0.022)}$	$0.7875_{(0.005)}$	$0.4843_{(0.028)}$	$0.7885_{(0.024)}$	$0.9230_{(0.018)}$
	ARMA	$0.7721_{(0.013)}$	$0.7312_{(0.014)}$	$0.7535_{(0.012)}$	$0.4721_{(0.031)}$	$0.7732_{(0.013)}$	$0.8307_{(0.011)}$
	EGC	$0.7808_{(0.010)}$	$0.7459_{(0.014)}$	$0.7550_{(0.031)}$	$0.4805_{(0.014)}$	$0.7885_{(0.014)}$	$0.8332_{(0.031)}$
	GraphGPS	$0.7591_{(0.009)}$	$0.7393_{(0.018)}$	$0.7374_{(0.024)}$	$0.4702_{(0.021)}$	$0.7339_{(0.012)}$	$0.8161_{(0.027)}$
	SSGC	$0.8113_{(0.016)}$	$0.8217_{(0.022)}$	$0.7915_{(0.016)}$	$0.4817_{(0.028)}$	$0.7738_{(0.022)}$	$0.8951_{(0.014)}$
	HGNN	$0.9171_{(0.013)}$	$0.9290_{(0.011)}$	$0.9376_{(0.018)}$	$0.5050_{(0.022)}$	$1.0598_{(0.025)}$	$0.9516_{(0.025)}$
	HGNN$^+$	$1.2531_{(0.019)}$	$1.1639_{(0.016)}$	$1.4146_{(0.015)}$	$0.5996_{(0.014)}$	$1.2362_{(0.022)}$	$1.1280_{(0.013)}$
	BScNets	$0.9158_{(0.015)}$	$0.9063_{(0.013)}$	$0.9042_{(0.016)}$	$0.4721_{(0.015)}$	$0.8891_{(0.016)}$	$0.9853_{(0.014)}$
	HiGCN	$1.0353_{(0.021)}$	$0.8735_{(0.011)}$	$0.8790_{(0.012)}$	$0.4823_{(0.007)}$	$0.9719_{(0.018)}$	$0.9712_{(0.021)}$
	TopDr	$\mathbf{0.7208}_{(0.001)}$	$\mathbf{0.7215}_{(0.001)}$	$\mathbf{0.7221}_{(0.001)}$	$\mathbf{0.4699}_{(0.001)}$	$\mathbf{0.7142}_{(0.001)}$	$\mathbf{0.7988}_{(0.001)}$

Table S10 Performance comparison under cross-cell line and cross-drug settings.

Setting	Metric	Method	TGSA	GDSC1	GDSC2	CCLE	CTRP1	CTRP2
Cross-cell	PCC	TopDr	0.8072	0.8565	0.9370	0.8584	0.6394	0.7538
		MultiDRP	0.7941	0.8442	0.8336	0.8400	0.5871	0.7462
	RMSE	TopDr	1.7090	1.4232	1.6019	0.8783	1.3627	1.7923
		MultiDRP	1.7344	1.4801	1.6649	0.9185	1.4162	1.8272
	MAE	TopDr	1.2605	1.0610	1.2469	0.4951	1.0643	1.2581
		MultiDRP	1.3159	1.1142	1.2674	0.5365	1.0926	1.3044
Cross-drug	PCC	TopDr	0.4217	0.4180	0.6855	0.4500	0.3622	0.3894
		MultiDRP	0.3925	0.4076	0.6673	0.4365	0.3588	0.3674
	RMSE	TopDr	2.2170	1.9595	1.9946	1.0310	1.8939	3.0689
		MultiDRP	2.3513	2.0489	2.1294	1.2139	2.0012	3.6905
	MAE	TopDr	1.5358	1.5734	1.5923	0.8391	1.5981	2.2655
		MultiDRP	1.6987	1.7921	1.6881	0.9128	1.7283	2.3589

Table S11 Parameter sensitivity analysis with respect to the number of nearest neighbors k in kNN-based construction.

Metric	k	TGSA	GDSC1	GDSC2	CCLE	CTRP1	CTRP2
PCC	$k=5$	0.9407	0.9297	0.9380	0.8781	0.8224	0.8950
	$k=10$	0.9405	0.9291	0.9394	0.8758	0.8256	0.8887
	$k=15$	0.9389	0.9266	0.9366	0.8746	0.8214	0.8979
	$k=20$	0.9387	0.9259	0.9374	0.8789	0.8173	0.8951
RMSE	$k=5$	0.9602	0.9885	0.9879	0.7488	0.9811	1.1728
	$k=10$	0.9663	0.9923	0.9785	0.7581	0.9676	1.2046
	$k=15$	0.9778	1.0069	0.9986	0.7600	0.9816	1.1557
	$k=20$	0.9756	1.0124	0.9923	0.7476	0.9936	1.1720
MAE	$k=5$	0.7117	0.7309	0.7331	0.4684	0.7158	0.8196
	$k=10$	0.7135	0.7366	0.7308	0.4725	0.7104	0.8398
	$k=15$	0.7203	0.7488	0.7434	0.4735	0.7275	0.8080
	$k=20$	0.7157	0.7494	0.7366	0.4680	0.7293	0.8199

Table S12 Sensitivity analysis of the RBF center range c_l for 1-simplex feature initialization.

Metric	c_l range	TGSA	GDSC1	GDSC2	CCLE	CTRP1	CTRP2
PCC	[0,2]	0.9390	0.9320	0.9405	0.8772	0.8186	0.9037
	[0,4]	0.9388	0.9282	0.9417	0.8791	0.8236	0.8979
	[0,6]	0.9406	0.9279	0.9408	0.8788	0.8157	0.9009
	[0,8]	0.9407	0.9322	0.9406	0.8779	0.8308	0.9024
	[0,10]	0.9418	0.9318	0.9423	0.8791	0.8319	0.9026
RMSE	[0,2]	0.9761	0.9730	0.9688	0.7524	0.9844	1.1255
	[0,4]	0.9761	0.9826	0.9589	0.7453	0.9756	1.1664
	[0,6]	0.9653	0.9849	0.9671	0.7480	0.9904	1.1398
	[0,8]	0.9600	0.9713	0.9681	0.7487	0.9560	1.1338
	[0,10]	0.9484	0.9762	0.9608	0.7454	0.9562	1.1331
MAE	[0,2]	0.7213	0.7197	0.7159	0.4678	0.7221	0.7835
	[0,4]	0.7157	0.7268	0.7089	0.4678	0.7170	0.8128
	[0,6]	0.7134	0.7276	0.7149	0.4683	0.7283	0.7939
	[0,8]	0.7185	0.7179	0.7182	0.4668	0.7044	0.7875
	[0,10]	0.7114	0.7187	0.7109	0.4671	0.7058	0.7900

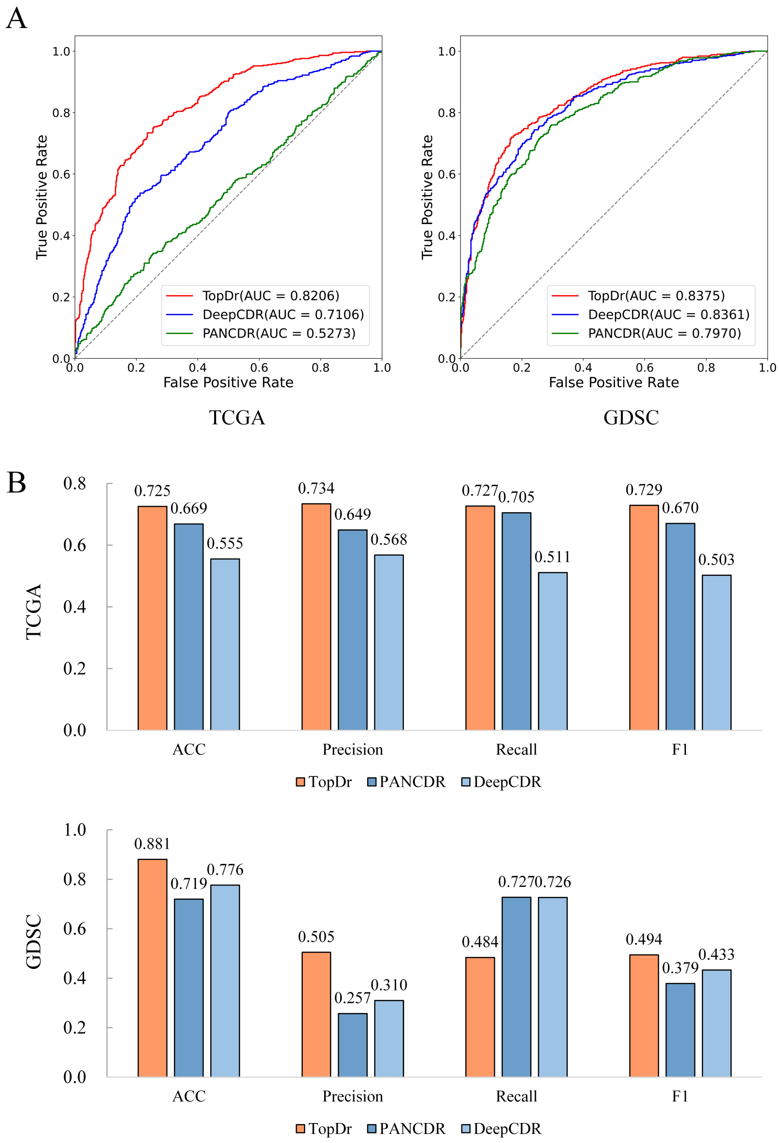

Fig. S5 Performance comparison on discrete drug response phenotype prediction. (A) ROC curves showing AUC for TopDr, PANCDR, and DeepCDR on TCGA and GDSC datasets. **(B)** Bar plots of classification metrics (ACC, Precision, Recall, and F1 score) across the three models on TCGA (top) and GDSC (bottom).

Table S13 Sensitivity analysis of the RBF center range c_l for 2-simplex feature initialization.

Metric	c_l range	TGSA	GDSC1	GDSC2	CCLE	CTRP1	CTRP2
PCC	[0,2]	0.9415	0.9314	0.9406	0.8766	0.8224	0.9004
	[0,4]	0.9418	0.9318	0.9423	0.8791	0.8319	0.9026
	[0,6]	0.9398	0.9316	0.9406	0.8778	0.8277	0.9003
	[0,8]	0.9409	0.9305	0.9417	0.8759	0.8185	0.9420
	[0,10]	0.9405	0.9310	0.9413	0.8775	0.8305	0.8991
RMSE	[0,2]	0.9553	0.9776	0.9714	0.7522	0.9806	1.1421
	[0,4]	0.9484	0.9762	0.9608	0.7454	0.9562	1.1331
	[0,6]	0.9680	0.9741	0.9679	0.7502	0.9696	1.1443
	[0,8]	0.9622	0.9809	0.9601	0.7556	0.9956	0.9626
	[0,10]	0.9663	0.9783	0.9634	0.7559	0.9558	1.1506
MAE	[0,2]	0.7139	0.7214	0.7186	0.4686	0.7130	0.7949
	[0,4]	0.7114	0.7187	0.7109	0.4671	0.7058	0.7900
	[0,6]	0.7105	0.7172	0.7215	0.4693	0.7092	0.7973
	[0,8]	0.7159	0.7242	0.7085	0.4709	0.7337	0.7128
	[0,10]	0.7135	0.7227	0.7174	0.4720	0.7110	1.3239

Table S14 Sensitivity analysis of the RBF step size in feature initialization.

Metric	Step	TGSA	GDSC1	GDSC2	CCLE	CTRP1	CTRP2
PCC	0.05	0.9408	0.9310	0.9417	0.8782	0.8262	0.9027
	0.1	0.9418	0.9318	0.9423	0.8791	0.8319	0.9026
	0.2	0.9403	0.9314	0.9419	0.8748	0.8307	0.8995
	0.3	0.9415	0.9306	0.9413	0.8770	0.8183	0.9022
	0.4	0.9408	0.9318	0.9417	0.8790	0.8212	0.9026
	0.5	0.9406	0.9294	0.9411	0.8804	0.8266	0.9032
RMSE	0.05	0.9624	0.9790	0.9603	0.7480	0.9710	1.1350
	0.1	0.9484	0.9762	0.9608	0.7454	0.9562	1.1331
	0.2	0.9639	0.9752	0.9577	0.7592	0.9590	1.1501
	0.3	0.9558	0.9810	0.9633	0.7520	0.9856	1.1357
	0.4	0.9617	0.9731	0.9614	0.7521	0.9825	1.1285
	0.5	0.9561	0.9736	0.9656	0.7416	0.9725	1.1269
MAE	0.05	0.7057	0.7235	0.7090	0.4657	0.7167	0.7898
	0.1	0.7114	0.7187	0.7109	0.4671	0.7058	0.7900
	0.2	0.7139	0.7203	0.7115	0.4716	0.7023	0.8028
	0.3	0.7101	0.7265	0.7128	0.4727	0.7204	0.7950
	0.4	0.7106	0.7182	0.7118	0.4708	0.7278	0.7895
	0.5	0.7132	0.7240	0.7134	0.4653	0.7224	0.7885

Table S15 Summary of drug response statistics across six pharmacogenomic datasets

Dataset	Number of Drugs	Number of Cell Lines	Number of Drug Responses
TGSA	170	580	82,833
GDSC1	312	946	271,705
GDSC2	233	944	198,457
CCLE	1267	221	278,597
CTRP1	203	240	30,194
CTRP2	545	821	364,774
